# C3d-Targeted factor H inhibits tissue complement in disease models and reduces glomerular injury without affecting circulating complement

**DOI:** 10.1016/j.ymthe.2024.02.001

**Published:** 2024-02-20

**Authors:** Fei Liu, Sarah T. Ryan, Kelly C. Fahnoe, Jennifer G. Morgan, Anne E. Cheung, Michael J. Storek, Alejandro Best, Hui A. Chen, Monica Locatelli, Shuyun Xu, Enno Schmidt, Leon F. Schmidt-Jiménez, Katja Bieber, Joel M. Henderson, Christine G. Lian, Admar Verschoor, Ralf J. Ludwig, Ariela Benigni, Giuseppe Remuzzi, David J. Salant, Susan L. Kalled, Joshua M. Thurman, V. Michael Holers, Shelia M. Violette, Stefan Wawersik

**Affiliations:** 1 Q32 Bio, Waltham, MA 02451, USA; 2 Arkana Laboratories, Little Rock, AR 77211, USA; 3 Department of Pathology and Laboratory Medicine, Chobanian and Avedisian School of Medicine at Boston University and Boston Medical Center, Boston, MA 02118, USA; 4 Istituto di Ricerche Farmacologiche Mario Negri IRCCS, Centro Anna Maria Astori, Science and Technology Park Kilometro Rosso, 24126 Bergamo, Italy; 5 Department of Pathology, Brigham & Women’s Hospital/Harvard Medical School, Boston, MA 02115, USA; 6 Lübeck Institute of Experimental Dermatology, University of Lübeck, 23562 Lübeck, Germany; 7 Department of Otorhinolaryngology, Technische Universität München and Klinikum Rechts der Isar, 81675 Munich, Germany; 8 Department of Dermatology, University Hospital Schleswig-Holstein, University of Lübeck, 23562 Lübeck, Germany; 9 Department of Medicine, Chobanian and Avedisian School of Medicine at Boston University and Section of Nephrology, Boston Medical Center, Boston, MA 02118, USA; 10 Department of Medicine, University of Colorado School of Medicine, Anschutz Medical Campus, Aurora, CO 80045, USA

**Keywords:** complement, tissue-targeted, factor H, C3d, glomerular disease, skin disease

## Abstract

Complement-mediated diseases can be treated using systemic inhibitors. However, complement components are abundant in circulation, affecting systemic inhibitors’ exposure and efficacy. Furthermore, because of complement’s essential role in immunity, systemic treatments raise infection risk in patients. To address these challenges, we developed antibody fusion proteins combining the alternative-pathway complement inhibitor factor H (fH_1–5_) with an anti-C3d monoclonal antibody (C3d-mAb-2fH). Because C3d is deposited at sites of complement activity, this molecule localizes to tissue complement while minimizing circulating complement engagement. These fusion proteins bind to deposited complement in diseased human skin sections and localize to activated complement in a primate skin injury model. We further explored the pharmacology of C3d-mAb-2fH proteins in rodent models with robust tissue complement activation. Doses of C3d-mAb-2fH >1 mg/kg achieved >75% tissue complement inhibition in mouse and rat injury models while avoiding circulating complement blockade. Glomerular-specific complement inhibition reduced proteinuria and preserved podocyte foot-process architecture in rat membranous nephropathy, indicating disease-modifying efficacy. These data indicate that targeting local tissue complement results in durable and efficacious complement blockade in skin and kidney while avoiding systemic inhibition, suggesting broad applicability of this approach in treating a range of complement-mediated diseases.

## Graphical abstract

**Figure:**
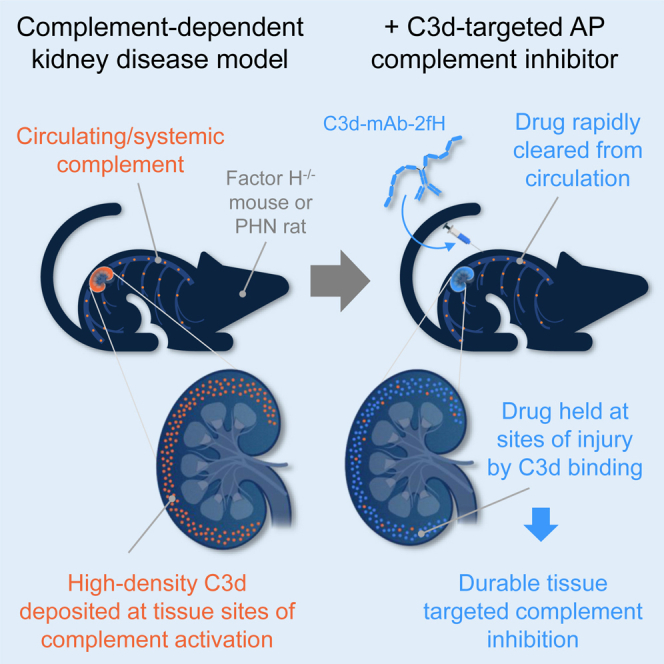

## Teaser

Liu, Ryan and colleagues show that C3d-targeted fusion proteins locally inhibit dysregulated complement in tissues and can reduce disease progression in preclinical models. This efficacy can be achieved with minimal effects on circulating complement, potentially offering a novel, potent, and targeted approach to therapeutic re-regulation of complement in autoimmune disease.

## Introduction

Complement is an essential component of immunity, providing a first line of defense against pathogens and a bridge between the innate and adaptive immune systems.^1^^,^^2^^,^^3^ The complement cascade can be initiated through either mannose-binding lectins (lectin pathway [LP]) or by immunoglobulin M (IgM) or IgG clustering (classical pathway [CP]).^3^ A third arm, the alternative pathway (AP), amplifies CP- and LP-initiated complement activation.^4^^,^^5^^,^^6^^,^^7^ All three pathways trigger formation of protein complexes called convertases that proteolytically cleave complement C3 and C5 proteins into functional fragments. Complement activation drives multiple important immune and homeostatic functions. These include promoting cell lysis and pro-inflammatory signaling through membrane attack complex (MAC) formation, activating phagocytosis through target opsonization, attracting phagocytes by generating C3a and C5a chemotactic peptides, increased expression of C3a and C5a receptors on selected cell types, and stimulation of B cells, T cells, and follicular dendritic cells.^1^^,^^2^^,^^3^^,^^8^

Persistent uncontrolled complement activation plays a major role in the pathogenesis of several inflammatory and autoimmune diseases.^9^ Systemic complement blockade—reducing dysregulated tissue complement via circulating complement inhibitors—has consequently garnered significant attention as a therapy for diseases including paroxysmal nocturnal hemoglobinuria, atypical hemolytic uremic syndrome, cold agglutinin disease, C3 glomerulopathy (C3G), IgA nephropathy (IgAN), bullous pemphigoid (BP), and geographic atrophy.^10^^,^^11^^,^^12^^,^^13^^,^^14^ However, because of its essential role in innate immunity, systemic complement inhibition through long-half-life circulating inhibitors increases susceptibility to bacterial infections, including life-threatening meningococcal (*Neisseria meningitidis*) infection and sepsis, even in vaccinated patients.^15^^,^^16^^,^^17^ One approach to mitigating this risk has been to selectively inhibit downstream terminal complement activation, for example by blocking C5 cleavage, thereby leaving proximal complement intact.^18^ However, recent data in membranous nephropathy (MN) indicate that C3 activation and AP amplification play a major role in disease by upregulating C3aR1 and C5aR1 expression on podocytes, suggesting that inhibition of proximal complement may be more effective than distal blockade.^8^^,^^19^^,^^20^

Circulating complement inhibitors also face a second challenge: many complement proteins are highly abundant in circulation and undergo rapid turnover, creating a large pharmacologic sink. Thus, efficacy of many systemically acting complement therapeutics requires high circulating concentrations, and patients can experience suboptimal disease control when these concentrations are not achieved or maintained.^21^^,^^22^^,^^23^ Furthermore, these drugs often must be delivered in large volumes and high doses, requiring biweekly or monthly infusions by a trained healthcare provider, adding to patient burden, treatment cost, and risks of short- and long-term toxicity.^24^^,^^25^^,^^26^^,^^27^^,^^28^ These factors illustrate the difficult balance between drug exposures high enough to effectively inhibit complement but low enough to minimize patients’ infection risk, and substantial unmet need remains for safer and more effective anti-complement therapies.

Many complement-mediated diseases are characterized by highly localized complement activation.^29^^,^^30^ Concentrating an inhibitor at sites of pathogenic active complement could therefore address the challenges of circulating complement blockade. Such a tissue-targeted drug could avoid pharmacologic sinks, thereby improving potency. Furthermore, this approach may enable durable complement control in tissues after the circulating drug has cleared, leaving the complement system largely intact in unaffected tissues and in circulation, thereby potentially improving safety.

To test the feasibility of this strategy, we explored use of targeted human and rodent bifunctional fusion proteins to locally deliver an active fragment of factor H (fH), a critical negative regulator of AP complement that is predominantly synthesized in liver.^31^^,^^32^ In both in the fluid phase and on surfaces, fH controls the AP amplification loop by catalyzing dissociation of the AP convertases and irreversible proteolytic degradation of convertase components.^31^^,^^33^ Five N-terminal short consensus repeats (SCRs) of fH (fH_1–5_) are sufficient for both AP convertase dissociation and degradation.^34^^,^^35^ We show that fH_1–5_ localization to high-density surface-bound AP convertases can be achieved by fusion to a monoclonal antibody that recognizes a common epitope with the complement fragments iC3b, C3dg, and C3d, which are covalently deposited at high density on tissues where complement is active.^36^^,^^37^^,^^38^^,^^39^ For simplicity, we refer to this antibody as “anti-C3d” and the human and rodent fusion proteins collectively as C3d-mAb-2fH.

Together, the data presented here demonstrate three important attributes of this C3d-targeted strategy: First, tissue C3d deposition co-localizes with active complement in multiple autoimmune kidney and skin conditions, supporting a translational strategy that focuses on the presence of C3d in diseased tissue. Second, we demonstrate that high-affinity anti-C3d antibody binding can efficiently localize a complement regulator in multiple tissues and across species. Finally, in the passive Heymann nephritis (PHN) antibody-driven model of membranous nephropathy in rats, we show that local complement inhibition is sufficient to modulate disease progression *in vivo*. C3d-mAb-2fH fusion proteins in this model inhibited glomerular complement and progression of renal injury without affecting systemic complement. These studies therefore point to the therapeutic potential of a humanized C3d-mAb-2fH, ADX-097, suggesting C3d-directed targeting as a strategy for potent, localized complement inhibitors.

## Results

### C3d deposition in human dermal and renal diseases

**Figure 1:**
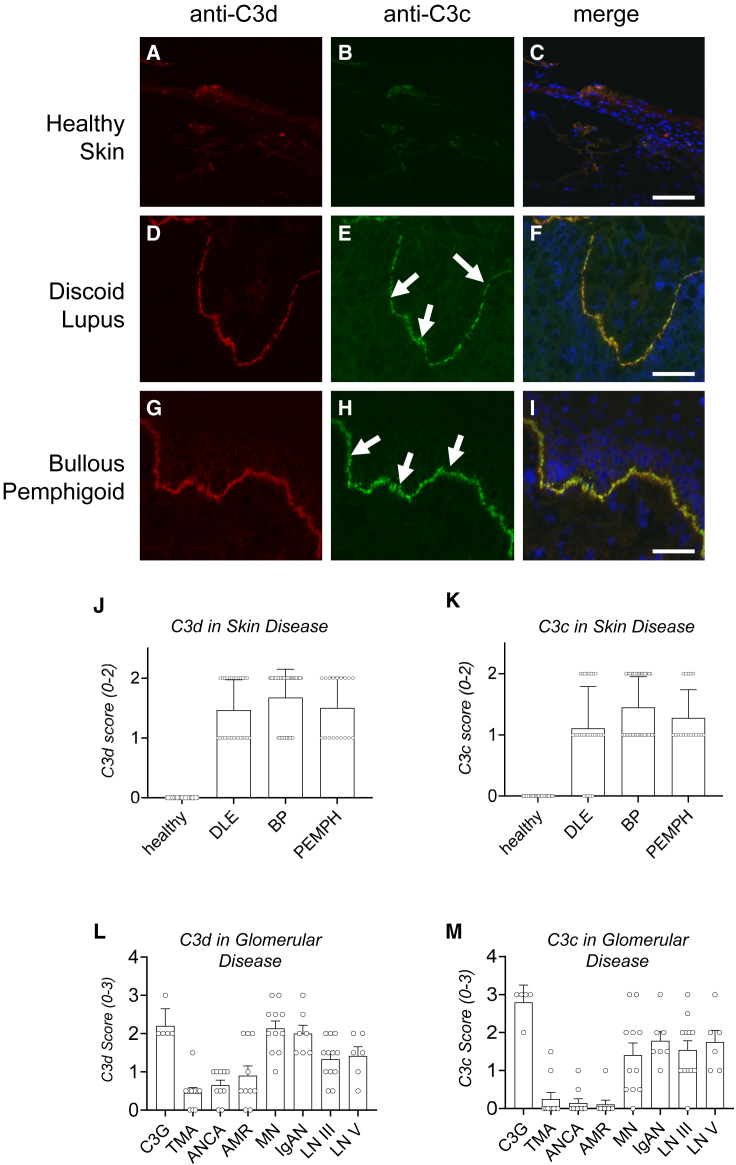
C3d target deposition in human disease Representative glomerular immunofluorescence in human biopsies from (A–C) healthy skin, (D–F) skin from a patient with discoid lupus erythematosus (DLE), and (G–I) skin from a bullous pemphigoid (BP) patient. Biopsies were stained with anti-C3d antibody (A, D, G) or anti-C3c antibody, which recognizes fragments generated by active complement (B, E, H). White arrows in (E) and (H) indicate the location of the dermal-epidermal junction (DEJ). Co-localization of anti-C3d (red) and C3c (green) immunostaining is shown in (C), (F), and (I), together with DAPI co-staining (blue). (J) and (K) patient show semiquantitative scoring of anti-C3d (J) and anti-C3c (K) in biopsies from healthy skin (n = 30) and from DLE (n = 28), BP (n = 40), and Pemphigus (PEMPH) (n = 18) patients. (L) and (M) show semiquantitative scoring of anti-C3d (L) and anti-C3c (M) in biopsies from patients with C3 glomerulopathy (C3G) (n = 5), thrombotic microangiopathy (TMA) (n = 10), anti-neutrophilic cytoplasmic autoantibody vasculitis (ANCA) (n = 10), antibody-mediated rejection of kidney transplant (AMR) (n = 10), membranous nephropathy (MN) (n = 11), IgA nephropathy (IgAN) (n = 7), and class III and class IV lupus nephritis (n = 12 and n = 6, respectively). Scale bar in (C), 100 μm. Scale bar in (F) and (I), 50 μm.

C3d represents an attractive localization target to bring fH to complement-active tissues due to its covalent link to cell surfaces and associated reported long tissue-residence time.^36^^,^^37^^,^^39^^,^^40^^,^^41^ However, while immunostaining using anti-C3c antibody is a common clinical diagnostic of active complement in kidney and skin, C3d deposition is less well characterized.^40^ To better understand the relationship between C3c and C3d deposition across indications, we surveyed tissue from a subset of skin and kidney diseases. Anti-C3d and anti-C3c immunofluorescence revealed minimal background staining in healthy human skin (Figures 1A–1C). In contrast, both C3c and C3d immunostaining were detected in a coincident pattern at the dermal-epidermal junction (DEJ) of affected skin in patients diagnosed with discoid lupus erythematosus (DLE) (Figures 1D–1F) or BP (Figures 1G–1I). Semiquantitative scoring of C3d- and C3c-immunostained samples from patients diagnosed with DLE, BP, and pemphigus revealed consistent deposition of both complement fragments across all three skin diseases (Figures 1J and 1K).

In kidney, strong C3d immunofluorescence was present in biopsies from patients with C3G (Figure S1A), consistent with the well-established role for complement in this disease.^42^^,^^43^^,^^44^ Low C3d immunostaining was detected in samples from patients diagnosed with thrombotic microangiopathy (Figure S1B), while moderate staining was present in samples from patients with anti-neutrophilic cytoplasmic autoantibody (ANCA) vasculitis (Figure S1C) and antibody-mediated rejection of transplanted kidney (Figure S1D). Strong immunostaining was evident in samples from MN (Figure S1E), IgAN (Figure S1F), and both class III and class IV lupus (Figures S1G and S1H). Semiquantitative scoring of anti-C3d immunostaining confirmed generally higher-density C3d deposition in C3G, MN, IgAN, and both classes of lupus nephritis (Figure 1L). These findings are also broadly consistent with similar quantitation of anti-C3 fragment (C3c) (Figure 1M).

To further explore the prevalence of complement activity in selected renal diseases, we performed a retrospective analysis of C3 fragment staining in a larger sample cohort (Table S1). Consistent with our prospective analysis, these data revealed a significant number of ANCA patients (43/104) that were C3c fragment positive, while patients with MN (86.2% of samples positive), IgAN (89.7% of samples positive), and lupus nephritis (96.3% of class III and 87.0% of class IV samples positive) indicated a high prevalence of glomerular complement activation in these diseases. Taken together, these data in skin and kidney suggest that C3d deposition is a feature of a subset of autoimmune diseases affecting multiple organs, and that C3d targeting could be a means of locally delivering a complement inhibitor for these indications.

### Generation of human and mouse anti-C3d-fH_1–5_ fusion proteins and characterization of binding to C3d

**Figure 2:**
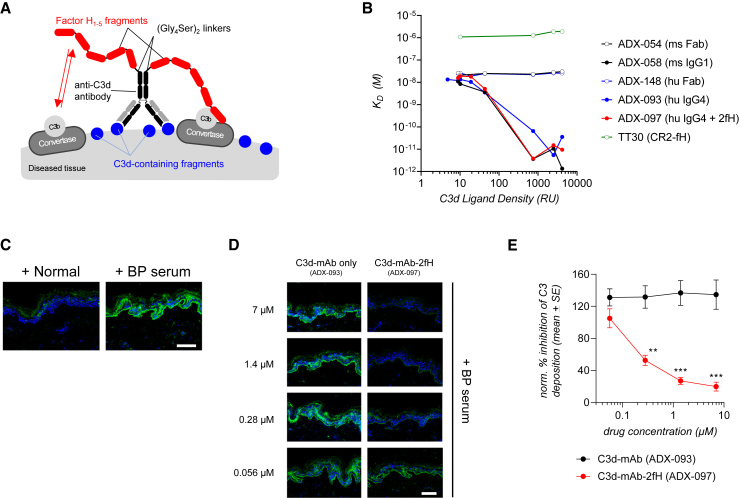
C3d-mAb-2fH design, comparison of binding affinities, and inhibition of complement deposition on human skin (A) Schematic of the design of C3d-mAb-2fH. (B) Summary of binding affinities measured by surface plasmon resonance (SPR) with increasing density of recombinant C3d. Binding affinity of mouse (ms) (ADX-054) and human (hu) (ADX-148) C3d-binding Fab proteins is roughly 75-fold greater than TT30/CR2-fH at all C3d densities tested. Binding of mouse (ADX-058) and human (ADX093) C3d-targeting monoclonal antibodies (mAbs), or of the human C3d-mAb-2fH fusion protein ADX-097, is similar to anti-C3d Fab binding at low density but increases by >100-fold at higher C3d densities. (C) Deposition of complement fragments (green) after incubation of sections of human skin with serum from bullous pemphigoid (BP) patients, but not after incubation with serum from non-diseased patients. Scale bar, 100 μm. (D) Addition of human C3d-mAb-2fH (ADX-097) fusion protein, but not of the C3d-targeting antibody alone (ADX-093), inhibits deposition on sections of human skin that has been exposed to BP serum. Scale bar, 100 μm. (E) Dose-response curve of the effect of ADX-097 and ADX-093 treatment on complement deposition on human skin sections. ADX-097 significantly inhibits complement deposition in the nanomolar range (∗∗p < 0.0002; ∗∗∗p < 0.00002).

We generated anti-C3d-targeted fH_1–5_ fusion proteins, collectively designated C3d-mAb-2fH, to localize complement inhibition to C3d-positive tissue. Mouse and human C3d-mAb-2fH are approximately 213 kDa recombinant bifunctional fusion proteins consisting of an anti-C3d monoclonal antibody linked to two moieties of the first five SCRs of factor H (fH_1–5_) (Figure 2A). Mouse C3d-mAb-2fH (ADX-118) and the mouse/human chimeric fusion ADX-048 rely on a previously identified monoclonal mouse anti-C3d IgG1 antibody (3d8b) that binds with low-nanomolar affinity to an epitope present in mouse, cynomolgus monkey, and human C3d, iC3b, and C3dg (Table S2).^45^^,^^46^ As noted earlier, for simplicity we refer to this binding target as “anti-C3d.” The 3d8b antibody was subsequently humanized by CDR (complementarity-determining regions) grafting onto a human germline acceptor framework, followed by additional amino acid modifications to reduce potential immunogenicity, improve antibody stability, and minimize potential for antibody-dependent cell-mediated cytotoxicity or complement-dependent cytotoxicity.^46^ The resulting human IgG4 antibody, ADX-093, retained similar affinity as 3d8b to human C3d (Table S2) and was therefore used in the human C3d-mAb-2fH fusion protein ADX-097.

The first five SCRs of fH (fH_1–5_) are both necessary and sufficient to catalyze AP convertase dissociation by accelerating decay of the C3bBb complex and by serving as a co-factor for factor I-mediated cleavage of C3b to iC3b.^34^^,^^35^ Prior work from our group demonstrated that fusion of CR1 complement regulatory domains to the C termini of targeting antibody heavy chains yielded optimal complement inhibition.^46^ Similarly, fH_1–5_ moieties to the antibody heavy-chain C termini via (Gly_4_Ser)_2_ linkers (Figure 2A) did not affect anti-C3d binding, as ADX-118 (mouse antibody/mouse fH_1–5_) and ADX-097 (human antibody/human fH_1–5_) bound mouse, cynomolgus monkey, and human C3d with similar affinity as their parent antibodies, 3d8b and ADX-093 (Table S2).

ADX-097 and targeting antibody alone (ADX-093, ADX-058) showed similar binding affinities by surface plasmon resonance (SPR) measured over a range of C3d densities (Figure 2B and Table 2), further indicating that the fH_1–5_ moieties on ADX-097 do not affect C3d binding. Increasing C3d density by 420-fold led to an approximately 2,000-fold increase in binding affinity (*K*_D_) compared to monovalent Fab fragments ADX-148 and ADX-054 (Figure 2B and Table 2). The lower dissociation constants (*k*_off_) of the bivalent monoclonal antibodies (mAbs) indicate that this is likely driven by avidity (Table 2). We also compared binding of C3d-mAb-2fH to TT30, which monovalently localizes a single moiety of fH to C3d and related fragments via a domain of the B cell C3d/iC3b receptor CR2 (CD21).^35^^,^^47^^,^^48^^,^^49^ At all C3d densities tested, the *K*_D_ of TT30 was in the low-micromolar range, with a rapid off-rate that made it impossible to calculate an accurate *k*_off_ (Figure 2B and Table 2). This is 50- to 75-fold weaker binding affinity than anti-C3d Fabs at any C3d density and >150,000-fold weaker affinity than ADX-097 at high C3d density. Taken together, these binding data illustrate two important advantages of localizing fH_1–5_ using a bivalent antibody. First, antibodies are capable of high-affinity monovalent anti-C3d binding that is not achieved using fragments of an endogenous C3d-binding protein. Second, a bivalent antibody biases fH_1–5_ targeting toward high-density C3d deposition, potentially favoring localization to dysregulated rather than to homeostatic complement activity.

**Table 2:** C3d-mAb-2fH binding affinity rises with increased C3d density

**Protein ID**	**Anti-C3d antibody**	**Low C3d density (10 RU)**		**Low-med C3d density (40 RU)**		**Med C3d density (750 RU)**		**Med-high C3d density (2,500** RU)		**High C3d density (4,200 RU)**	
		***K***_**D**_**(nM)**	***k***_**off**_**(1/s)**	***K***_**D**_**(nM)**	***k***_**off**_**(1/s)**	***K***_**D**_**(nM)**	***k***_**off**_**(1/s)**	***K***_**D**_**(nM)**	***k***_**off**_**(1/s)**	***K***_**D**_**(nM)**	***k***_**off**_**(1/s)**
ADX-058 (3d8b)	ms IgG1	8.47	9.7 × 10^−4^	3.67	3.9 × 10^−4^	0.004	4.1 × 10^−7^	0.011	1.2 × 10^−6^	0.001	1.5 × 10^−7^
ADX-054	ms Fab	20.9	1.8 × 10^−3^	24.9	3.9 × 10^−4^	23.0	1.6 × 10^−3^	27.9	1.6 × 10^−3^	30.3	1.4 × 10^−3^
ADX-093	hu IgG4	13.4	7.8 × 10^−4^	3.49	3.9 × 10^−4^	0.065	4.9 × 10^−6^	0.005	4.4 × 10^−7^	0.036	2.9 × 10^−6^
ADX-148	hu Fab	26.1	1.4 × 10^−3^	24.5	3.9 × 10^−4^	21.9	1.5 × 10^−3^	25.7	1.4 × 10^−3^	25.8	1.3 × 10^−3^
ADX-097	hu IgG4 + 2fH	18.8	8.2 × 10^−4^	5.06	3.9 × 10^−4^	0.004	2.3 × 10^−7^	0.015	9.8 × 10^−7^	0.010	6.7 × 10^−7^
TT30	hu CR2-fH	1,080	ND	NT	NT	1,260	ND	1,890	ND	1,890	ND
**Fold ↑ C3d density**		**1×**		**4×**		**75×**		**250×**		**420×**	

RU, relative density units; ms, mouse; hu, human; fH, factor H_1–5_ fragment; ND, not able to determine; NT, not tested.

### *In vitro* characterization of complement inhibition by human and mouse C3d-targeted fH_1–5_

To understand whether linking fH_1–5_ to an anti-C3d antibody affects its C3 regulatory properties, we assessed the ability of C3d-mAb-2fH to act as a factor I (fI) co-factor, catalyzing fluid-phase C3b cleavage.^50^^,^^51^^,^^52^^,^^53^ C3b contains two distinct chains, α′ (110 kDa) and β (70 kDa), that dissociate under reducing conditions. Together, fI and fH cleave the α′ chain into smaller protein fragments that can be separated and visualized by SDS-PAGE. Recombinant C3b co-incubated with fI in the presence of anti-C3d antibody (ADX-093) did not cleave C3b (Figure S2A). Combining C3b, fI, and full-length fH (FL fH) resulted in dose-dependent cleavage of the 110-kDa C3α′ chain into 68-, 46-, and 43-kDa fragments (C3α′ −68, −46, and −43, Figure S2B), demonstrating co-factor activity of fH. fH_1–5_ resulted in similar C3b cleavage (Figure S2C), although its catalytic potency was somewhat lower than that of FL fH (Figure S2E). ADX-097 (human C3d-mAb-2fH) also catalyzed fI-mediated C3b cleavage (Figure S2D). The cleavage efficiency of ADX-097 was similar to that of fH_1–5_ (Figures S2E and S2F), indicating that in this purely fluid-phase assay, linking to fH_1–5_ to an antibody did not substantially affect its fI co-factor activity.

To measure complement inhibition more quantitatively, we evaluated the effect of C3d-mAb-2fH fusions on formation of AP- or CP-generated C5b-9 (MAC) in human complement-preserved serum (Wieslab assays). Human fH_1–5_ inhibited AP complement (half-maximal inhibitory concentration [IC_50_] = 1,980 ± 163 nM) but demonstrated no measurable inhibition of CP complement (Table 1). Fusion of two fH_1–5_ moieties to a human Fc domain (ADX-145) demonstrated a roughly 6-fold increase in AP complement inhibition (IC_50_ = 325 ± 61 nM), suggesting that the presence of a second fH_1–5_ domain may confer an avidity effect. The humanized anti-C3d antibody ADX-093 by itself showed no inhibition of AP complement, but fusion of two fH_1–5_ domains (ADX-097) resulted in a further 4-fold increase in potency vs. ADX-145 (IC_50_ = 81 ± 3.9 nM). ADX-097 retained strong selectivity for AP complement, as inhibition of CP-initiated complement activity remained approximately 10-fold weaker (IC_50_ = 610 ± 83 nM). A chimeric molecule consisting of two moieties of human fH_1–5_ linked to mouse 3d8b (ADX-048) showed AP complement inhibition similar to that of ADX-097 (IC_50_ = 73 ± 8.3 nM), indicating that fusion of fH_1–5_ to either the mouse or human anti-C3d antibodies results in similar potency.

**Table 1:** *In vitro* complement inhibition by C3d-mAb-fH fusion proteins

**Protein ID**	**Anti-C3d antibody**	**Backbone**	**Effector**	**Complement pathway (Wieslab assay)**	**IC**_**50**_**(nM)**
sfH_1–5_	–	–	1× human fH_1–5_	alternative	1,980 ± 163
				classical	no activity
ADX-048	3d8b	mouse IgG1	2× human fH_1–5_	alternative	73 ± 8.3
ADX-093	humanized 3d8b	human IgG4	–	alternative	no activity
ADX-145	–	Fc fusion (IgG4)	2× human fH_1–5_	alternative	325 ± 61
ADX-097	humanized 3d8b	human IgG4	2× human fH_1–5_	alternative	81 ± 3.9
				classical	610 ± 93

Wieslab AP and CP assays are designed for human serum and do not work well in rodent sera.^54^ Therefore, to assess C3d-mAb-2fH potency in mouse and rat, a crucial prerequisite for *in vivo* testing in rodent species, we used an assay that relies on zymosan particles incubated in complement-preserved serum. The human fusion protein ADX-097 showed similar potency in human and mouse serum (IC_50_ = 191 ± 17 nM in human and 202 ± 54 nM in mouse) and was slightly more potent in rat serum (IC_50_ = 99 ± 17 nM) (Table S3). In comparison to ADX-097, the mouse fusion protein ADX-118 was 3- to 4-fold more potent in mouse (IC_50_ = 46 ± 3.9) but 2- to 3-fold less potent in rat serum (281 ± 52 nM). ADX-118 showed no complement inhibition in human serum. Together, these data demonstrate activity of both the mouse and human fusion proteins in rodent, enabling subsequent *in vivo* studies to assess tissue-targeted pharmacokinetics and pharmacodynamics (PK/PD).

### Evaluation of C3d-mAb-2fH activity on human skin explants

To test inhibition of complement in the context of human tissue, we evaluated human C3d-mAb-2fH (ADX-097) in a skin explant assay.^55^ Cryosections of human skin were pre-incubated with heat-inactivated (complement-inactive) normal human serum or serum from BP patients, allowing pathogenic autoantibodies in the BP serum to bind the skin section. After washing, sections were then incubated with complement-active human serum (with or without inhibitors), and complement deposition was detected by immunofluorescence with an anti-C3c antibody. Sections pre-incubated with normal serum exhibit minimal anti-C3c immunofluorescence, while those pre-incubated with BP serum show substantial C3c signal, indicating C3b tissue deposition (Figure 2C). Addition of the anti-C3d-binding antibody ADX-093 had no effect, while ADX-097 concentrations as low as 0.28 μM significantly inhibited skin C3b deposition (p < 0.0002, Figures 2D and 2E), with complete inhibition of complement occurring between 0.28 and 1.4 μM (60–300 μg/mL ADX-097 in serum). These data indicate that ADX-097 can inhibit human tissue complement deposition, even in the highly dysregulated context of BP serum.

### C3d-targeted fH_1–5_ localized to and blocked local tissue complement in *CfH*^*−/−*^ mice

We evaluated the C3d-mAb-2fH *in vivo* distribution and local complement inhibition in fH knockout mice (*CfH*^*−/−*^), which have uncontrolled systemic complement activation and increased C3 deposition in the liver and kidney, resulting in sporadic complement-mediated kidney injury.^56^ Tissue complement activity in kidney and liver was evaluated by immunofluorescence with an antibody against non-tissue-linked active C3 split products (C3b/iC3b/C3c, which we collectively refer to as “anti-C3 fragment”) and for drug localization by staining with anti-human fH (anti-fH). After a single intravenous (i.v.) dose of untargeted human fH_1–5_ (hu fH_1–5_), anti-fH immunostaining indicated no localization to liver or kidney (Figures S3G and S3O), and complement activity in these tissues was unchanged compared to *CfH*^*−/−*^ + PBS (Figures S3B, S3C, S3J, and S3K). In contrast, significant anti-fH immunostaining was evident in *CfH*^*−/−*^ mice treated with chimeric C3d-mAb-2fH (ADX-048) (Figures S3H and S3P), demonstrating targeting to tissues with active complement. These mice also showed a marked decrease in both liver and kidney anti-C3 fragment immunofluorescence (compare Figures S3D and S3L to S3C and S3K), indicating that, unlike non-targeted hu fH_1–5_, C3d-mAb-2fH potently inhibits complement activity in tissue for at least a week after dosing.

We next assessed the kinetics of C3d-mAb-2fH-mediated glomerular complement inhibition using a 5 mg/kg i.v. dose of C3d-mAb-2fH, C3d-targeting antibody alone (ADX-093), or vehicle control. C3 fragment immunofluorescence, measured by digital image quantitation, was similar in ADX-093-dosed *CfH*^*−/−*^ mice and in untreated *CfH*^*−/−*^ mice (compare black line and shaded area in Figure S3Q). However, dosing with ADX-097 significantly and durably reduced glomerular C3 fragment deposition in *CfH*^*−/−*^ mice (red line in Figure S3Q). Anti-human IgG4 immunostaining demonstrated accumulation of both the ADX-093 and ADX-097 proteins to glomeruli (Figures S4E and S4F). Detection with both anti-fH and anti-IgG4 show similar tissue distribution (compare Figures S3H and S4F), indicating that the ADX-097 fusion protein remains intact *in vivo*. These data demonstrate that both the human and human/mouse chimeric C3d-mAb-2fH fusion proteins localize to and block tissue convertase activity in *CfH*^*−/−*^ mice and that inhibition requires the presence of fH moieties.

**Figure 3:**
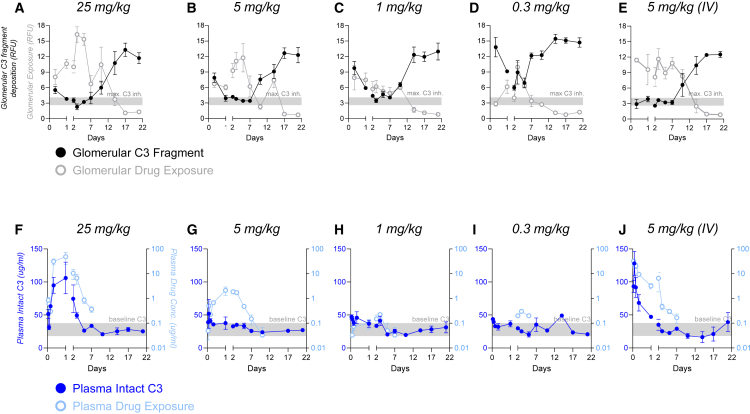
Glomerular and circulating pharmacokinetics and pharmacodynamics of C3d-mAb-2fH *CfH*^*−/−*^ mice received a single dose of mouse C3d-mAb-2fH (ADX-118) at the indicated concentrations. Dosing route was s.c. unless otherwise indicated. Kidney tissue was collected at 8 h and 1, 2, 3, 5, 7, 10, 14, 17, and 22 days after dosing (n = 4 mice per time point). Plasma was collected at 1, 2, 4, and 8 h, and 1, 2, 3, 5, 7, 10, 14, 17, and 22 days after dosing. (A–E) Summary of image quantitation data for C3 fragment deposition (black lines) and tissue drug levels (anti-fH, gray lines). ADX-118 levels were dose dependent (compare gray lines in A–D) and showed measurable drug levels in kidney for at least 2 weeks after dosing. Tissue *C*_max_ of 5 mg/kg s.c. (B) and i.v. (E) were similar. The level of maximum C3 fragment inhibition was defined as the average C3 fragment level in the 25 mg/kg group at 1, 2, 3, and 5 days ± 1 SD (A) and is indicated as a gray bar in (A)–(E). Peak inhibition of C3 fragment deposition was similar in the 25 mg/kg (A), 5 mg/kg (B, E), and 1 mg/kg (C) groups, while partial inhibition is achieved in the 0.3 mg/kg group (D). Measurement of circulating drug exposure (light-blue lines) and circulating intact C3 (dark-blue lines) is shown in (F)–(J). Circulating ADX-118 levels were dose dependent (compare light-blue lines in F–I). Circulating *C*_max_ was approximately 15-fold higher for i.v. vs. s.c. delivery (5 mg/kg) (compare G and J). Circulating intact C3 levels transiently increased in the 25 mg/kg s.c. (F) and 5 mg/kg i.v. (J) groups, as these groups achieved sufficient circulating drug concentration to transiently slow C3 consumption. However, despite inhibiting complement in glomeruli, s.c. doses ≤5 mg/kg did not attain sufficiently high circulating C3d-mAb-2fH concentrations to affect systemic C3 cleavage (G–I).

A detailed dose-ranging time course in *CfH*^*−/−*^ mice more thoroughly evaluated the relationship between circulating and tissue PK/PD. These studies used mouse C3d-mAb-2fH, ADX-118, to minimize the potential for cross-species anti-drug antibody (ADA) formation that could affect drug exposure. ADX-118 was delivered subcutaneously (s.c.) at doses ranging from 0.3 to 25 mg/kg and i.v. at 5 mg/kg. All doses resulted in glomerular localization of ADX-118 (gray lines in Figures 3A–3E; representative immunofluorescence is shown in Figure S5), with maximal drug concentration in tissue (tissue *C*_max_) correlating to drug dose. At all doses, localized drug was detected in tissue for at least 10 days, returning to background levels by 14–17 days post dose. Comparison of 5 mg/kg delivered s.c. vs. i.v. suggests that the i.v. route distributed more rapidly to tissue, translating to greater tissue complement inhibition at the 8-h time point (compare black lines in Figures 3B and 3E; representative immunofluorescence is shown in Figure S6). However, both routes of administration achieved similar tissue *C*_max_ and, beyond the first 24 h after dosing, both inhibited complement similarly. While tissue drug exposure was dose correlated, 1, 5, or 25 mg/kg s.c. ADX-118 doses led to similar maximal complement inhibition, although lower doses required more time to reach maximal inhibition (black lines in Figures 3A–3C). Importantly, maximum tissue complement inhibition in the 1 mg/kg and 5 mg/kg s.c. groups was reached prior to tissue *C*_max_ (Figures 3B, 3C, and S6), suggesting that anti-C3d target saturation is not required to fully inhibit tissue AP complement. The 0.3 mg/kg dose group inhibited complement for several days but did not reach the same maximum achieved by the 1, 5, and 25 mg/kg doses (Figure 3D). Finally, 1 week after dosing, the 1, 5, and 25 mg/kg groups all retained maximal complement inhibition, indicating that ADX-118-mediated tissue complement inhibition is quite durable.

### Local complement can be inhibited by C3d-mAb-2fH at doses that do not affect circulating complement

Because uncontrolled AP complement activity in *CfH*^*−/−*^ mice consumes C3 faster than new C3 protein is generated, these mice exhibit a 10- to 20-fold reduction in circulating intact C3 protein levels.^56^ *In vivo* administration of an exogenous AP complement inhibitor temporarily reduces this consumption, leading to a transient increase in intact plasma C3 that can serve as a sensitive biomarker of systemic complement inhibition.^57^^,^^58^^,^^59^ Twenty-four hours after delivery, a single 25 mg/kg s.c. dose of ADX-118 reached a circulating *C*_max_ of approximately 50 μg/mL (light blue in Figure 3F), corresponding to a measurable increase in intact plasma C3 that returned to baseline as drug cleared from circulation (dark blue in Figure 3F). I.v. delivery of 5 mg/kg ADX-118 also resulted in sufficient drug exposure (∼30 μg/mL) to elicit transient elevation of intact plasma C3 (Figure 3J). In contrast, s.c. delivery of ≤5 mg/kg ADX-118 led to lower *C*_max_ and overall exposure levels, resulting in negligible plasma C3 elevation (Figures 3G–3I). Thus, while s.c. doses of 1–5 mg/kg are capable of potent and durable local inhibition in tissue (Figures 3B and 3C), they do not achieve sufficiently high plasma drug concentrations to affect circulating complement in *CfH*^*−/−*^ mice.

### C3d-mAb-2fH localized to active complement in primate skin

We next examined whether human C3d-mAb-2fH (ADX-097) can target local complement in primates. High-dose UVB irradiation activates epidermal complement,^60^^,^^61^ and we therefore developed a model of UVB-induced skin complement activation in cynomolgus monkeys. Erythema was induced in monkey skin by transient exposure to UVB light using a hand-held lamp (Figures S7A–S7D). A time course of skin biopsies was collected after exposure and immunostained using anti-C3c and anti-C3d antibodies (Figures S7E–S7H and S7I–S7L). Co-localized deposition of complement fragments was observed in the epidermis as early as 24 h after exposure and lasted for at least 72 h (Figures S7M–S7P).

**Figure 4:**
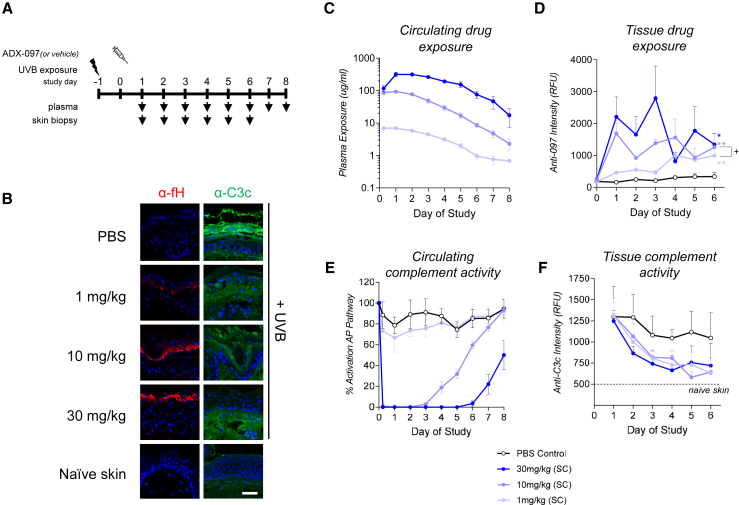
C3d-mAb-2fH localizes to skin in cynomolgus monkeys (A) Study design to test distribution and complement inhibition in UVB-induced cynomolgus monkey skin. Discrete sections of shaved skin were exposed to 3,900 mJ/cm^2^ UVB irradiation on day −1 of the study. ADX-097 or PBS vehicle control was delivered systemically by s.c. injection. Skin biopsies from the irradiated areas and plasma were collected daily for 6 days after dosing. (B) Representative images from sections collected 72 h after ADX-097 dosing. Sections were stained with an anti-fH antibody (red) to detect localization of fH_1–5_ in skin or with an antibody that recognizes C3c fragments (green) to detect areas of active complement. All sections were counterstained with DAPI (blue). Scale bar, 50 μm. (C) Plasma exposure (μg/mL) of ADX-097, measured by ELISA, over time. (D) Tissue exposure of ADX-097, measured by anti-fH immunofluorescence and digitally quantified, over time demonstrate drug localization to complement-active epidermis. Area under the curve (AUC) of anti-fH immunostaining for all time points is statistically different vs. PBS control for all ADX-097 dosed groups (∗p < 0.02, ∗∗p < 0.005). AUC of the 10 mg/kg ADX-097 group is greater than that of the 1 mg/kg dosed monkeys (^+^p < 0.05). Tissue exposure (AUC) of the 30 mg/kg group is not statistically different from the 10 mg/kg group (p = 0.26) but is statistically different from the 1 mg/kg group (p < 0.04). (E) Systemic complement inhibition was measured by Wieslab assay in monkey serum. Thirty mg/kg and 10 mg/kg ADX-097 inhibited complement in serum in a dose-dependent manner (p < 0.0001, %Activity_AUC_ between the two doses). A non-statistically significant trend toward inhibition was observed between the %Activity_AUC_ of the PBS control and 1 mg/kg ADX-097 groups (p = 0.07), suggesting the possibility of minimal systemic inhibition at early time points after ADX-097 dosing. (F) Tissue complement inhibition was measured by anti-C3c immunostaining and image quantitation. All doses of ADX-097 tested resulted in a non-statistically significant trend (p = 0.11–0.16) toward reduced tissue complement compared to PBS-treated controls.

To evaluate localization of C3d-mAb-2fH to primate skin, UVB injury was induced on study day −1, 24 h prior to drug dosing (Figure 4A). Complement activation, marked by anti-C3c immunostaining, was observed after UVB exposure in vehicle (PBS)-treated monkeys (compare PBS to naive skin, Figures 4B and S8). Human C3d-mAb-2fH was administered systemically by s.c. injection on day 0, and drug localization was detected as early as 24 h after dosing (Figure S8). Total tissue drug exposure, measured as area under the curve (AUC), was dose correlated and was greater than the PBS control for all doses tested (p < 0.02) (Figures 4B–4D and S8). Notably, while ADX-097 localizes to tissue in the 1 mg/kg dose group, no inhibition of systemic complement was observed in plasma collected from this group (Figure 4E). In the 10 mg/kg and 30 mg/kg dose groups, inhibition of circulating complement waned as circulating drug levels dropped below approximately 70 μg/mL (Figures 4C and 4E). Circulating drug concentration in the 1 mg/kg group remained below 10 μg/mL throughout the study (Figure 4C). All tested doses of ADX-097 showed similar tissue complement inhibition, reaching maximal inhibition roughly 3 days after s.c. dosing (Figure 4F). However, this difference was not statistically significant (p = 0.11–0.15), a likely consequence of the small number of samples (n = 3) collected at each time point. Nevertheless, these data are consistent with those observed in *CfH*^*−/−*^ mice, suggesting that in addition to homing to skin complement, ADX-097 may locally regulate complement in non-human primate (NHP) skin.

### Intravenous C3d-targeted fH_1–5_ reduced renal injury in a rat model of membranous nephropathy

We tested disease-modifying efficacy of C3d-mAb-2fH in the PHN model of membranous nephropathy in rats. PHN is induced by administering a sheep serum raised against a preparation of rat proximal tubules (anti-Fx1A).^62^^,^^63^ Anti-Fx1A causes subepithelial immune deposits in the glomerular basement membrane (GBM), leading to pathologic changes in the GBM and podocytes that are reflected as elevated urine protein levels.^62^ Initial renal injury in PHN is driven by complement activation, as treatment with cobra venom factor (CVF), a C3 analog that depletes endogenous complement, or with a small-molecule factor B inhibitor, effectively reduces disease in this model.^64^^,^^65^^,^^66^^,^^67^

**Figure 5:**
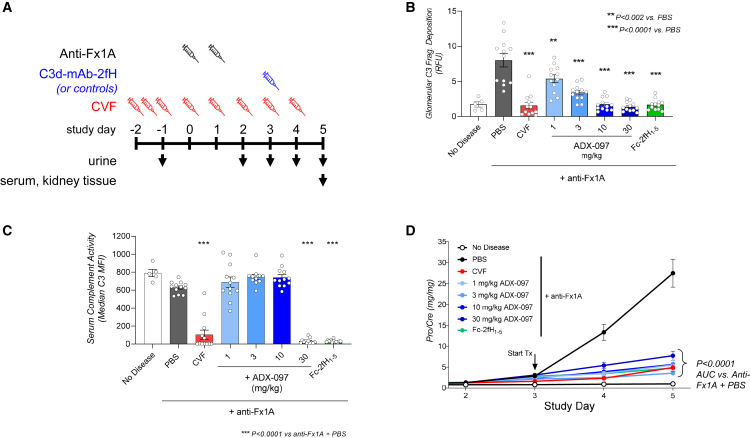
C3d-mAb-2fH reduces proteinuria and inhibits glomerular complement without affecting systemic complement (A) Study design in the passive Heymann nephritis (PHN) model of kidney injury. (B) Glomerular C3 fragment deposition was quantified from anti-C3 fragment immunofluorescence on kidney tissue. Compared to non-disease controls, C3 fragment deposition is elevated in PHN rats treated with PBS (p < 0.0001). CVF treatment reduces anti-C3 fragment to levels equivalent to non-disease controls (p < 0.0001). ADX-097 treatment reduces glomerular C3 fragment deposition in a dose-dependent manner: 1 mg/kg ADX-097 reduces anti-C3 fragment immunofluorescence by approximately 40% (p < 0.002), and 3 mg/kg reduces it by approximately 70% (p < 0.0001). ADX-097 doses ≥10 mg/kg, as well as 17 mg/kg Fc-2fH_1–5_, reduced anti-C3 fragment immunofluorescence to levels equivalent to non-diseased controls. (C) Compared to healthy controls, serum complement activity was slightly but not statistically significantly reduced in PHN rats treated with PBS. Activity was significantly inhibited in PHN rats treated with CVF, 50 mg/kg ADX-097, or 17 mg/kg Fc-2fH_1–5_ (all p < 0.0001 vs. PHN + PBS). In contrast, compared to PHN + PBS, ADX-097 doses ≤10 mg/kg did not affect circulating complement. (D) Urine protein/creatinine ratio (uPCR) was elevated by day 3 in rats treated with anti-Fx1A nephrotoxic serum alone and continued to increase until the end of the study. Treatment with cobra venom factor (CVF), dosed daily beginning 2 days prior to anti-Fx1A instillation, significantly inhibited uPCR (p < 0.0001). Single i.v. doses of 1–30 mg/kg human C3d-mAb-2fH (ADX-097) inhibited uPCR progression (p < 0.0001). No dose response was observed, suggesting that 1 mg/kg ADX-097 achieves maximal efficacy in this time frame. A 17 mg/kg dose (equimolar to 30 mg/kg ADX-097) of a non-targeted inhibitor, Fc-2fH_1–5_, also inhibited uPCR progression.

Because C3d-mAb-2fH targeting occurs through C3d binding, we first assessed the time course of C3d deposition after anti-Fx1A treatment (Figure S9). Consistent with the role of complement in PHN, glomerular anti-C3d immunostaining was first detected in kidneys 2 days after delivery of anti-Fx1A (Figures S9A and S9B) while proteinuria, measured as urine protein/creatinine ratio (uPCR), appeared by day 3 (p < 0.001 vs. healthy control, Figure 5D). Based on these data, C3d-mAb-2fH was dosed on day 3 to evaluate therapeutic efficacy after disease onset.

We initially tested the human C3d-mAb-2fH, ADX-097, administered i.v. after onset of proteinuria (day 3 after disease induction) at doses ranging from 1 to 30 mg/kg. As a comparator, a separate cohort of rats was dosed daily with 100 U/kg CVF beginning 2 days prior to disease induction (for study design see Figure 5A). We used the human drug candidate ADX-097 in these studies because the short drug-treatment period (2–4 days) minimizes concerns that cross-species ADAs could affect drug exposure. Glomerular and systemic complement activity was assessed in samples collected 2 days after ADX-097 administration (day 5 after disease induction). Representative images from both anti-C3 fragment and anti-fH immunofluorescence are shown in Figure S10. In PHN kidney tissue, ADX-097 treatment led to dose-dependent reduction of glomerular complement activity (image quantitation of anti-C3 fragment immunostaining) (Figure 5B). Notably, while ADX-097 doses ≥10 mg/kg completely inhibited activity, the 1 mg/kg and 3 mg/kg dose groups showed approximately 40% and 75% reduction in C3 fragment deposition, respectively. Non-targeted Fc-2fH_1–5_ at a molar-equivalent dose to 30 mg/kg ADX-097 also inhibited glomerular C3 fragment deposition (Figure 5B). However, while both the 30 mg/kg ADX-097 and Fc-2fH_1–5_ groups had similar concentrations in circulation (Figure S11A), only ADX-097 localizes to glomeruli (Figure S11B), suggesting that Fc-fH_1–5_ acts through fluid-phase/circulating rather than local complement inhibition. Consistent with this hypothesis, both Fc-2fH_1–5_ and 30 mg/kg ADX-097 also inhibited serum complement (Figure 5C). In contrast, no systemic complement inhibition was detected in samples from animals treated with 1, 3, or 10 mg/kg ADX-097 (Figure 5C), indicating that C3d targeting drives the potency of ADX-097’s effects on complement-mediated disease at these lower doses.

All doses of ADX-097 reduced the progression of proteinuria (uPCR) as early as 24 h after injection and to a similar degree as prophylactic CVF treatment (Figure 5B). This was not due to diminished anti-Fx1A localization, as equivalent sheep IgG deposition was detected in ADX-097-treated and control PHN glomeruli collected 5 days after disease induction (2 days after ADX-097 dosing) (Figure S12). No correlation between ADX-097 dose and reduced uPCR progression was observed, suggesting that the minimal efficacious dose of ADX-097 is below the lowest tested dose (1 mg/kg). We note that the 1 mg/kg and 3 mg/kg doses yielded 40% and 75% decreases in glomerular anti-C3 fragment immunofluorescence, respectively, but their effect on proteinuria was comparable to fully blocking anti-C3 fragment immunostaining (Figures 5B and 5D). These data suggest that partial complement inhibition may be sufficient for disease-modifying efficacy in this model.

### Soluble C5b-9 in urine correlated with tissue complement inhibition

The C5b-9 protein complex is an end product of complement activation, leading to formation of pores that disrupt pathogen and target cell membranes, driving cell lysis and death.^68^ Tissue C5b-9 deposition is detected in a wide spectrum of kidney diseases, including membranous, IgA, hypertensive, and diabetic nephropathies, as well as lupus nephritis, thrombotic microangiopathies, and C3 glomerulopathy.^69^ Soluble C5b-9 is also detected in urine (uC5b-9) from PHN rats and from patients suffering from IgAN, membranous nephropathy, and pre-eclampsia, suggesting that uC5b-9 may also reflect tissue complement activity in the kidney.^70^^,^^71^^,^^72^^,^^73^ Because ADX-097 inhibits in tissue without affecting circulating complement, our PHN studies provide an opportunity to further evaluate uC5b-9 as an indicator of renal complement. In urine samples collected from PHN rats on study day 5, 48 h after treatment with PBS or with 1, 3, or 10 mg/kg ADX-097 (see Figure 5A), uC5b-9 concentration normalized to urine creatinine (uC5b-9/uCre) was reduced by ADX-097 in a dose-dependent manner (Figure S13A). Notably, all ADX-097-treated rats showed similar reductions in proteinuria (Figure 5D), suggesting that changes in uC5b-9/uCre do not simply reflect decreased urine protein. Furthermore, we found a highly significant correlation (Spearman’s r = 0.76; p < 0.00000001) between uC5b-9/uCre and tissue glomerular C3 fragment deposition, measured by anti-C3 fragment immunostaining (Figure S13B), suggesting that uC5b-9/uCre is a urine biomarker of kidney tissue complement activation.

### Low-dose, subcutaneous C3d-mAb-2fH reduced renal injury in PHN rats

**Figure 6:**
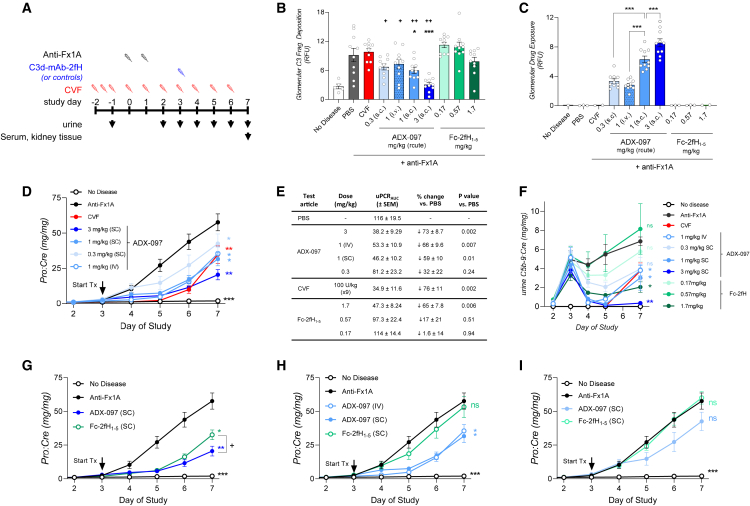
C3d targeting of fH dose-dependently reduces renal injury in passive Heymann nephritis (A) Summary of study design in the passive Heymann nephritis (PHN) model of kidney injury. PHN rats were treated with CVF starting on study day −2 (prior to disease induction by anti-Fx1A) or after onset of proteinuria on study day 3 with human C3d-mAb-2fH (ADX-097) at doses of 0.3, 1, or 3 mg/kg s.c. or with 1 mg/kg i.v. Fc-2fH_1–5_ was included in the study at s.c. doses equimolar to ADX-097. (B) Glomerular C3 fragment deposition (quantitation of anti-C3 fragment immunofluorescence) in glomeruli from PHN rats. C3 fragment deposition is significantly increased in anti-Fx1A-induced PHN kidneys (p < 0.0001). CVF-treated PHN rats do not exhibit reduced anti-C3 fragment signal, a likely consequence of CVF clearance due to immunogenicity. ADX-097-treated PHN rats show a dose-dependent inhibition of glomerular C3 fragment deposition, which is reduced to levels equivalent to non-diseased controls in the 3 mg/kg s.c. group and is partially reduced in the 1 mg/kg s.c. group (∗p < 0.05, ∗∗∗p < 0.0001 vs. anti-Fx1A + PBS). None of the Fc-2fH_1–5_-treated dose groups show a statistically significant difference in glomerular C3 fragment deposition compared to the PBS-treated PHN control group. All C3d-mAb-2fH-treated groups show a statistically significant reduction in C3 fragment deposition compared to their molar-equivalent dose of Fc-2fH (^+^p < 0.05, ^++^p < 0.005 vs. equivalent-dose Fc-fH_1–5_). (C) ADX-097 localization to glomeruli was detected by quantitation of anti-fH immunofluorescence. No ADX-097 is observed in healthy controls or in PHN rats dosed with PBS or CVF. Importantly, glomerular fH localization is also not detected in rats that received any dose of Fc-2fH_1–5_. In contrast, anti-fH immunofluorescence is measured at all doses of ADX-097. Staining is dose dependent across groups receiving s.c. ADX-097. However, i.v. dosing results in less glomerular localization that an equivalent dose delivered s.c. (compare 1 mg/kg s.c. vs. i.v. dosing groups), suggesting that the duration rather than the concentration of the *C*_max_ is a key determinant of ADX-097 accumulation in tissue (∗∗∗p < 0.0001). (D) On study day 5, ADX-097 treatment at doses ≥1 mg/kg showed similar reduction in proteinuria compared to CVF. At this time point, the 0.3 mg/kg group showed reduced uPCR, though less substantial than CVF or higher doses of ADX-097. At the end of the study (day 7), all ADX-097-treated groups exhibited a dose-dependent reduction in proteinuria (∗p < 0.01, ∗∗p < 0.005, ∗∗∗p < 0.0001 vs. anti-Fx1A + PBS). (E) Summary of uPCR_AUC_ for days 3–7 of study. (F) Time course of urine C5b-9/Cre ratio (uC5b-9) suggests that 1 mg/kg and 3 mg/kg doses of ADX-097 equivalently inhibit glomerular complement in the first 48 h after dosing but that effects on uC5b-9 are more durable in the 3 mg/kg dose group. AUC_uC5b-9_ of 1 and 3 mg/kg ADX-097 is reduced compared to the untreated anti-Fx1A dose group (p < 0.03 for 1 mg/kg s.c. and i.v.; p < 0.002 for 3 mg/kg s.c.). AUC_uC5b-9_ of the 0.3 mg/kg ADX-097 dose group and of the 0.17 and 0.57 mg/kg Fc-2fH dose groups are not statistically significantly different from that of untreated anti-Fx1A, indicating little effect of these treatments on uC5b-9. AUC_uC5b-9_ of the 1.7 mg/kg Fc-2fH dose group is reduced relative to untreated anti-Fx1A (p < 0.008), likely reflecting the longer circulating half-life of Fc-2fH. (G) Comparison of 3 mg/kg ADX-097 and the molar-equivalent dose of Fc-2fH_1–5_ (1.7 mg/kg). Both molecules reduce uPCR equivalently up to day 5, but by day 7 the effect of ADX-097 is more potent than that of Fc-2fH_1–5_ (^+^p < 0.05). (H) Comparison of 1 mg/kg s.c. or i.v. ADX-097 to 0.57 mg/kg s.c. Fc-2fH_1–5_. Both dosing routes of ADX-097 significantly reduce uPCR and uPCR_AUC_ (∗p < 0.01), while Fc-2fH_1–5_ has no statistically significant effect on either means of assessing proteinuria. (I) Comparison of 0.3 mg/kg s.c. ADX-097 and 0.17 mg/kg s.c. Fc-2fH_1–5_. Fc-2fH_1–5_ has no effect on proteinuria. ADX-097 shows a non-statistically significant trend toward proteinuria reduction.

In a separate study, we further explored ADX-097 potency in the PHN model using lower and s.c. doses of ADX-097 (0.3, 1, and 3 mg/kg) to understand the minimum efficacious dose (for study design see Figure 6A). We also included s.c. doses of Fc-2fH_1–5_ at molar equivalence to ADX-097 (0.17, 0.51, and 1.7 mg/kg, matching 0.3, 1, and 3 mg/kg ADX-097, respectively) to more directly compare C3d targeted vs. non-targeted complement inhibition.

In tissue collected 4 days after dosing (7 days after disease induction), we observed a statistically significant reduction in glomerular anti-C3 fragment immunofluorescence in rats dosed with 1 mg/kg or 3 mg/kg ADX-097 (Figure 6B). Treatment with equimolar doses of Fc-2fH_1–5_, however, showed no equivalent effect. Anti-fH immunostaining was detected in ADX-097-treated glomeruli but not in those that received Fc-2fH_1–5_ (Figure 6C), confirming a lack of fH_1–5_ localization in the absence of the C3d-targeting antibody.

Forty-eight hours after s.c. injection (5 days after disease induction), 1 mg/kg and 3 mg/kg doses of ADX-097 reduced uPCR relative to anti-Fx1A + PBS controls (p < 0.0007), while the 0.3 mg/kg ADX-097 group exhibited a non-statistically significant trend (p = 0.07) toward proteinuria reduction (Figure 6D). By 96 h after injection, all ADX-097 treatment groups exhibited a statistically significant reduction in proteinuria (p < 0.005) that correlated with ADX-097 dose (Figure 6D). A similar correlation was evident when uPCR was analyzed as AUC (uPCR_AUC_): Compared to anti-Fx1A + PBS, 3 mg/kg ADX-097 reduced uPCR_AUC_ by 73% ± 8.7% (p < 0.003), and 1 mg/kg (s.c.) reduced uPCR_AUC_ by 59% ± 10% (p < 0.01) (Figure 6E). uPCR_AUC_ reduction in the 0.3 mg/kg group (33% ± 22%) was not statistically significant, with the larger error reflecting a more variable response at this low dose. Efficacy of 3 mg/kg ADX-097 was similar to that of prophylactic CVF treatment (uPCR reduced by 76% ± 11%). We note, however, that despite daily dosing with CVF, the reduction of uPCR in this group waned between study days 5 and 6. This may be due to CVF’s high propensity to induce a neutralizing ADA response that can reduce circulating exposure.^74^^,^^75^^,^^76^ This interpretation is further supported by concomitant lack of glomerular complement inhibition in tissue (Figure 6B) and by urine C5b-9/Cre, which was similar to that of non-diseased control rats until study day 5 but elevated by day 7 (Figure 6F).

### Local complement inhibition preserved podocyte architecture

A subset of renal cortex samples collected on study day 7 was further analyzed by transmission electron microscopy (TEM) to visualize ultrastructural changes in the glomeruli. Figures S14A and S14B show representative TEM images from a healthy control glomerulus, with a GBM of uniform thickness and displaying a distinct lamina densa. Healthy podocytes with normal foot-process morphology and well-differentiated slit diaphragms resided along the length of the GBM (white arrows in Figures S14A and S14B). In contrast, representative glomeruli from rats treated with anti-Fx1A exhibited extensive podocyte foot-process effacement (yellow arrows in Figures S14C and S14D), with rare slit-diaphragm-like structures evident along a distorted GBM of varying thickness. Electron-dense subepithelial deposits were visible between some podocytes and the underlying GBM (yellow asterisks in Figure S14D), consistent with prior observations in the PHN model and representing accumulation of immune complexes at the filtration barrier.^62^ Figures S14E and S14F show representative images from a PHN rat treated with 3 mg/kg ADX-097. ADX-097 rescued podocyte architecture along a substantial portion of the GBM. While local examples of effaced podocytes (yellow arrows in Figures S14E and S14F) and electron-dense deposits (yellow asterisk in Figure S14F) were observed, the number of well-differentiated podocyte foot processes (white arrows in Figures S14E and S14F) was clearly increased throughout multiple evaluated samples. These data further demonstrate preservation of glomerular ultrastructure after ADX-097 treatment consistent with dose-dependent attenuation of uPCR.

### C3d-mediated drug targeting improved potency compared to non-targeted fH_1–5_

To further understand the relationship between potency and tissue targeting, we compared efficacy of ADX-097 to that of equimolar doses of Fc-2fH_1–5_. Forty-eight hours after s.c. delivery, 3 mg/kg ADX-097 and 1.7 mg/kg Fc-2fH_1–5_ equivalently reduced uPCR relative to controls, suggesting that both proteins initially inhibit renal injury. Fc-2fH_1–5_ exhibited 22- to 43-fold higher circulating exposure at end of the study compared to equimolar doses of ADX-097 (Figure S15B), a finding consistent with prior studies suggesting that C3d binding shortens the circulating half-life of anti-C3d antibodies and implying greater Fc-2fH_1–5_ systemic exposure across the course of the study.^77^ If the circulating *C*_max_ of both ADX-097 and Fc-2fH_1–5_ was high enough to inhibit circulating complement in this model, this effect would be prolonged in the 1.7 mg/kg Fc-2fH_1–5_ group, potentially explaining its proteinuria reduction. We note, however, that both 3 mg/kg and 1 mg/kg doses of ADX-097 inhibit urine C5b-9 more potently than 1.7 mg/kg Fc-2fH_1–5_ (Figure 6F), indicating that C3d targeting increased the potency of fH_1–5_ beyond any transient effects on fluid-phase/circulating complement. This also translated to greater durability as, 96 h after dosing, proteinuria in the 3 mg/kg ADX-097 group was less than in rats treated with 1.7 mg/kg Fc-2fH_1–5_ (p < 0.05) (Figure 6G). Thus, while complicated by potential effects on circulating complement, 3 mg/kg ADX-097 appears to be more potent than an equimolar dose of untargeted fH_1–5_.

Comparison of the 1 mg/kg ADX-097 (s.c. and i.v.) and 0.57 mg/kg Fc-2fH_1–5_ treatment groups revealed a more clear-cut efficacy difference, as ADX-097 but not Fc-2fH_1–5_ reduced uPCR and uPCR_AUC_ (Figure 6H). Notably, s.c. and i.v. routes show similar degrees of inhibition, indicating that circulating *C*_max_, which would be higher in the i.v. dosed group, had little effect on ADX-097 efficacy. A similar difference was observed between the 0.3 mg/kg ADX-097 and 0.17 mg/kg Fc-2fH_1–5_ treatment groups, although the difference between these low-dose groups is not statistically significant (Figure 6I).

Urine-soluble uC5b-9/Cre ratio further elucidated both the potency difference between C3d-mAb-2fH/ADX-097 and Fc-2fH_1–5_ (Figure 6F) and the kinetics of inhibition. Prior to dosing on day 3, uC5b-9/Cre was elevated in anti-Fx1A-treated rats (p < 0.02), and untreated and drug-treated dose groups showed no statistically significant difference in uC5b-9/Cre. In the first 48 h after dosing, 1 mg/kg and 3 mg/kg doses of ADX-097 equivalently inhibited glomerular complement, but effects on uC5b-9/Cre were more durable in the 3 mg/kg dose group, showing continued inhibition at the end of the study on day 7. AUC_uC5b-9_ of 1 mg/kg and 3 mg/kg ADX-097 was reduced relative to the untreated anti-Fx1A dose group (p < 0.03 for 1 mg/kg s.c. and i.v.; p < 0.002 for 3 mg/kg s.c.). AUC_uC5b-9_ of the 0.3 mg/kg ADX-097 dose group and of the 0.17 mg/kg and 0.57 mg/kg Fc-2fH dose groups were not statistically significantly different from that of untreated anti-Fx1A, indicating minimal effect of these treatments on uC5b-9. AUC_uC5b-9_ of the 1.7 mg/kg Fc-2fH dose group was reduced relative to untreated anti-Fx1A (p < 0.008), likely reflecting the longer circulating half-life of Fc-2fH as discussed above (Figure S15B). Thus, urinary C5b-9/Cre further underscores the potency advantage conferred by anti-C3d targeting of the fH_1–5_ moieties. Together with reduced proteinuria and potent inhibition of tissue complement deposition, these data demonstrate that ADX-097/C3d-mAb-2fH is a potent, durable, and local inhibitor of complement activation and effector generation at low doses, suggesting potential for therapeutic use in complement-mediated diseases.

## Discussion

Therapeutics that target systemic complement are constrained by complement’s essential role in innate immunity and by the fact that complement components exist in high abundance and undergo rapid systemic turnover.^15^^,^^16^^,^^17^^,^^21^^,^^22^ As a result, therapies relying on this strategy must contend with large pharmacologic sinks while striking a balance between safety and potency. Consequently a substantial unmet need remains, particularly in autoimmune diseases where standard of care includes immunosuppressive agents.^15^^,^^16^ Targeting drug to tissues where complement is dysregulated has the potential to improve potency by avoiding circulating sinks while minimizing systemic complement blockade, leaving complement-dependent homeostatic functions relatively intact. The data presented here suggest that localizing fH_1–5_ to tissue-deposited C3d is an effective strategy for targeted complement inhibition, resulting in potent and durable local complement inhibition at doses that do not affect systemic complement.

Translational studies presented here indicate that high-density C3d deposition is a feature of complement dysregulation across a range of tissues. We observed C3d immunostaining in both skin and kidney disease, including DLE, BP, MN, IgAN, and lupus nephritis (Figures 1 and S1). We note that C3d deposition co-localized with active complement (anti-C3c immunofluorescence), consistent with the fact that C3d is linked to complement activity rather than being tissue or organ specific. Thus, C3d targeting may have translational applications across a wide range of complement-driven diseases. While not the focus of this work, further analysis of associations between the density of C3d deposition and disease severity will be of interest. These findings support C3d as a localization target to bring a therapeutic to tissues with active complement. In addition, they suggest a translational strategy for C3d-mAb-2fH that focuses on indications with strong C3d deposition and/or patients for whom local C3d immunostaining is a part of their clinical diagnosis.

C3d-mAb-2fHs are human, mouse, and mouse/human chimeric antibody fusion proteins that direct fH_1–5_ to tissue-deposited C3d, thereby inhibiting local AP complement. We demonstrate in multiple models and species that these fusions localize to and regulate tissue complement sites of high C3d density, resulting in potent and durable local complement re-regulation at doses that do not affect systemic complement. In SPR studies, bivalent anti-C3d antibodies bound high-density C3d with approximately 2,000-fold greater affinity than monovalent Fabs (Figure 2B and Table 2), illustrating the significant avidity advantage of using a bivalent targeting antibody. Concordantly, even at low doses, C3d-mAb-2fH appeared to have a longer tissue-residence time than tissue-targeting strategies that rely on monovalent binding to their localization target.^48^^,^^51^^,^^59^ Furthermore, in addition to providing drug localization and durability, this avidity may also confer a binding preference toward high-density tissue C3d over lower-density C3d that is present on circulating blood cells.^78^^,^^79^^,^^80^

We also found that saturation of the C3d-binding target does not appear to be required for maximum local complement inhibition. In both mouse and NHP models, C3d-mAb-2fH was distributed to skin in a dose-dependent manner (Figures 3D, 4A–4C, and 4E) while effects on complement activation showed more limited dose dependence (Figures 3F, 4A–4C, and 4E). We believe this reflects the mechanism of C3d-mAb-2fH, in which affinity for high-density C3d durably holds the fH_1–5_ fragments in the vicinity of active complement. Because fH-mediated complement regulation is catalytic—a single molecule of C3d-mAb-fH can dissociate and degrade multiple convertase complexes—this long tissue-residence time greatly amplifies fH_1–5_-driven convertase decay and degradation. Furthermore, fH-induced degradation also leaves behind tissue-bound C3d, raising the possibility that C3d-mAb-2fH tissue localization may enhance its own convertase regulatory function either while the drug remains in circulation or upon subsequent dosing. These attributes combine to yield localized drug potency and durability that may be difficult to achieve through competitive or allosteric AP inhibition.

Importantly, our data suggest that localized inhibition is sufficient to control complement-driven disease. In the rat PHN model of renal injury, a single ≥1 mg/kg dose of human C3d-mAb-2fH (ADX-097) reduced the uPCR (Figure 5D). Accordingly, all doses of ADX-097 localized to glomeruli and significantly inhibited glomerular complement activity (anti-C3 fragment immunofluorescence) (Figures 5C, S11, and S12B). We also note that 1 mg/kg and 3 mg/kg doses yielded 40% and 75% decreases in glomerular anti-C3 fragment immunofluorescence, respectively, while their effect on proteinuria was comparable to fully blocking anti-C3 fragment immunostaining (Figure 5D). These data suggest that complete local complement inhibition may not be required to inhibit proteinuria. We cannot rule out, however, that some of the residual staining could represent inactive forms of C3, particularly iC3b.^48^^,^^59^ Generation of more selective C3 fragment antibodies may be required to address this question.

While all tested doses of ADX-097 inhibited glomerular complement, doses ≤10 mg/kg left serum activity intact by 48 h after dosing (Figures 5C and 5D), indicating that tissue-targeted complement inhibition is achievable with limited effect on serum complement. An additional study showed that 1 mg/kg ADX-097 (i.v. or s.c.) significantly reduced proteinuria and urine C5b-9, while an equimolar dose (0.57 mg/mL) of Fc-2fH_1–5_ had no effect (Figures 6E and 6I). ADX-097 at 3 mg/kg had a greater effect on urine C5b-9 and a more durable effect on proteinuria than equimolar Fc-2fH_1–5_ (1.7 mg/kg) (Figure 6F). Importantly, due to limitations on *in vivo* blood collection in the PHN model, we cannot rule out that circulating concentrations of ADX-097 may have transiently affected fluid-phase inhibition in these studies. However, an extensive PK/PD time course showed that 1 mg/kg and 5 mg/kg s.c. doses of C3d-mAb-2fH inhibit tissue complement in CfH^−/−^ mice without affecting intact C3 levels in plasma, a biomarker of circulating complement, even when plasma drug is at *C*_max_ (Figure 3). These data demonstrate a clear potency advantage of tissue targeting and furthermore suggest that local inhibition is sufficient for controlling complement-mediated disease.

Finally, treatment-induced changes in soluble urine C5b-9 in the PHN model provide important translational insight into this biomarker. Tissue deposition of C5b-9 is present in lupus nephritis, thrombotic microangiopathies, C3 glomerulopathy, and membranous, IgA, hypertensive, and diabetic nephropathies.^69^ Soluble C5b-9 has also been reported in urine (uC5b-9) in the rat PHN model and from patients suffering from IgAN, MN, and pre-eclampsia, suggesting that uC5b-9 may reflect tissue complement activity in the kidney.^70^^,^^71^^,^^72^^,^^73^ However, because serum-soluble C5b-9 is also elevated in disease,^81^^,^^82^^,^^83^ it has not been clear whether changes in urine C5b-9 stem from changes in the circulating biomarker or from intrinsic changes in tissue complement activation. We show here that urine-soluble C5b-9 tightly correlates with tissue-specific complement inhibition (Figure S13), suggesting that the bulk of urine C5b-9 in the PHN model stems directly from renal tissue rather than from changes in filtration of circulating soluble C5b-9. While additional studies in patients and in other model systems is warranted, this finding suggests that measurement of urine C5b-9 could be eventually be used in place of biopsy collection and immunostaining to track changes in tissue complement deposition.

Taken together, the data presented here describe a novel approach to local targeting of a complement-blocking therapy. Translational data confirm that high-density C3d deposition is a common feature of local complement activation across multiple tissue types, suggesting a therapeutic homing target as well as an opportunity for selecting patients who might most benefit from a C3d-directed inhibitor. Based on this, we designed mouse, human, and chimeric antibody fusion proteins that target fH_1–5_ to tissue complement via a high-affinity anti-C3d antibody (C3d-mAb-2fH). We show that this molecule efficiently localizes fH_1–5_ in human tissue *ex vivo* and *in vivo* in mouse, rat, and NHP models of complement activation. These data also elucidate key features of the C3d-mAb-2fH mechanism, suggesting that both antibody avidity and the catalytic mechanism of fH contribute to the potency and durability of the molecule. Critically, our data indicate that local complement inhibition is sufficient to alter disease progression. We also show that soluble urine C5b-9 (uC5b-9) closely reflects renal tissue complement inhibition in this model, suggesting uC5b-9 as a potential biomarker of local kidney complement activity. Collectively, these studies support further evaluation of the human C3d-mAb-2fH fusion protein ADX-097 in the clinic, with the potential for potent, durable, and targeted complement inhibition for a range of complement-driven diseases.

## Materials and methods

### Sourcing, immunostaining, and semiquantitative scoring of human samples

A retrospective study of human skin biopsies was carried out using formalin-fixed paraffin-embedded (FFPE) samples obtained from the Mass General Brigham Biobank (Boston, MA). After deparaffinization, antigen retrieval was accomplished using DAKO Target Retrieval Solution combined with heat treatment in a BioCare Medical Pressure Cooker. Slides were then blocked in a solution of 10% goat serum, 1% BSA, and 0.05% Na-azide in 1× PBS, followed by immunostaining for anti-C3d (clone 3d8b, mouse IgG_1_ kappa), followed by detection with an AF647-conjugated anti-mouse secondary or with a fluorescein isothiocyanate (FITC)-conjugated anti-C3c (DAKO F0201, rabbit IgG). Skin immunostaining was scored by a trained pathologist on a scale of 0–2 (negative staining was assigned as “0”). Thirty samples from healthy biopsies and a minimum of 18 samples for each disease state were included in this analysis.

Frozen human kidney biopsy tissue blocks were supplied by Arkana Laboratories (Little Rock, AR). Kidney tissue cryosections were stained with anti-C3d mAb (clone 3d8b, mouse IgG_1_ kappa) followed by a secondary FITC-conjugated goat anti-mouse IgG Fc. Fluorescence intensity of glomerular C3d staining was scored in blinded fashion by a pathologist for each case using a scale of 0–3+ (negative staining was assigned as “0” and trace staining as “0.5” in the plotted graphs). A subset of the human kidney samples used in this study was stained for C3 deposition using an FITC-conjugated goat anti-human C3 polyclonal antibody (#B1C/B1A; Kent Labs, Bellingham, WA). The fluorescence intensity of glomerular C3 staining was scored in blinded fashion by a pathologist for each case using a scale of 0–3+ (negative staining was assigned as “0” and trace staining as “0.5” in the graphs). A retrospective analysis was conducted based on the historical C3 scores to determine the frequency of C3-positive staining.

### Characterization of C3d binding

Binding affinity measurements were performed on Biacore 3000 or T200 at 25°C. Flow cells of the CM5 chip were coated with low surface density of human, cynomolgus monkey, or mouse C3d using a standard EDC/NHS amine coupling method in sodium acetate (pH 5.0). Anti-C3d antibodies and fusion proteins were diluted in buffer in a concentration series ranging between 0 and 200 nM (0–1,000 nM for CR2-fH_1–5_, due to its weaker binding affinity) and injected over surface-bound target antigen at a flow rate of 30 μL/min for 120 s followed by dissociation in running buffer for 180 s. Binding of antibody fusion proteins was monitored in real time and fit using a Langmuir (1:1) binding model. From the observed *k*_on_ and *k*_off_, *K*_D_ was determined. Surfaces were regenerated using two injections of glycine at pH 1.7 for 40 s.

### *In vitro* measurement of complement inhibition

#### Wieslab assays

Complement activation assays were performed using the Wieslab AP and CP ELISAs (SVAR Life Science, Malmö, Sweden) according to manufacturer’s instructions.

#### Classical pathway hemolysis (CH_50_) assay

Sheep red blood cells (SRBCs) were washed in gelatin veronal buffer with MgCl_2_/CaCl_2_ (GVB^++^) and isolated by centrifugation. Pelleted SRBCs were washed in GVB^++^, then sensitized with rabbit anti-sheep erythrocyte antiserum for 30 min at 30°C.^84^ Complement inhibition was assessed as decreased SRBC lysis vs. untreated controls.

### Complement deposition on human skin explants

Complement activation by BP immune complexes was performed as previously described.^55^ Compounds or a solvent were added with the addition of the complement source (diluted in normal human plasma). Slides were examined by fluorescence microscopy and image analysis in blinded fashion. Results are based on n = 10 per group, whereby the donor skin and patient IgG were varied.

### UVB-induced skin complement activation model in non-human primates

NHP UVB skin model was performed at Biomere (Worcester, MA). Twenty-four naive cynomolgus monkeys of 2–5 years of age were enrolled. Animals were sedated with Telazol, and UVB exposure at 3,120 mJ/cm^2^ was applied to dorsal skin on day −1 using a SolRx 100-series Ultraviolet Phototherapy Lamp Unit (SolarcSystems, ON, Canada). A piece of UV light blocking film with a center hole of 2-cm diameter was attached to the lamp unit surface to block all the UV light except the center 2-cm diameter area, so that only an area of the 2-cm circle was exposed to UVB at each skin testing site. Each animal received s.c. administration of ADX-097 or vehicle on day 0. Blood collections and skin biopsies were collected at specified time points (n = 3 per time point) (Figure 4A). Skin biopsies from naive animals were collected at the time of the final, group-specific time point. All animals were returned to the testing facility’s colony at the completion of post-biopsy medication and observations.

### NHP circulating PK/PD assays

Plasma PK analysis was conducted using an ADX-097-specific ELISA. Plates were coated with an anti-ideotype antibody that recognizes ADX-097 (Q32Bio CL00027) for total drug capture. Detection was accomplished using a biotinylated mouse anti-human factor H (Thermo Fisher [Waltham, MA] MA5-17735, clone OX-24) followed by a standard streptavidin HRP A/TMB colorimetric assay. Plasma complement activity assays were performed at Q32Bio using the Wieslab AP Kit (SVAR Life Science) according to manufacturer’s instructions. AP pathway activation was determined by normalizing values at each time point to pre-dose control.

### NHP skin immunostaining and image analysis

Frozen skin sections (5 μm) were stained after fixation with cold acetone at −20°C. Slides were rinsed with Dulbecco’s PBS and loaded onto a Leica BOND Rx autostainer. ADX-097 tissue drug level was detected using a biotinylated mouse anti-human factor H primary antibody (Thermo Fisher MA5-17735, clone OX-24) followed by detection with Alexa 647-conjugated streptavidin (Thermo Fisher S-21374). Slides were co-stained for complement fragment C3c using an FITC- rabbit anti-human C3c (DAKO F0201) antibody. Stained slides were mounted with VectaShield Vibrance anti-fade mounting medium containing DAPI (VectorLab H-1800). Whole slide images were acquired using DAPI, FITC, and Cy5 channels with identical exposure times in each channel across all slides. VisioPharm software was used to identify tissue edges and tissue-free areas to define a region of interest (ROI) corresponding to the epidermis. C3c and C3d signal in this ROI was quantified as an average signal intensity in the appropriate fluorescence channel.

### *CfH*^*−/−*^ mice

C57BL/6 fH-deficient (*CfH*^−/−^) mice carrying a targeted disruption of the gene encoding fH+ were generously provided by Prof. Matthew Pickering.^56^ Mice were housed and studies conducted at Istituto di Ricerche Farmacologiche Mario Negri IRCCS (Italy) or at Biomere (Worcester, MA) according to internal institutional guidelines.

### Rodent tissue immunostaining

Mouse or rat tissue samples (n = 5 per time point) were frozen in OCT. Cryosections (5 μm) were fixed in −20°C acetone. Rodent C3 fragments were detected using an FITC-conjugated goat anti-mouse C3 fragment polyclonal antibody (MPBiomedicals, 0855510) or an FITC-conjugated goat anti-rat C3 fragment polyclonal antibody (MPBiomedicals, 0855751). Rodent C3d was stained using a human anti-C3d IgG4 (clone 3d8b, ADX-086) followed by a secondary Alexa Fluor 647-conjugated mouse anti-human IgG4 pFc′ (Southern Biotech 9190-31 clone HP6023) or Alexa Fluor 488-conjugated mouse anti-human IgG4 pFc′ (Southern Biotech 9190-30 clone HP6023). Humanized C3d-mAb-2fH (ADX-097) was detected using FITC-conjugated OX-24 (Thermo Fisher MA5-17736). Mouse C3d-mAb-2fH (ADX-118) was detected using an FITC-conjugated mouse anti-mouse fH1-4 monoclonal antibody (clone 2A5, a gift from Dr. Claire Harris). Immunofluorescence quantitation was performed in blinded fashion using an EVOS M5000 imaging system (Thermo Fisher) and ImageJ software. A minimum of ten glomeruli were assessed per section.

### Measurement of plasma C3 in *CfH*^*−/−*^ mice

Mouse blood was collected by cardiac puncture in the presence of EDTA, chilled on ice, and plasma separated by centrifugation at 2,000 × *g* at 4°C within 15 min of collection. Mouse C3 was detected using a mouse C3 ELISA kit (Genway Biotech, San Diego, CA, GWB-7555C7) following the manufacturer’s instructions.

### Passive Heymann nephritis model of membranous nephropathy

PHN studies were performed at Inotiv Westminster (formerly Plato BioPharma). Six-week old male Sprague-Dawley rats (n = 10 per group, except for “no disease” control, which were n = 5 per group) were obtained from Charles River Laboratories and allowed to acclimatize for 5 days prior to study initiation. Rats were housed in metabolic cages throughout the study, starting at day 3. Nephritis was induced by administration of two doses of a sheep anti-Fx1A antibody (Dr. David Salant, Boston University School of Medicine, Boston, MA) delivered i.v. at 100 and 300 mg/kg on days 0 and 1 of the study, respectively. Healthy control animals were dosed i.v. with normal sheep serum (Millipore, Burlington, MA). The positive control group was treated intraperitoneally on day −1 with 150 U/kg CVF (Quidel, San Diego, CA), then 100 U/kg daily from day 0 until the end of the study. All other study animals were treated intraperitoneally with PBS on the same schedule. Test proteins ADX-097 and Fc-2fH_1–5_ or PBS were delivered either s.c. or i.v. on day 3. Urine was collected from day 2 to day 7, and body weights and other physiological parameters were assessed daily. At study termination, serum and plasma were collected and both kidneys collected for histology and immunofluorescence. Immunostaining and quantitation were performed as described above for mouse tissue. Urinary protein and creatinine were measured on an Olympus AU400e Clinical Chemistry Analyzer (Beckman Coulter, Brea, CA) using clinical chemistry reagents Micro-Total Protein and creatinine (Sekisui Diagnostics, Burlington, MA). Urinary albumin was measured by ELISA using a rat albumin-specific ELISA kit (Nephrat; Ethos Biosciences, Logan Township, NJ).

### Measurement of complement activity in rat serum

Blood was clotted in serum separator tubes, centrifuged, and stored at −80°C. Complement activity was measured using a modified zymosan assay protocol. Ten microliters of serum was mixed with 30 μL of PBS + 0.1% BSA buffer, after which 8.3% pre-activated zymosan (Complement Technologies, Tyler, TX), 16.6 mM EGTA, and 8.3 mM MgCl_2_ in 0.1% BSA/PBS was added to all wells (final volume, 100 μL). After incubation, the complement reaction was quenched with 20 μL of 50 mM EDTA and goat anti-rat C3-FITC (MP Biomedicals, Solon, OH) was added. Following washing, the pellets were resuspended in PBS (pH 7.4) and 0.1% BSA. Median fluorescence intensity values were measured to determine complement activity in the serum using an Attune flow cytometer (Thermo Fisher) collecting 10,000 events per well in autosampler mode, and were analyzed in FlowJo (FlowJo, Ashland, OR).

### Measurement of soluble C5b-9 in rat urine

Soluble C5b-9 was measured using a Hycult Terminal Complement Complex (HK-106) assay according to the manufacturer’s instructions, with the exception that measurement standard was diluted in diluent plus 25% male Sprague-Dawley urine (BioIVT #RAT00URINE0104496, Lot #RAT515719).

### Statistics

Statistical significance was determined by one-way ANOVA unless otherwise specified. All statistical calculations were made using embedded functions in GraphPad Prism software.

### Study approval

All animal study protocols were approved by and performed under guidelines of the institutional animal care and use committee of the institution where the studies were performed. Approval for collection and use of human samples was as follows. Collection of immune cells and skin samples for skin explant studies was approved by the Ethics Committee, University of Lübeck Retrospective (protocol numbers 09-140 and 04-061). Analysis of human kidney samples was performed under IRB 2017/06/28 (Arkana Laboratories, Little Rock, AR). Human skin specimens were retrieved from the archives of the Department of Pathology at Brigham and Women’s Hospital, and the study was conducted with approval of the Institutional Review Board of Brigham and Women’s Hospital, Harvard Medical School (2020P003508).

## Data and code availability

All data shown in this work are available from the authors upon request.

## Supplemental information

Document S1. Figures S1–S15 and Tables S1–S3

Document S2. Article plus supplemental information
