# Supplementary material for: C3d-Targeted factor H inhibits tissue complement in disease models and reduces glomerular injury without affecting circulating complement
Source: Mol Ther. 2024 Feb 20;32(4):1061–79. doi: 10.1016/j.ymthe.2024.02.001 (PMC11163200; doi:10.1016/j.ymthe.2024.02.001)
Supplement: Document S1. Figures S1–S15 and Tables S1–S3 [file mmc1.pdf]

YMTHE, Volume *32*

Supplemental Information

C3d-Targeted factor H inhibits tissue complement in disease models and reduces glomerular injury without affecting circulating complement

Fei Liu, Sarah T. Ryan, Kelly C. Fahnoe, Jennifer G. Morgan, Anne E. Cheung, Michael J. Storek, Alejandro Best, Hui A. Chen, Monica Locatelli, Shuyun Xu, Enno Schmidt, Leon F. Schmidt-Jiménez, Katja Bieber, Joel M. Henderson, Christine G. Lian, Admar Verschoor, Ralf J. Ludwig, Ariela Benigni, Giuseppe Remuzzi, David J. Salant, Susan L. Kalled, Joshua M. Thurman, V. Michael Holers, Shelia M. Violette, and Stefan Wawersik

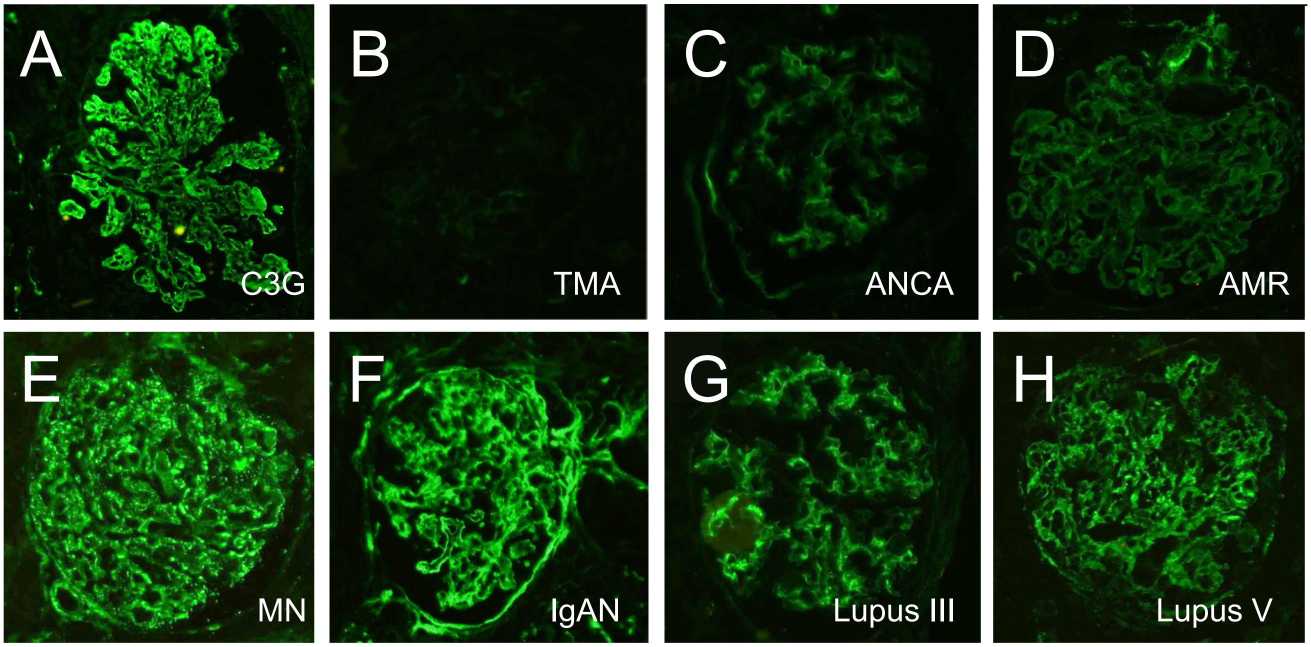

Figure S1. C3d Target Deposition in Human Glomerular Disease. Representative glomerular immunofluorescence in human biopsies from (A) C3 glomerulopathy (C3G), (B) Thrombotic microangiopathy (TMA), (C) anti-neutrophilic cytoplasmic autoantibody vasculitis (ANCA), (D) Antibody mediated rejection of kidney transplant (AMR), (E) Membranous glomerulopathy (MN), (F) IgA nephropathy (IgAN), (G) Class III and (H) Class IV lupus nephritis.

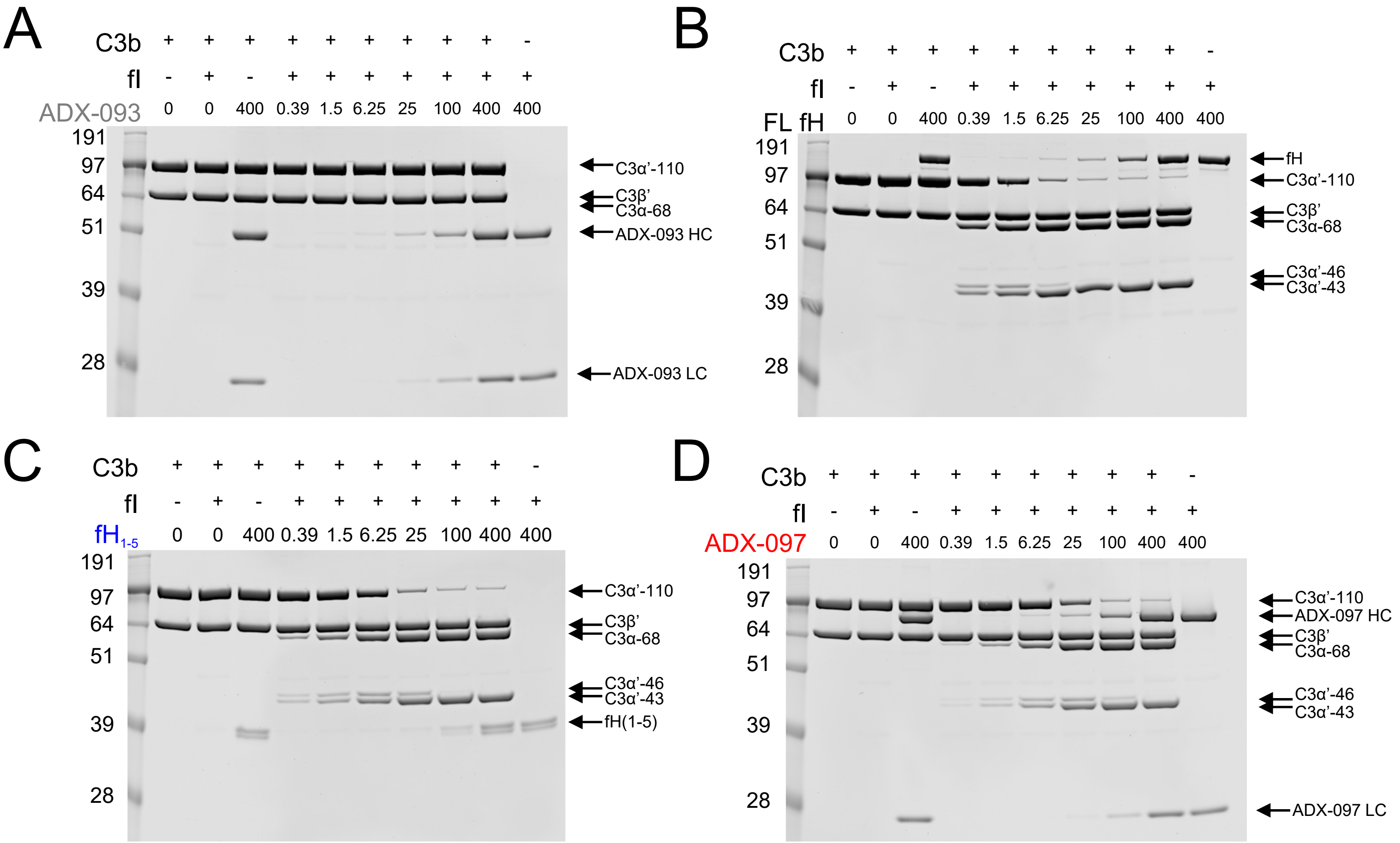

E

Concentration (nM)	% residual intact C3b			
	fH FL	ADX-097	fH1-5	ADX-093
0	100%	100%	100%	100%
0.39	71%	104%	95%	98%
1.56	44%	102%	86%	98%
6.25	16%	86%	60%	98%
25	11%	39%	21%	98%
100	9%	13%	14%	100%
400	6%	13%	13%	105%

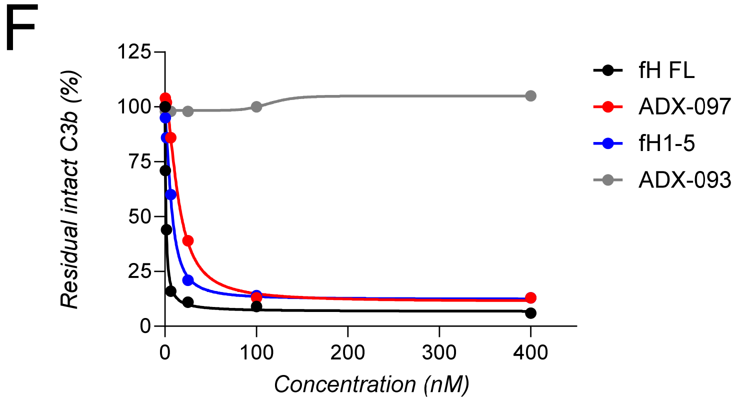

Figure S2. Effect of C3d-mAb-2fH on fI cofactor activity. C3b, fI, and increasing concentrations of (A) anti-C3d antibody (ADX-093), (B) full length Factor H (FL fH), (C) fH_{1-5}, or (D) human C3d-mAb-2fH (ADX-097) were incubated in solution at 37°C for 1 hour. C3b cleavage by fI was analyzed by SDS-PAGE followed by Coomassie staining. Disappearance of C3α'-110 band and the appearances of C3α'-68, -46, and -43 bands indicate C3b proteolytic inactivation. (E) % Residual C3b was calculated as the band intensities of the C3α'-110 band divided by the C3β' band, multiplied by 100. Band intensities were measured using imaging software. (F) % Residual C3b plotted as a function of fH (or control) concentration.

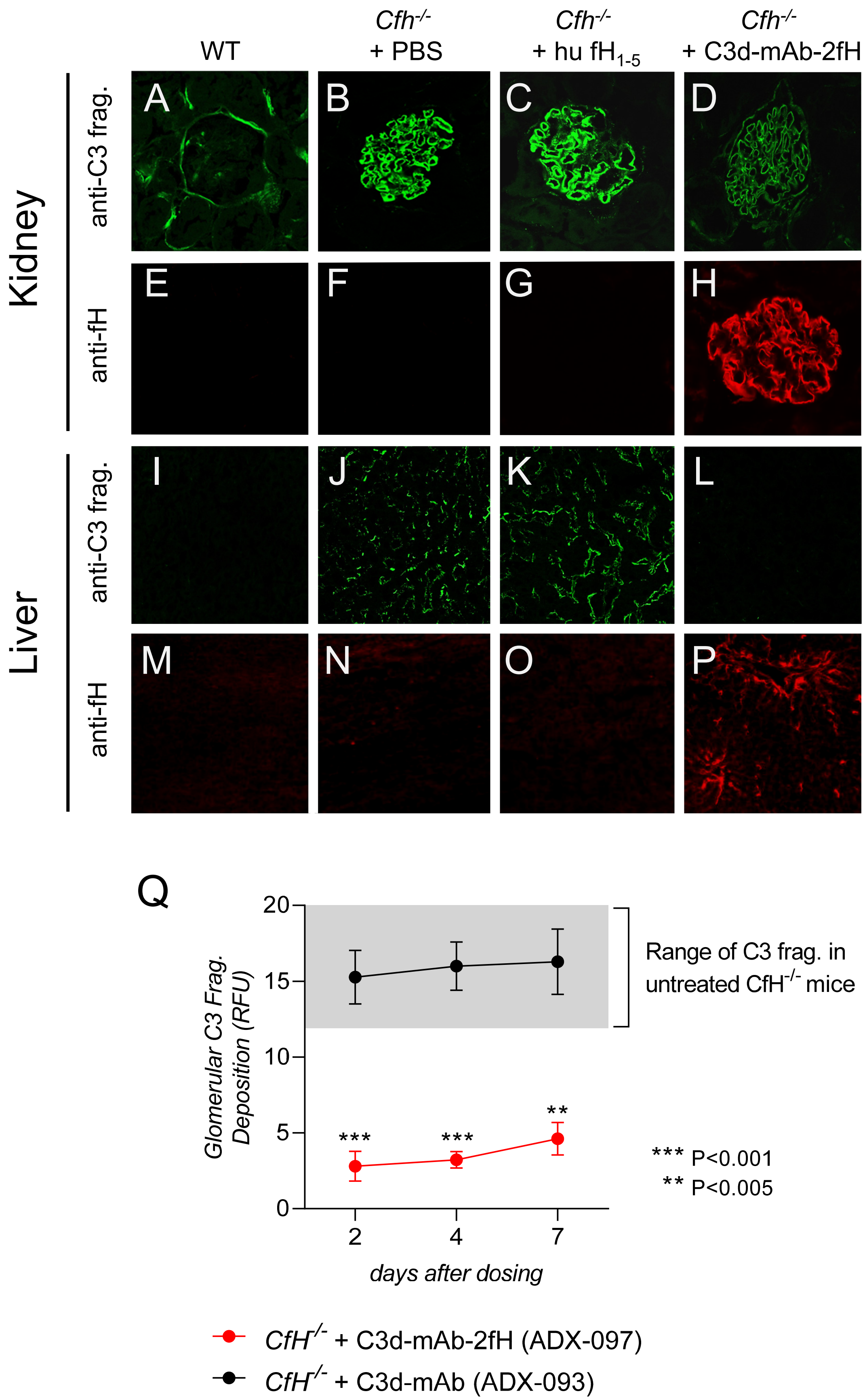
WT
Cfh-/- + PBS
Cfh-/- + hu fH1-5
Cfh-/- + C3d-mAb-2fH
Kidney
Liver
anti-C3 frag.
anti-fH
A
B
C
D
E
F
G
H
I
J
K
L
M
N
O
P
Q
Glomerular C3 Frag. Deposition (RFU)
days after dosing
Range of C3 frag. in untreated CfH-/- mice
*** P<0.001
** P<0.005
CfH-/- + C3d-mAb-2fH (ADX-097)
CfH-/- + C3d-mAb (ADX-093)

Figure S3. Tissue Localization and Complement Inhibition by C3d-mAb-2fH. A-P) *CfH*$^{-/-}$ mice were injected with PBS, 50 mg/kg IV ADX-048 (mouse/human chimeric C3d-mAb-2fH), or soluble human fH_{1-5} (16.6 mg/kg IV – 2x the molar equivalent to 50 mg/kg ADX-048 to provide similar number of fH_{1-5} molecules). Sections from kidney and liver tissue collected after 72 hours were immunostained for C3 active fragment (A-D and I-L) or for fH_{1-5} localization (anti-fH) (E-H and M-P). Background levels of C3 fragment deposition are detected in glomeruli (A) and liver (I) from wild-type mice, while increased C3 fragment deposition was detected in *CfH*$^{-/-}$ mice treated with PBS (B, J). C3 fragment deposition in kidney (C) or liver (K) was unaffected in mice treated with soluble human fH_{1-5}. However, C3 fragment deposition was qualitatively reduced in mice treated with C3d-mAb-2fH (ADX-048) (D, L). Consistent with this, fH_{1-5} localization was evident in C3d-mAb-2fH-treated mice (H, P), while no anti-fH immunofluorescence was detected in WT mice (E, M) or in *CfH*$^{-/-}$ mice treated with PBS (F, G) or soluble human fH_{1-5} (N, O). (Q) A time course of C3d-mAb-2fH-mediated tissue complement inhibition was in *CfH*$^{-/-}$ mice treated with 5 mg/kg IV ADX-097 (human C3d-mAb-2fH) or anti-C3d antibody (ADX-093) (n = 5 mice per group at each time point). Kidneys from *CfH*$^{-/-}$ mice (n = 3) were collected to define the range of C3 fragment deposition in untreated glomeruli (shaded area = average C3 fragment deposition +/- SEM). Kidney tissue was collected at 2, 4 and 7 days after dosing and immunostained with anti-C3 fragment antibody. Glomerular immunofluorescence from at least 10 glomeruli per animal was measured by digital image quantitation. At all time-points, C3d-mAb-2fH treatment significantly ($P < 0.005$) inhibited glomerular C3 fragment deposition. No difference was observed between PBS- and C3d-mAb-treated *CfH*$^{-/-}$ mice.

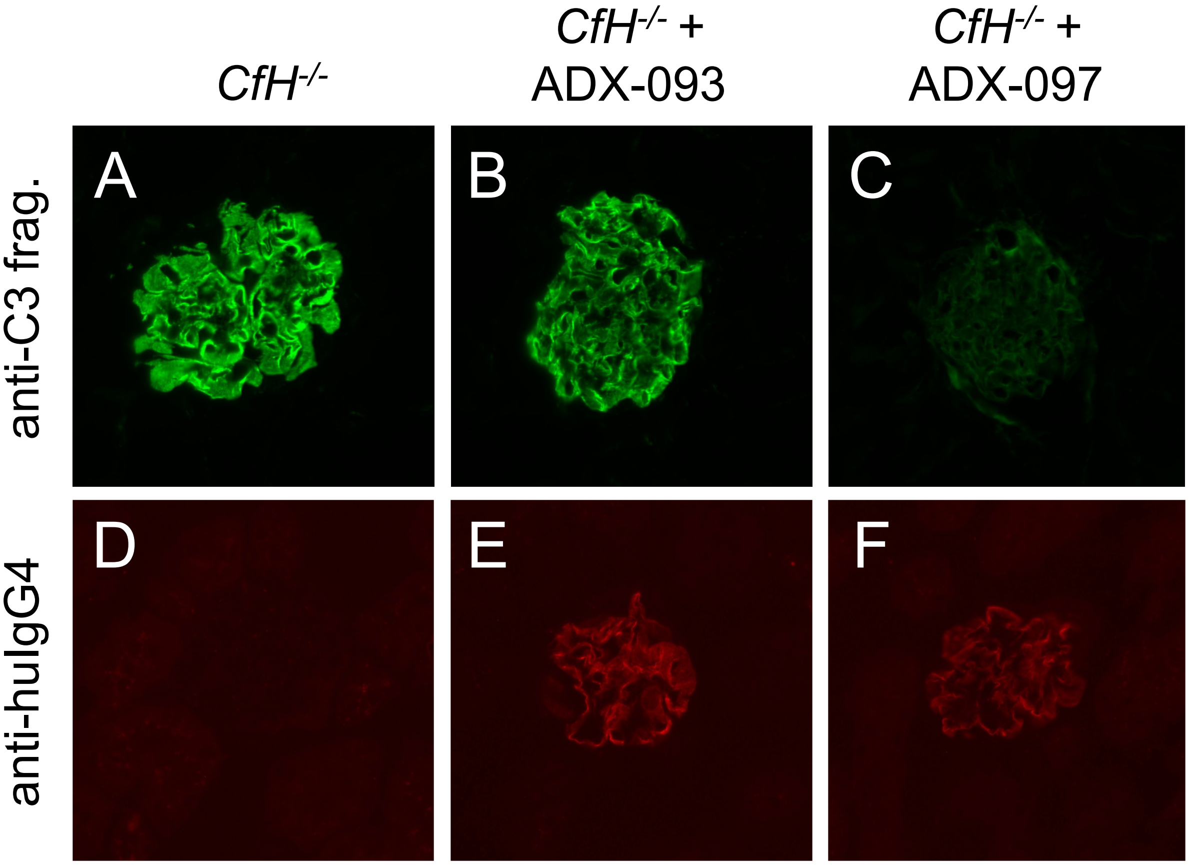

Figure S4. Localization and Tissue Complement Inhibition by ADX-097 and ADX-093 in CfH$^{-/-}$ Glomeruli. Representative images from kidneys immunostained with anti-C3 fragment antibodies. C3 fragment deposition was detected in untreated *CfH*$^{-/-}$ mice (A) and in *CfH*$^{-/-}$ mice treated with ADX-093 (B) or ADX-097 (C). anti-C3 fragment immunofluorescence images from all collected samples were used to generate quantitative data shown in Figure S4Q. To evaluate tissue drug localization, sections were immunostained with an anti-human IgG4 antibody that recognizes both ADX-093 and ADX-097. Representative immunofluorescence images are shown from untreated *CfH*$^{-/-}$ mice (D) and in *CfH*$^{-/-}$ mice treated with ADX-093 (E) or ADX-097 (F).

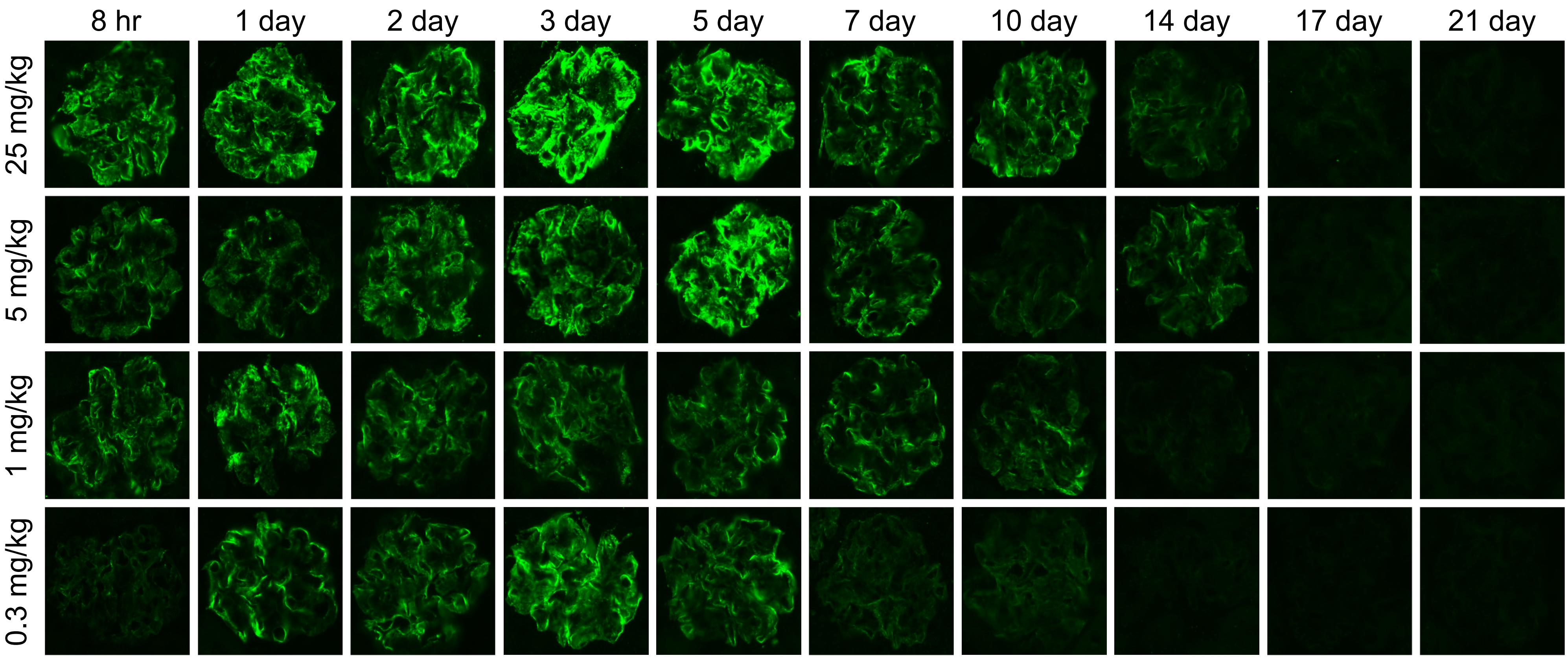

Figure S5. C3d-mAb-2fH Localization in *CfH*$^{-/-}$ Glomeruli. Representative immunofluorescence of fH localization in C3d-mAb-2fH (ADX-118)-treated *CfH*$^{-/-}$ mice. Images from all collected samples (n = 3-4 per time point) were used to generate quantitative data shown in Figure 4.

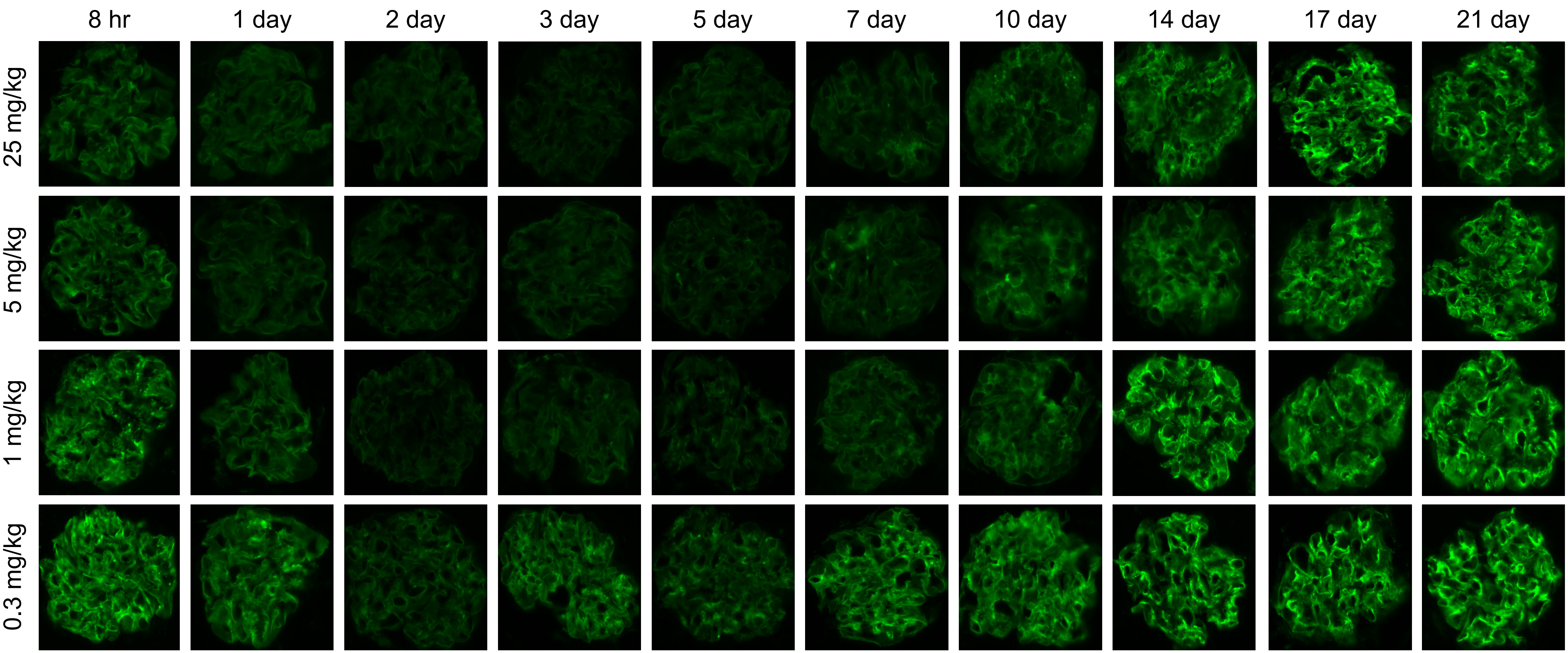

Figure S6. Tissue Complement Inhibition by C3d-mAb-2fH in *CfH*$^{-/-}$ Glomeruli. Representative immunofluorescence of C3 fragment deposition (anti-C3c antibody) in C3d-mAb-2fH (ADX-118)-treated *CfH*$^{-/-}$ mice. Images from all collected samples (n = 3-4 per time point) were used to generate quantitative data shown in Figure 4.

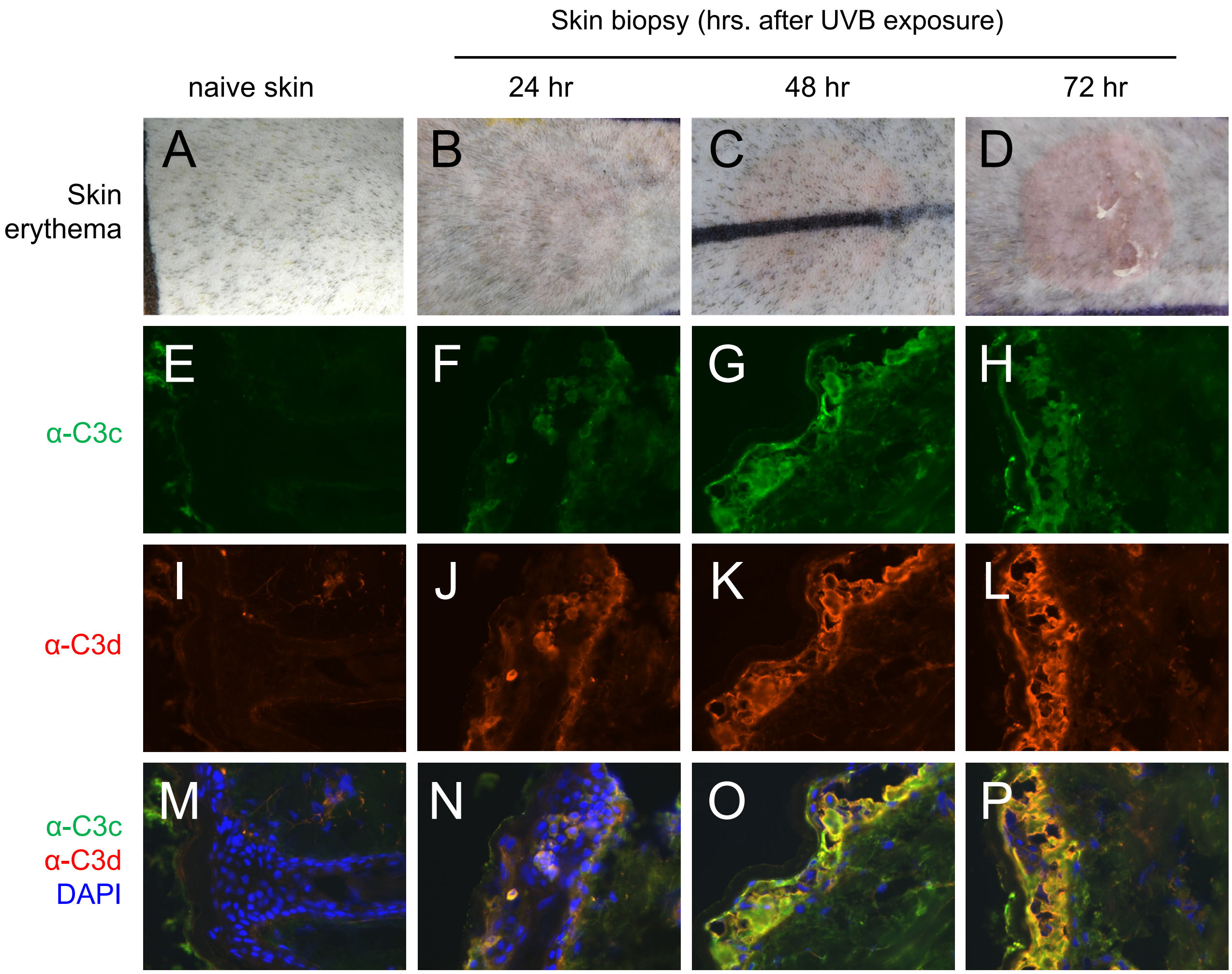

Figure S7. UVB-Induced Complement Activation in Cynomolgus Monkey Skin. Representative photographs of shaved cynomolgus monkey skin prior to exposure to UVB light (A) and 24 (B), 48 (C), and 72 hours (D) after UVB exposure. (E-H) anti-C3c immunofluorescence in naïve skin (E) and after UVB exposure (F-H) reveals the time course of complement activation in the model. (I-L) anti-C3d immunofluorescence shows minimal C3d deposition prior to UVB exposure (I). C3d is present in epidermis 24 hours after UVB exposure (J) and is maintained at 48 and 72 hours after exposure (K-L). anti-C3c (green) and anti-C3d (red) immunostaining co-localize in the epidermis (M-P)(biopsies were counterstained with DAPI (blue) to more clearly delineate tissue features.

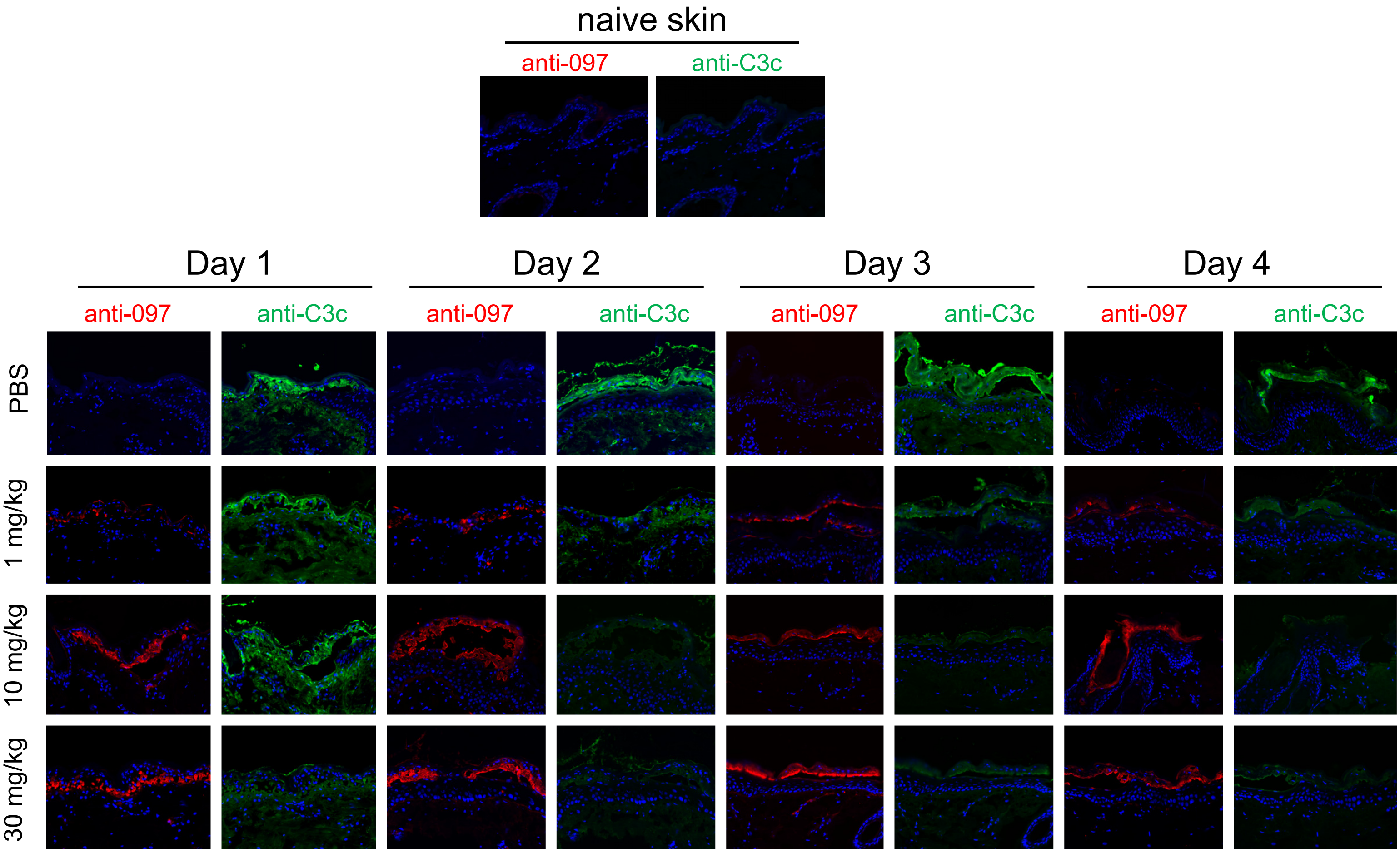

Figure S8. ADX-097 distributes to NHP skin and may inhibit local complement activation. Representative immunofluorescence of C3 fragment deposition (anti-C3c antibody, green) and C3d-mAb-2fH (ADX-097) localization (anti-fH immunostaining, red) in UVB-induced monkey skin. Images from all collected samples (n = 3 per time point) were used to generate quantitative data shown in Figure 3D, F.

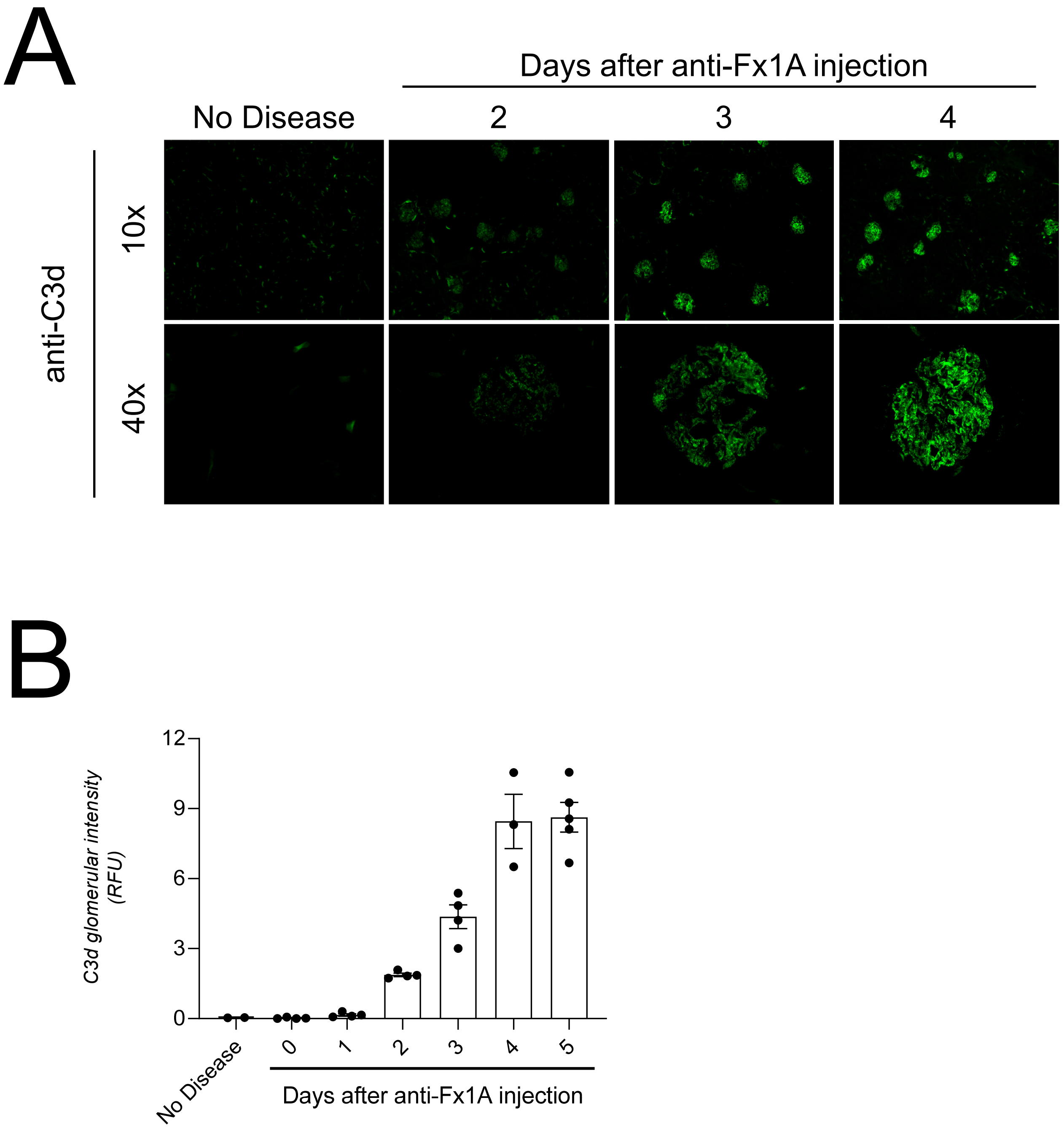

Figure S9. Characterization of C3d Deposition in Passive Heymann Nephritis. Kidney samples collected from the Passive Heymann Nephritis (PHN) model were collected and immunostained for C3d deposition. (A) Representative immunofluorescence from anti-C3d stained PHN kidneys shows clear C3d deposition in glomeruli by day 3 after anti-Fx1A-mediated disease induction. (B) Quantitation of C3d deposition in PHN glomeruli. Immunofluorescence from of least 10 glomeruli from 3-4 rats per time point was measured by digital image analysis.

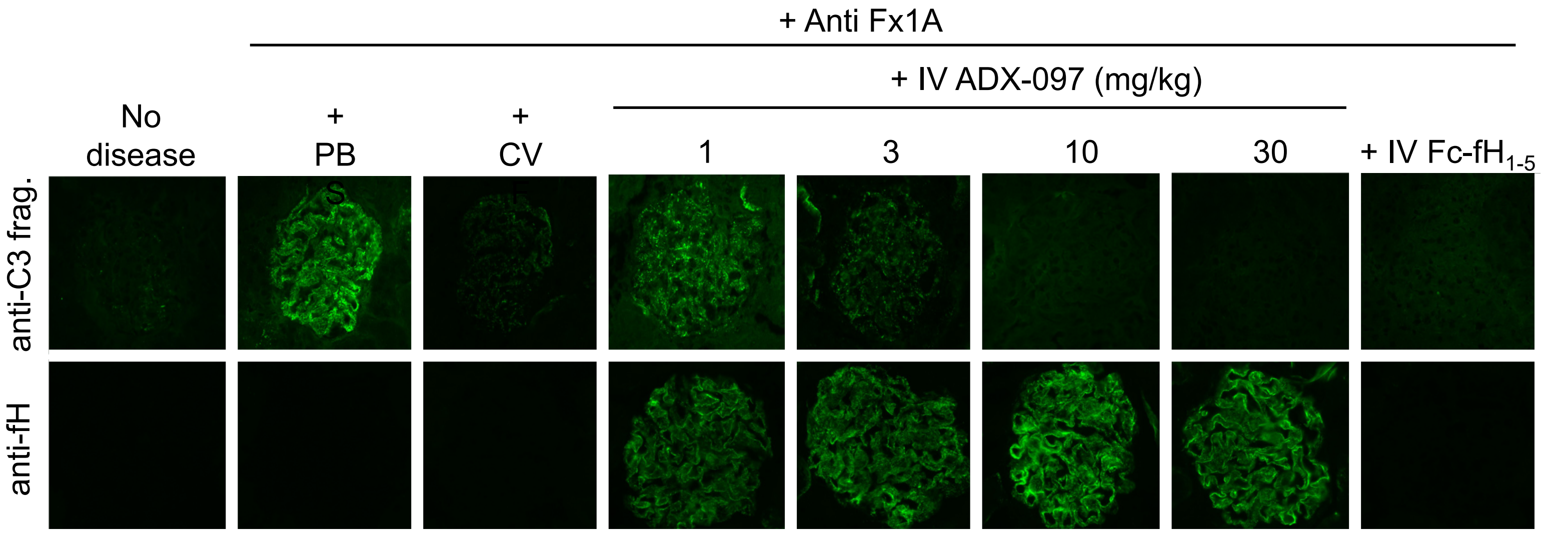

Figure S10. Representative Images of Tissue Complement Activity and ADX-097 Localization in Passive Heymann Nephritis. Images from all collected samples (n = 12 per group) were used to generate quantitative data shown in Figure 5C, D.

A

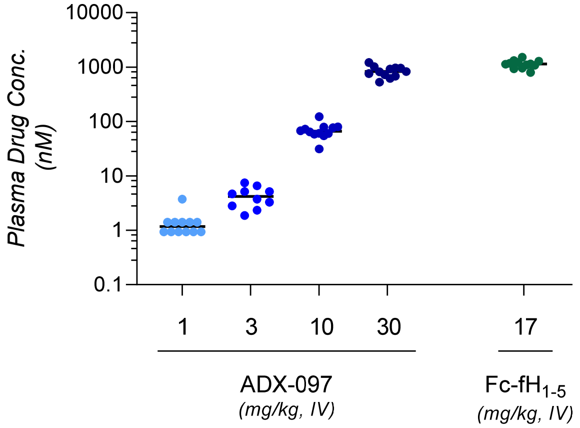

Treatment	Dose (mg/kg)	Ave Conc. (nM)	Ave Conc. (µg/ml)
ADX-097	30	837	181
ADX-097	10	69.0	14.9
ADX-097	3	4.31	0.93
ADX-097	1	1.36	0.29
Fc-2fH_{1-5}	17	1138	137

B

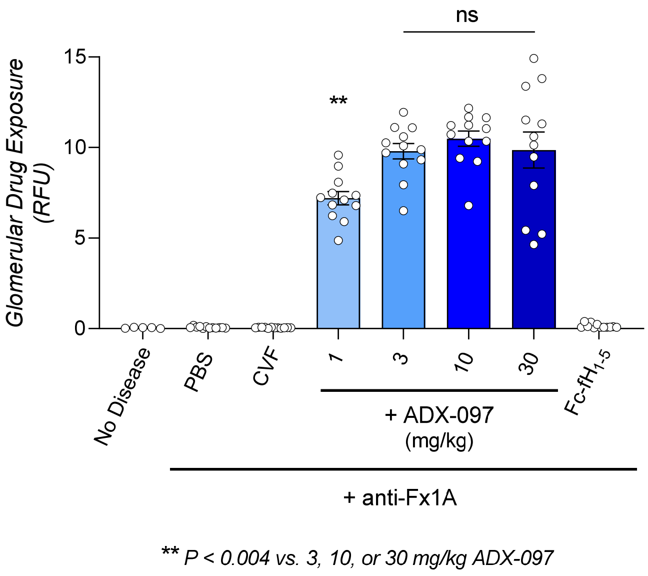

Figure S11. Tissue and Circulation Drug Exposure in Passive Heymann Nephritis. Kidney and plasma samples from the Passive Heymann Nephritis (PHN) study outlined in Figure 5A were collected on study day 5 (48 hours after ADX-097 treatment) analyzed for presence of ADX-097. (A) Plasma drug concentration was measured by drug-specific ELISA. Dose-dependent plasma drug concentrations are detected in the ADX-097 treatment groups. Fc-2fH_{1-5} circulating concentrations are similar to the 30 mg/kg ADX-097 dose group, consistent with delivery of equimolar doses of the two proteins. A summary of circulating drug concentrations, expressed in nM and µg/ml, are below. (B) Quantitation of glomerular immunofluorescence using an anti-fH antibody to detect localization of ADX-097. No anti-fH immunofluorescence is detected in non-disease controls, nor in PHN + PBS, PHN + CVF, or PHN + Fc-2fH_{1-5} dose groups. Dose-dependent localization of ADX-097 is detected in glomeruli, with less ADX-097 localization detected in glomeruli from the 1 mg/kg IV dose group. No difference in glomerular drug localization is observed between the 3, 10, and 30 mg/kg ADX-097 dose groups.

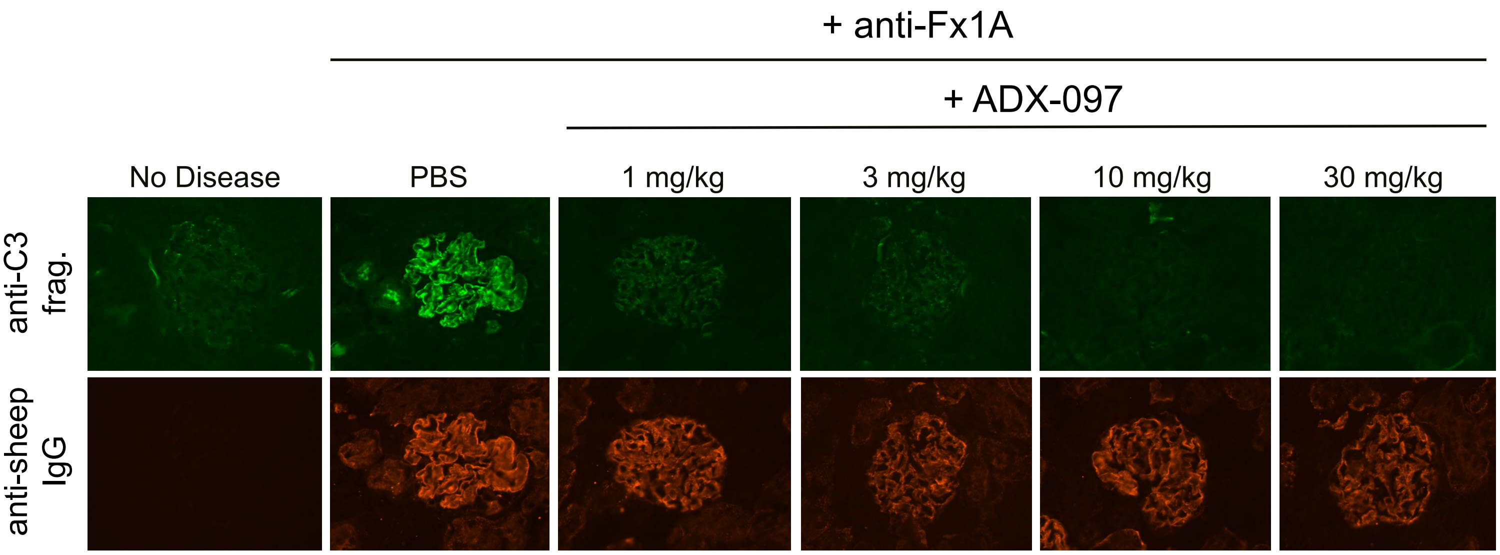

Figure S12. Anti-Sheep IgG Immunostaining in Passive Heymann Nephritis. Representative samples from Passive Heymann Nephritis (PHN) rats treated with indicated doses of C3d-mAb-2fH (ADX-097). Samples were co-stained for C3 fragment deposition (anti-C3c, green) and with an anti-sheep IgG antibody (red). ADX-097-mediated inhibition of C3 fragment deposition does not occur through blocking sheep anti-Fx1A, as anti-sheep IgG accumulation is similar in PHN + PBS controls and PHN + ADX-097-treated rats.

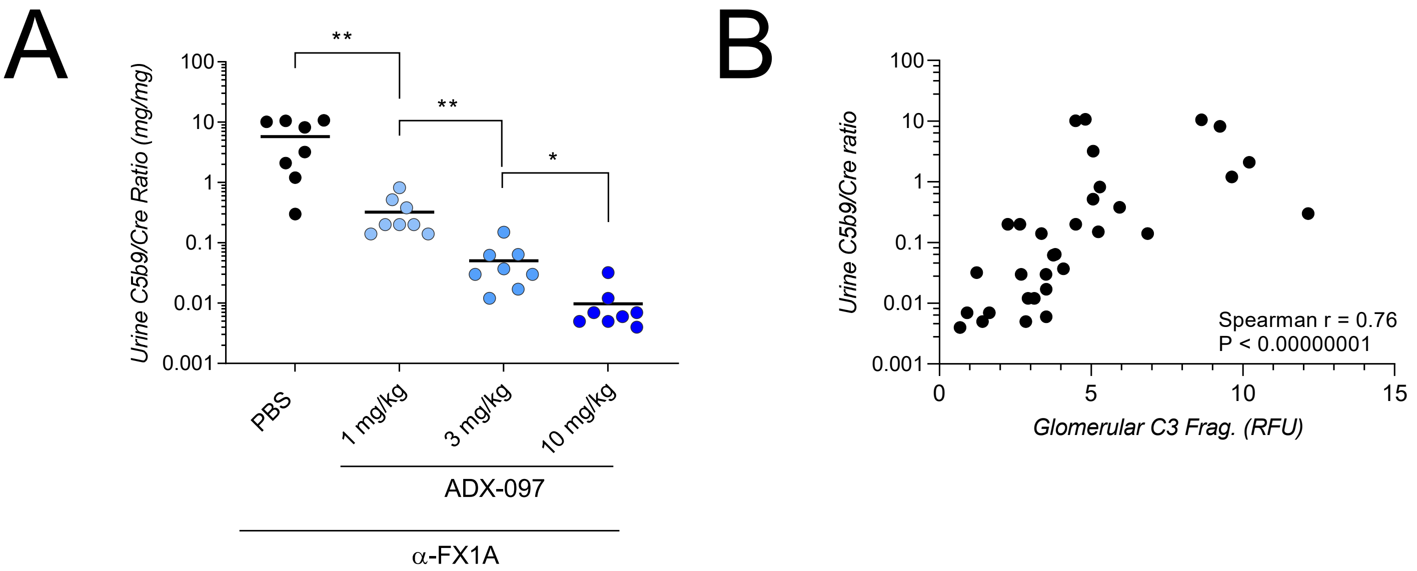

Figure S13. Urine C5b-9 Correlates with Glomerular Complement in Passive Heymann Nephritis. Soluble C5b-9 was measured in urine samples from study day 5 (48 hours after ADX-097 treatment) of the Passive Heymann Nephritis (PHN) study outlined in Figure 5A. (A) Urine C5b-9/Creatinine ratio is dose-dependently reduced in PHN rats after treatment with ADX-097 (**P < 0.002, * P < 0.03). Note that these doses (1 to 10 mg/kg, IV) do not inhibit circulating complement (see Figure 5D), suggesting that uC5b-9/Cre reflects changes in renal complement activity. (B) X-Y correlation plot of glomerular C3 fragment immunostaining on study day 5 vs. urine C5b-9/Cre ratio shows a strong correlation between glomerular complement deposition and urine C5b-9/Cre ratio (P < 0.00000001).

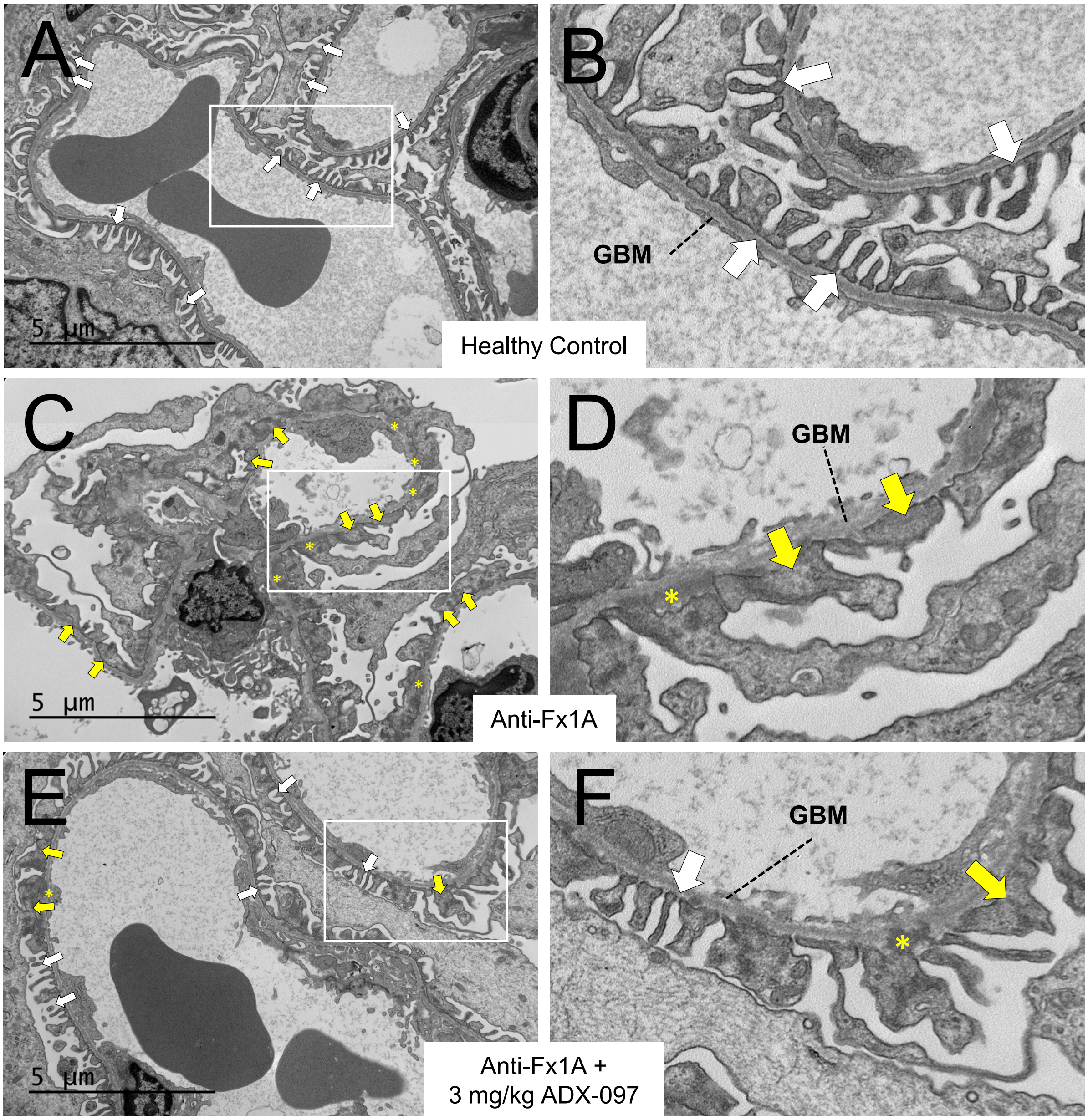

Figure S14. C3d-mAb-2fH Protects Podocyte Ultrastructure in Passive Heymann Nephritis. (A) Representative EM image from glomeruli in the healthy control (treated with normal serum) group shows well-differentiated podocyte foot processes (white arrows). (B) Enlarged image of the area outlined in the white frame in panel A. White arrows indicate examples of normal slit diaphragms. The glomerular basement membrane (GBM) is of uniform thickness with a distinct lamina densa (C) Representative glomerular EM image from a PHN rat shows extensive foot process effacement (yellow arrows), electron-dense regions consistent with immune complexes (yellow asterisks). (D) Enlarged image of the area outlined in the white box in panel B. Yellow arrows indicate effaced podocyte foot processes. Yellow asterisks denote electron-dense regions consistent with immune complex deposition. Note the distorted and thickened GBM without a clear lamina densa. (E) Glomerular EM from PHN rats treated with 3 mg/kg SC ADX-097 show substantial preservation of podocyte foot processes (white arrows), though occasional examples of effaced podocytes can be found (yellow arrows). (F) Enlarged image of the area within the white box in panel C. The white arrow highlights a representative healthy slit diaphragm, while the yellow arrow points out a partially effaced podocyte foot process. The GBM is more uniform in thickness and has a differentiated lamina densa.

A

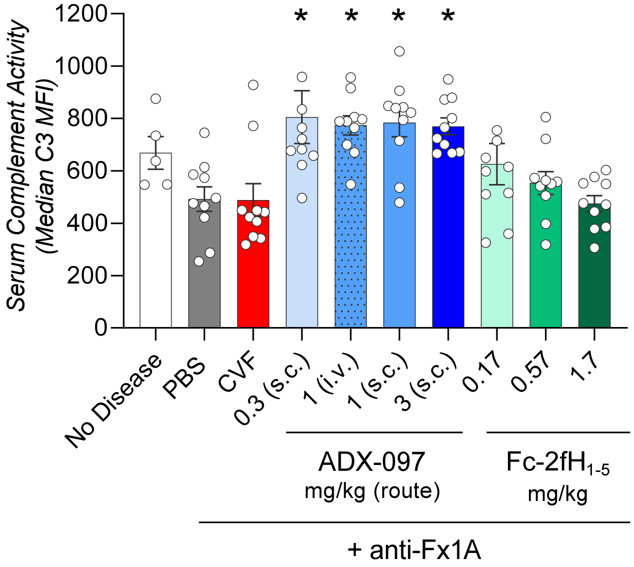

B

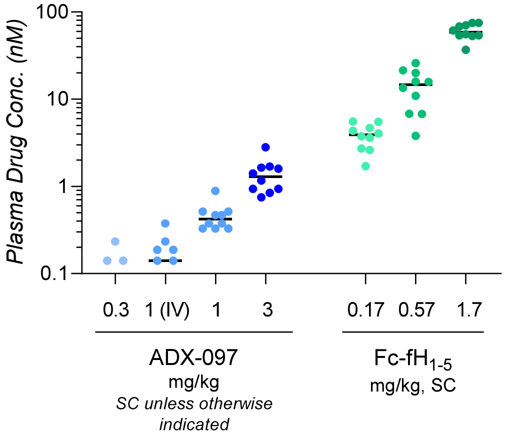

ADX-097 Dose (mg/kg)	Ave Plasma Conc. (nM)	Ave Plasma Conc. (µg/ml)	Fc-fH Dose (mg/kg)	Ave Plasma Conc. (nM)	Ave Plasma Conc. (µg/ml)
3	1.38	0.298	1.7	60.3	7.3
1	0.46	0.099	0.57	14.1	1.71
1 (IV)	0.16	0.035	-	-	-
0.3	0.12	0.026	0.17	3.85	0.465

Figure S15. Circulating Complement and Drug Exposure in Passive Heymann Nephritis. Kidney and plasma samples from the Passive Heymann Nephritis (PHN) study outlined in Figure 6A were collected on study day 7 (96 hours after ADX-097 treatment) analyzed for presence of ADX-097. (A) Day 7 serum complement activity measured by zymosan beads. The PHN + PBS control group shows a non-statistically significant reduction in serum complement activity compared to non-disease controls, suggesting that circulating complement levels may begin to drop as disease progresses. CVF- and Fc-2fH$_{1-5}$-treated PHN rats' serum complement activity is unchanged from PBS-treated controls. All doses of ADX-097 exhibit serum complement activity that is slightly higher than PHN + PBS controls ($P < 0.05$), indicating that ADX-097 is blocking glomerular complement activity without affecting systemic complement. (B) Plasma drug concentration was measured by drug-specific ELISA. Dose-dependent plasma drug concentrations are detected in the ADX-097 treatment groups. Fc-2fH$_{1-5}$ plasma concentrations are significantly higher at this time point, suggesting more rapid clearance of ADX-097 from circulation. A summary of circulating drug concentrations, expressed in nM and µg/ml, are below.

Supplemental Tables

Table S1. Semi-quantitative Scoring – Retrospective Review of Renal C3 Fragment Staining in Glomeruli

	TMA	ANCA	MN	IgAN	Lupus III	Lupus IV
Total Cases	100	104	109	107	112	107
# Positive	17	43	94	96	108	94
# Negative	83	61	15	11	4	13
% Positive	20.8	41.3	86.2	89.7	96.3	87.9
# of samples per positive score	9 = trace<br>2 = 1+<br>1 = 1.5+<br>4 = 1-2+<br>1 = 2-3+	13 = trace<br>9 = tr-1+<br>10 = 1+<br>7 = 1-2+<br>3 = 2+<br>1 = 3+	20 = trace<br>3 = tr-1+<br>32 = 1+<br>5 = 1-2+<br>21 = 2+<br>5 = 2-3+<br>9 = 3+	7 = trace<br>2 = tr-1+<br>31 = 1+<br>11 = 1-2+<br>31 = 2+<br>7 = 2-3+<br>6 = 3+	4 = trace<br>1 = tr-1+<br>24 = 1+<br>4 = 1-2+<br>42 = 2+<br>13 = 2-3+<br>20 = 3+	15 = trace<br>1 = tr-1+<br>27 = 1+<br>7 = 1-2+<br>24 = 2+<br>3 = 2-3+<br>17 = 3+

Table S2. Binding of Targeting Antibodies to C3d

Protein ID	Anti-C3d Antibody	Effector	Human C3d K_D (nM)	Cyno C3d K_D (nM)	Mouse C3d K_D (nM)
ADX-058 (3d8b)	Mouse (IgG1)	-	7.10	Not tested	6.90
ADX-093	Human IgG4 (humanized ADX-058)	-	10.0	Not tested	Not tested
ADX-118	ADX-058 (Mouse IgG1)	Mouse fH_{1-5}	9.40	1.33	7.16
ADX-097	ADX-093 (Human IgG4)	Human fH_{1-5}	12.0	6.32	3.15

Table S3. Comparison of C3d-mAb-fH Activity Across Species (Zymosan Assays)

Protein ID	Anti-C3d Antibody	Effector	Serum Species	IC_{50} (nM)
ADX-097	ADX-093 (Human IgG4)	2x Human fH_{1-5}	Mouse	202 ± 54
	ADX-093 (Human IgG4)	2x Human fH_{1-5}	Rat	99 ± 17
	ADX-093 (Human IgG4)	2x Human fH_{1-5}	Human	191 ± 17
ADX-118	ADX-058 (Mouse IgG1)	2x Mouse fH_{1-5}	Mouse	46 ± 3.9
	ADX-058 (Mouse IgG1)	2x Mouse fH_{1-5}	Rat	281 ± 52
	ADX-058 (Mouse IgG1)	2x Mouse fH_{1-5}	Human	No activity

Supplemental Methods

Humanization of 3d8b

The anti-C3d mAb 3d8b, originally identified as a murine IgG2, [42] was humanized as previously described [43] using modeled structure-based complementarity-determining region (CDR) grafting into human germline gene acceptor frameworks [81]. The resulting antibody is a hinge-stabilized (S228P) human IgG4 with additional Fc mutations to minimize C1q binding and effector function.

Generation and expression of fusion proteins

Anti-C3d parental antibodies, antibody, Fab, and CR2 fusions, and Fc-fusions were transiently transfected in CHO cells using standard methods. Proteins were affinity purified over Protein A (Cytiva, Marlborough, MA) and buffer exchanged into phosphate buffered saline (PBS) pH 7.4 using size exclusion chromatography to yield material with greater than 95% purity and endotoxin levels below 0.5 EU/mg. Proteins were concentration by centrifugation through high flow polyethersulfone (PES) membranes and sterile filtered through 0.2 µm filters.

Fluid phase fI co-factor activity

Complement regulatory activity of C3d-mAb-2fH was assayed in a fluid phase fI co-factor activity assay as previously described. [47,48] 0.7 µM of C3b and 20 nM of fI were mixed with either full length fH (FL fH), fH_{1-5}, ADX-097 (human C3d-mAb-2fH), or ADX-093 (human anti-C3d binding antibody) in a total volume of 16 µl in PBS buffer at 37°C for 30 min. The proteolytic breakdown of C3b was assessed using a 10% SDS-PAGE gel followed by Coomassie

staining. C3b cleavage was calculated by measuring the band intensity ratio of C3α’-110/C3β’ using a LI-COR gel imager and associated software.

Zymosan Complement Activation Assay

Pre-activated zymosan (Complement Technologies, Tyler, TX) was diluted in PBS pH 7.4 containing 25 mM EGTA, 12.5 mM $MgCl_2$ and 0.1% BSA. Antibody fusion proteins were serially diluted in PBS pH 7.4, 0.1% BSA. Activated zymosan was combined with fusion protein and 25% complement preserved serum. Reactions were incubated at 37°C for 20 minutes and stopped by the addition of 50 mM EDTA. Samples were centrifuged (3000 rpm), and the zymosan pellet was resuspended and washed in PBS pH 7.4, 0.1% BSA followed by centrifugation. The final pellet was resuspended in goat anti mouse C3–fluorescein isothiocyanate (FITC) $F(ab')_2$ (MP Biomedicals, Solon, OH) in PBS, pH 7.4 and incubated for 1 hour on ice. Following washing and centrifugation as described above, the pellets were resuspended in PBS, pH 7.4, 0.1% BSA. Data were acquired on an Attune flow cytometer (ThermoFisher, Waltham, MA) and analysed in FlowJo (FlowJo, LLC, Ashland, OR).

Plasma drug exposure assays (C3d-mAb-2fH and Fc-2fH)

C3d-mAb-2fH proteins in plasma were measured by ELISA. Human C3d protein (Complement Technologies, Tyler TX) was coated onto plates, then blocked in 1% BSA/TBS-Tween. Standards were diluted into 2% mouse or rat plasma and study samples were diluted in TBS. Biotin conjugated anti-human Factor H antibody (OX-24-biotin, ThermoFisher, Waltham MA) was added and subsequently detected with streptavidin – HRP (BioLegend San Diego, CA). The reaction was stopped with 2 N Sulfuric Acid Stop Solution (R&D systems, Minneapolis, MN)

and plates were read on a SpectraMax 250 plate reader. A four-parameter fit was used to generate the standard curve and test samples values were extrapolated from the standard curve. Fc-2fH protein was measured in plasma by coating plates with anti-human Factor H antibody (OX-24, ThermoFisher, Waltham, MA) and blocked in 2% BSA/PBS. Standards and test samples were diluted into 2% rat plasma/PBS. Drug binding was detected using an HRP-anti human IgG4 Fc' (Abcam, Waltham, MA) followed by TMB (ThermoFisher, Waltham, MA), then stopped with stop solution (ThermoFisher, Waltham, MA) and read on a SpectraMax 250 instrument. Standards were fit to a four-parameter curves and test sample concentrations were extrapolated using GraphPad Prism software.

Transmission Electron Microscopy

Kidney tissue was fixed with 1% osmium tetroxide in 0.15M cacodylate buffer, dehydrated in an acetone series, and embedded in epoxy resin. Ultrathin sections were cut at 72 nm with a diamond knife, mounted on 200-mesh copper grids, and stained with 4% uranyl acetate and 0.4% lead citrate. Prepared sections were examined in a JEOL JEM-1010 transmission electron microscope, and digital images were collected at a range of magnifications with an Erlangshen ES100W digital camera (Gatan, Pleasanton,CA).
