# Supplementary material for: C3d-Targeted factor H inhibits tissue complement in disease models and reduces glomerular injury without affecting circulating complement
Source: Mol Ther. 2024 Feb 20;32(4):1061–79. doi: 10.1016/j.ymthe.2024.02.001 (PMC11163200; doi:10.1016/j.ymthe.2024.02.001)
Supplement: Document S2. Article plus supplemental information [file mmc2.pdf]

Molecular Therapy
Original Article

C3d-Targeted factor H inhibits tissue complement in disease models and reduces glomerular injury without affecting circulating complement

Fei Liu,[1,12] Sarah T. Ryan,[1,12] Kelly C. Fahnoe,[1] Jennifer G. Morgan,[1] Anne E. Cheung,[1] Michael J. Storek,[1,11] Alejandro Best,[2] Hui A. Chen,[3] Monica Locatelli,[4] Shuyun Xu,[5] Enno Schmidt,[6] Leon F. Schmidt-Jiménez,[6] Katja Bieber,[6] Joel M. Henderson,[3] Christine G. Lian,[5] Admar Verschoor,[7,8] Ralf J. Ludwig,[6] Ariela Benigni,[4] Giuseppe Remuzzi,[4] David J. Salant,[9] Susan L. Kalled,[1] Joshua M. Thurman,[10,11] V. Michael Holers,[10] Shelia M. Violette,[1] and Stefan Wawersik[1]

[1]Q32 Bio, Waltham, MA 02451, USA; [2]Arkana Laboratories, Little Rock, AR 77211, USA; [3]Department of Pathology and Laboratory Medicine, Chobanian and Avedisian School of Medicine at Boston University and Boston Medical Center, Boston, MA 02118, USA; [4]Istituto di Ricerche Farmacologiche Mario Negri IRCCS, Centro Anna Maria Astori, Science and Technology Park Kilometro Rosso, 24126 Bergamo, Italy; [5]Department of Pathology, Brigham & Women's Hospital/Harvard Medical School, Boston, MA 02115, USA; [6]Lübeck Institute of Experimental Dermatology, University of Lübeck, 23562 Lübeck, Germany; [7]Department of Otorhinolaryngology, Technische Universität München and Klinikum Rechts der Isar, 81675 Munich, Germany; [8]Department of Dermatology, University Hospital Schleswig-Holstein, University of Lübeck, 23562 Lübeck, Germany; [9]Department of Medicine, Chobanian and Avedisian School of Medicine at Boston University and Section of Nephrology, Boston Medical Center, Boston, MA 02118, USA; [10]Department of Medicine, University of Colorado School of Medicine, Anschutz Medical Campus, Aurora, CO 80045, USA

Complement-mediated diseases can be treated using systemic inhibitors. However, complement components are abundant in circulation, affecting systemic inhibitors' exposure and efficacy. Furthermore, because of complement's essential role in immunity, systemic treatments raise infection risk in patients. To address these challenges, we developed antibody fusion proteins combining the alternative-pathway complement inhibitor factor H (fH_{1-5}) with an anti-C3d monoclonal antibody (C3d-mAb-2fH). Because C3d is deposited at sites of complement activity, this molecule localizes to tissue complement while minimizing circulating complement engagement. These fusion proteins bind to deposited complement in diseased human skin sections and localize to activated complement in a primate skin injury model. We further explored the pharmacology of C3d-mAb-2fH proteins in rodent models with robust tissue complement activation. Doses of C3d-mAb-2fH >1 mg/kg achieved >75% tissue complement inhibition in mouse and rat injury models while avoiding circulating complement blockade. Glomerular-specific complement inhibition reduced proteinuria and preserved podocyte foot-process architecture in rat membranous nephropathy, indicating disease-modifying efficacy. These data indicate that targeting local tissue complement results in durable and efficacious complement blockade in skin and kidney while avoiding systemic inhibition, suggesting broad applicability of this approach in treating a range of complement-mediated diseases.

INTRODUCTION

Complement is an essential component of immunity, providing a first line of defense against pathogens and a bridge between the innate and adaptive immune systems.[1–3] The complement cascade can be initiated through either mannose-binding lectins (lectin pathway [LP]) or by immunoglobulin M (IgM) or IgG clustering (classical pathway [CP]).[3] A third arm, the alternative pathway (AP), amplifies CP- and LP-initiated complement activation.[4–7] All three pathways trigger formation of protein complexes called convertases that proteolytically cleave complement C3 and C5 proteins into functional fragments. Complement activation drives multiple important immune and homeostatic functions. These include promoting cell lysis and pro-inflammatory signaling through membrane attack complex (MAC) formation, activating phagocytosis through target opsonization, attracting phagocytes by generating C3a and C5a chemotactic peptides, increased expression of C3a and C5a receptors on selected cell types, and stimulation of B cells, T cells, and follicular dendritic cells.[1–3,8]

Persistent uncontrolled complement activation plays a major role in the pathogenesis of several inflammatory and autoimmune diseases.[9] Systemic complement blockade—reducing dysregulated tissue complement via circulating complement inhibitors—has consequently garnered significant attention as a therapy for diseases including paroxysmal nocturnal hemoglobinuria, atypical hemolytic uremic syndrome, cold agglutinin disease, C3 glomerulopathy

Received 30 June 2023; accepted 1 February 2024;
https://doi.org/10.1016/j.ymthe.2024.02.001.

[11]Present address: Sanofi, Cambridge, MA 02141, USA
[12]These authors contributed equally
Correspondence: Stefan Wawersik, Q32 Bio, 830 Winter St., Waltham, MA 02451, USA.
E-mail: swawersik@q32bio.com

This is an open access article under the CC BY license (http://creativecommons.org/licenses/by/4.0/).

(C3G), IgA nephropathy (IgAN), bullous pemphigoid (BP), and geographic atrophy.[10–14] However, because of its essential role in innate immunity, systemic complement inhibition through long-half-life circulating inhibitors increases susceptibility to bacterial infections, including life-threatening meningococcal (*Neisseria meningitidis*) infection and sepsis, even in vaccinated patients.[15–17] One approach to mitigating this risk has been to selectively inhibit downstream terminal complement activation, for example by blocking C5 cleavage, thereby leaving proximal complement intact.[18] However, recent data in membranous nephropathy (MN) indicate that C3 activation and AP amplification play a major role in disease by upregulating C3aR1 and C5aR1 expression on podocytes, suggesting that inhibition of proximal complement may be more effective than distal blockade.[8,19,20]

Circulating complement inhibitors also face a second challenge: many complement proteins are highly abundant in circulation and undergo rapid turnover, creating a large pharmacologic sink. Thus, efficacy of many systemically acting complement therapeutics requires high circulating concentrations, and patients can experience suboptimal disease control when these concentrations are not achieved or maintained.[21–23] Furthermore, these drugs often must be delivered in large volumes and high doses, requiring biweekly or monthly infusions by a trained healthcare provider, adding to patient burden, treatment cost, and risks of short- and long-term toxicity.[24–28] These factors illustrate the difficult balance between drug exposures high enough to effectively inhibit complement but low enough to minimize patients' infection risk, and substantial unmet need remains for safer and more effective anti-complement therapies.

Many complement-mediated diseases are characterized by highly localized complement activation.[29,30] Concentrating an inhibitor at sites of pathogenic active complement could therefore address the challenges of circulating complement blockade. Such a tissue-targeted drug could avoid pharmacologic sinks, thereby improving potency. Furthermore, this approach may enable durable complement control in tissues after the circulating drug has cleared, leaving the complement system largely intact in unaffected tissues and in circulation, thereby potentially improving safety.

To test the feasibility of this strategy, we explored use of targeted human and rodent bifunctional fusion proteins to locally deliver an active fragment of factor H (fH), a critical negative regulator of AP complement that is predominantly synthesized in liver.[31,32] In both in the fluid phase and on surfaces, fH controls the AP amplification loop by catalyzing dissociation of the AP convertases and irreversible proteolytic degradation of convertase components.[31,33] Five N-terminal short consensus repeats (SCRs) of fH ($fH_{1–5}$) are sufficient for both AP convertase dissociation and degradation.[34,35] We show that $fH_{1–5}$ localization to high-density surface-bound AP convertases can be achieved by fusion to a monoclonal antibody that recognizes a common epitope with the complement fragments iC3b, C3dg, and C3d, which are covalently deposited at high density on tissues where complement is active.[36–39] For simplicity, we refer to this antibody as "anti-C3d" and the human and rodent fusion proteins collectively as C3d-mAb-2fH.

Together, the data presented here demonstrate three important attributes of this C3d-targeted strategy: First, tissue C3d deposition co-localizes with active complement in multiple autoimmune kidney and skin conditions, supporting a translational strategy that focuses on the presence of C3d in diseased tissue. Second, we demonstrate that high-affinity anti-C3d antibody binding can efficiently localize a complement regulator in multiple tissues and across species. Finally, in the passive Heymann nephritis (PHN) antibody-driven model of membranous nephropathy in rats, we show that local complement inhibition is sufficient to modulate disease progression *in vivo*. C3d-mAb-2fH fusion proteins in this model inhibited glomerular complement and progression of renal injury without affecting systemic complement. These studies therefore point to the therapeutic potential of a humanized C3d-mAb-2fH, ADX-097, suggesting C3d-directed targeting as a strategy for potent, localized complement inhibitors.

RESULTS

C3d deposition in human dermal and renal diseases

C3d represents an attractive localization target to bring fH to complement-active tissues due to its covalent link to cell surfaces and associated reported long tissue-residence time.[36,37,39–41] However, while immunostaining using anti-C3c antibody is a common clinical diagnostic of active complement in kidney and skin, C3d deposition is less well characterized.[40] To better understand the relationship between C3c and C3d deposition across indications, we surveyed tissue from a subset of skin and kidney diseases. Anti-C3d and anti-C3c immunofluorescence revealed minimal background staining in healthy human skin (Figures 1A–1C). In contrast, both C3c and C3d immunostaining were detected in a coincident pattern at the dermal-epidermal junction (DEJ) of affected skin in patients diagnosed with discoid lupus erythematosus (DLE) (Figures 1D–1F) or BP (Figures 1G–1I). Semiquantitative scoring of C3d- and C3c-immunostained samples from patients diagnosed with DLE, BP, and pemphigus revealed consistent deposition of both complement fragments across all three skin diseases (Figures 1J and 1K).

In kidney, strong C3d immunofluorescence was present in biopsies from patients with C3G (Figure S1A), consistent with the well-established role for complement in this disease.[42–44] Low C3d immunostaining was detected in samples from patients diagnosed with thrombotic microangiopathy (Figure S1B), while moderate staining was present in samples from patients with anti-neutrophilic cytoplasmic autoantibody (ANCA) vasculitis (Figure S1C) and antibody-mediated rejection of transplanted kidney (Figure S1D). Strong immunostaining was evident in samples from MN (Figure S1E), IgAN (Figure S1F), and both class III and class IV lupus (Figures S1G and S1H). Semiquantitative scoring of anti-C3d immunostaining confirmed generally higher-density C3d deposition in C3G, MN, IgAN, and both classes of lupus nephritis (Figure 1L). These findings are also broadly consistent with similar quantitation of anti-C3 fragment (C3c) (Figure 1M).

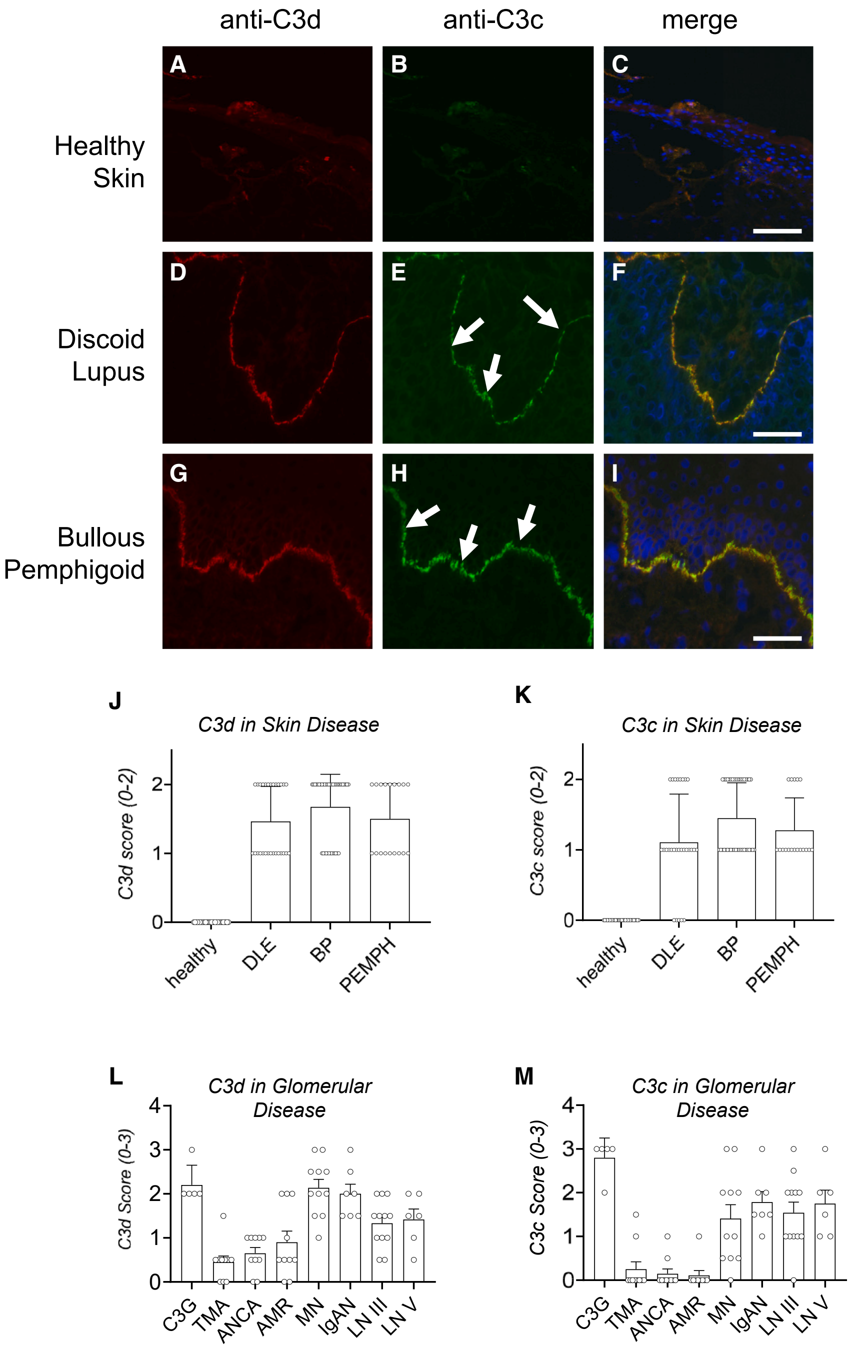

Figure 1. C3d target deposition in human disease

Representative glomerular immunofluorescence in human biopsies from (A–C) healthy skin, (D–F) skin from a patient with discoid lupus erythematosus (DLE), and (G–I) skin from a bullous pemphigoid (BP) patient. Biopsies were stained with anti-C3d antibody (A, D, G) or anti-C3c antibody, which recognizes fragments generated by active complement (B, E, H). White arrows in (E) and (H) indicate the location of the dermal-epidermal junction (DEJ). Co-localization of anti-C3d (red) and C3c (green) immunostaining is shown in (C), (F), and (I), together with DAPI co-staining (blue). (J) and (K) patient show semiquantitative scoring of anti-C3d (J) and anti-C3c (K) in biopsies from healthy skin (n = 30) and from DLE (n = 28), BP (n = 40), and Pemphigus (PEMPH) (n = 18) patients. (L) and (M) show semiquantitative scoring of anti-C3d (L) and anti-C3c (M) in biopsies from patients with C3 glomerulopathy (C3G) (n = 5), thrombotic microangiopathy (TMA) (n = 10), anti-neutrophilic cytoplasmic autoantibody vasculitis (ANCA) (n = 10), antibody-mediated rejection of kidney transplant (AMR) (n = 10), membranous nephropathy (MN) (n = 11), IgA nephropathy (IgAN) (n = 7), and class III and class IV lupus nephritis (n = 12 and n = 6, respectively). Scale bar in (C), 100 μm. Scale bar in (F) and (I), 50 μm.

To further explore the prevalence of complement activity in selected renal diseases, we performed a retrospective analysis of C3 fragment staining in a larger sample cohort (Table S1). Consistent with our prospective analysis, these data revealed a significant number of ANCA patients (43/104) that were C3c fragment positive, while patients with MN (86.2% of samples positive), IgAN (89.7% of samples positive), and lupus nephritis (96.3% of class III and 87.0% of class IV samples positive) indicated a high prevalence of glomerular complement activation in these diseases. Taken together, these data in skin and kidney suggest that C3d deposition is a feature of a subset of autoimmune diseases affecting multiple organs, and that C3d targeting could be a means of locally delivering a complement inhibitor for these indications.

Generation of human and mouse anti-C3d-fH$_{1-5}$ fusion proteins and characterization of binding to C3d

We generated anti-C3d-targeted fH$_{1-5}$ fusion proteins, collectively designated C3d-mAb-2fH, to localize complement inhibition to C3d-positive tissue. Mouse and human C3d-mAb-2fH are approximately 213 kDa recombinant bifunctional fusion proteins consisting of an anti-C3d monoclonal antibody linked to two moieties of the first five SCRs of factor H (fH$_{1-5}$) (Figure 2A). Mouse C3d-mAb-2fH (ADX-118) and the mouse/human chimeric fusion ADX-048 rely on a previously identified monoclonal mouse anti-C3d IgG1 antibody (3d8b) that binds with low-nanomolar affinity to an epitope present in mouse, cynomolgus monkey, and human C3d, iC3b, and C3dg (Table S2).[45,46] As noted earlier, for simplicity we refer to this binding target as "anti-C3d." The 3d8b antibody was subsequently humanized by CDR (complementarity-determining regions) grafting onto a human germline acceptor framework, followed by additional amino acid modifications to reduce potential immunogenicity, improve antibody stability,

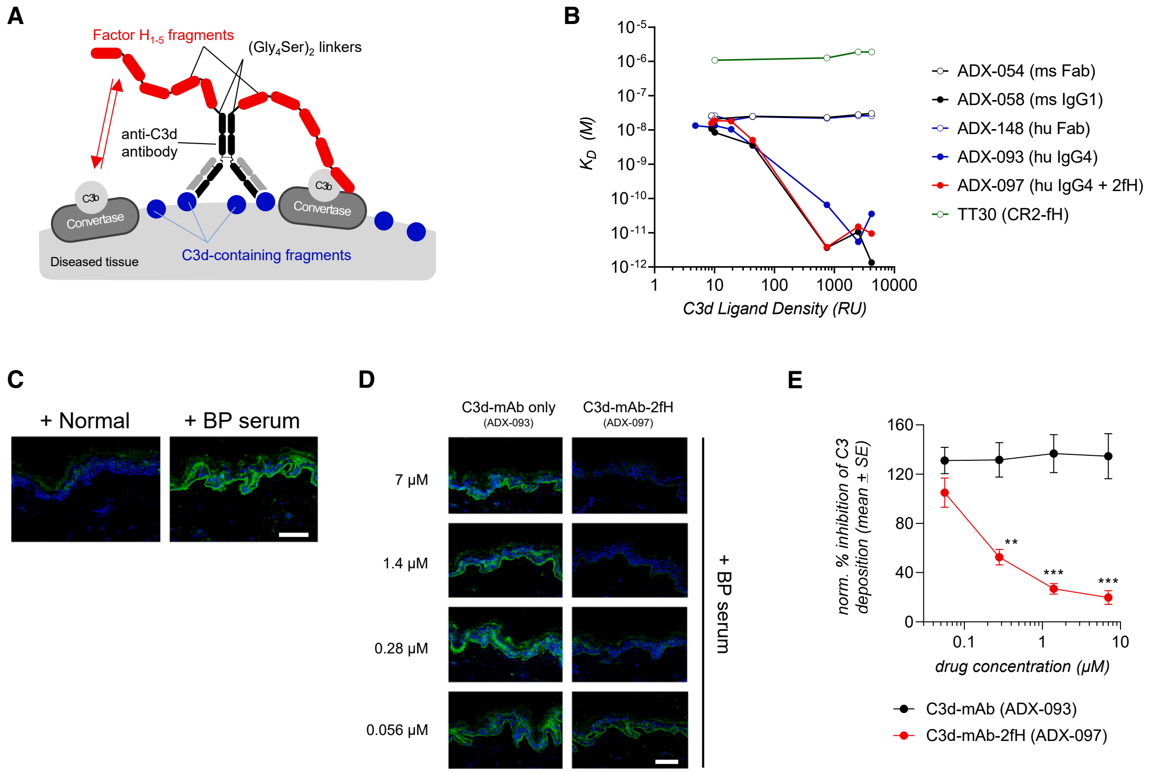

Figure 2. C3d-mAb-2fH design, comparison of binding affinities, and inhibition of complement deposition on human skin

(A) Schematic of the design of C3d-mAb-2fH. (B) Summary of binding affinities measured by surface plasmon resonance (SPR) with increasing density of recombinant C3d. Binding affinity of mouse (ms) (ADX-054) and human (hu) (ADX-148) C3d-binding Fab proteins is roughly 75-fold greater than TT30/CR2-fH at all C3d densities tested. Binding of mouse (ADX-058) and human (ADX093) C3d-targeting monoclonal antibodies (mAbs), or of the human C3d-mAb-2fH fusion protein ADX-097, is similar to anti-C3d Fab binding at low density but increases by >100-fold at higher C3d densities. (C) Deposition of complement fragments (green) after incubation of sections of human skin with serum from bullous pemphigoid (BP) patients, but not after incubation with serum from non-diseased patients. Scale bar, 100 μm. (D) Addition of human C3d-mAb-2fH (ADX-097) fusion protein, but not of the C3d-targeting antibody alone (ADX-093), inhibits deposition on sections of human skin that has been exposed to BP serum. Scale bar, 100 μm. (E) Dose-response curve of the effect of ADX-097 and ADX-093 treatment on complement deposition on human skin sections. ADX-097 significantly inhibits complement deposition in the nanomolar range (**p < 0.0002; ***p < 0.00002).

and minimize potential for antibody-dependent cell-mediated cytotoxicity or complement-dependent cytotoxicity.[46] The resulting human IgG4 antibody, ADX-093, retained similar affinity as 3d8b to human C3d (Table S2) and was therefore used in the human C3d-mAb-2fH fusion protein ADX-097.

The first five SCRs of fH (fH_{1-5}) are both necessary and sufficient to catalyze AP convertase dissociation by accelerating decay of the C3bBb complex and by serving as a co-factor for factor I-mediated cleavage of C3b to iC3b.[34,35] Prior work from our group demonstrated that fusion of CR1 complement regulatory domains to the C termini of targeting antibody heavy chains yielded optimal complement inhibition.[46] Similarly, fH_{1-5} moieties to the antibody heavy-chain C termini via $(Gly_4Ser)_2$ linkers (Figure 2A) did not affect anti-C3d binding, as ADX-118 (mouse antibody/mouse fH_{1-5}) and ADX-097 (human antibody/human fH_{1-5}) bound mouse, cynomolgus monkey, and human C3d with similar affinity as their parent antibodies, 3d8b and ADX-093 (Table S2).

ADX-097 and targeting antibody alone (ADX-093, ADX-058) showed similar binding affinities by surface plasmon resonance (SPR) measured over a range of C3d densities (Figure 2B and Table 2), further indicating that the fH_{1-5} moieties on ADX-097 do not affect C3d binding. Increasing C3d density by 420-fold led to an approximately 2,000-fold increase in binding affinity (K_D) compared to monovalent Fab fragments ADX-148 and ADX-054 (Figure 2B and Table 2). The lower dissociation constants (k_{off}) of the bivalent monoclonal antibodies (mAbs) indicate that this is likely driven by avidity (Table 2). We also compared binding of C3d-mAb-2fH to TT30, which monovalently localizes a single moiety of fH to C3d and related fragments via a domain of the B cell C3d/iC3b receptor CR2 (CD21).[35,47–49] At all C3d densities tested, the K_D of TT30 was in the low-micromolar range, with a rapid off-rate that made it impossible to calculate an accurate k_{off} (Figure 2B and Table 2). This is 50- to 75-fold weaker binding affinity than anti-C3d Fabs at any C3d density and >150,000-fold weaker affinity than ADX-097 at high C3d density. Taken together, these binding data illustrate two important advantages of localizing fH_{1-5} using a bivalent antibody.

Table 1. *In vitro* complement inhibition by C3d-mAb-fH fusion proteins

Protein ID	Anti-C3d antibody	Backbone	Effector	Complement pathway (Wieslab assay)	IC_{50} (nM)
sfH_{1-5}	–	–	1× human fH_{1-5}	alternative	1,980 ± 163
				classical	no activity
ADX-048	3d8b	mouse IgG1	2× human fH_{1-5}	alternative	73 ± 8.3
ADX-093	humanized 3d8b	human IgG4	–	alternative	no activity
ADX-145	–	Fc fusion (IgG4)	2× human fH_{1-5}	alternative	325 ± 61
ADX-097	humanized 3d8b	human IgG4	2× human fH_{1-5}	alternative	81 ± 3.9
				classical	610 ± 93

First, antibodies are capable of high-affinity monovalent anti-C3d binding that is not achieved using fragments of an endogenous C3d-binding protein. Second, a bivalent antibody biases fH_{1-5} targeting toward high-density C3d deposition, potentially favoring localization to dysregulated rather than to homeostatic complement activity.

In vitro characterization of complement inhibition by human and mouse C3d-targeted fH_{1-5}

To understand whether linking fH_{1-5} to an anti-C3d antibody affects its C3 regulatory properties, we assessed the ability of C3d-mAb-2fH to act as a factor I (fI) co-factor, catalyzing fluid-phase C3b cleavage.[50–53] C3b contains two distinct chains, α′ (110 kDa) and β (70 kDa), that dissociate under reducing conditions. Together, fI and fH cleave the α′ chain into smaller protein fragments that can be separated and visualized by SDS-PAGE. Recombinant C3b co-incubated with fI in the presence of anti-C3d antibody (ADX-093) did not cleave C3b (Figure S2A). Combining C3b, fI, and full-length fH (FL fH) resulted in dose-dependent cleavage of the 110-kDa C3α′ chain into 68-, 46-, and 43-kDa fragments (C3α′ −68, −46, and −43, Figure S2B), demonstrating co-factor activity of fH. fH_{1-5} resulted in similar C3b cleavage (Figure S2C), although its catalytic potency was somewhat lower than that of FL fH (Figure S2E). ADX-097 (human C3d-mAb-2fH) also catalyzed fI-mediated C3b cleavage (Figure S2D). The cleavage efficiency of ADX-097 was similar to that of fH_{1-5} (Figures S2E and S2F), indicating that in this purely fluid-phase assay, linking to fH_{1-5} to an antibody did not substantially affect its fI co-factor activity.

To measure complement inhibition more quantitatively, we evaluated the effect of C3d-mAb-2fH fusions on formation of AP- or CP-generated C5b-9 (MAC) in human complement-preserved serum (Wieslab assays). Human fH_{1-5} inhibited AP complement (half-maximal inhibitory concentration [IC_{50}] = 1,980 ± 163 nM) but demonstrated no measurable inhibition of CP complement (Table 1). Fusion of two fH_{1-5} moieties to a human Fc domain (ADX-145) demonstrated a roughly 6-fold increase in AP complement inhibition (IC_{50} = 325 ± 61 nM), suggesting that the presence of a second fH_{1-5} domain may confer an avidity effect. The humanized anti-C3d antibody ADX-093 by itself showed no inhibition of AP complement, but fusion of two fH_{1-5} domains (ADX-097) resulted in a further 4-fold increase in potency vs. ADX-145 (IC_{50} = 81 ± 3.9 nM). ADX-097 retained strong selectivity for AP complement, as inhibition of CP-initiated complement activity remained approximately 10-fold weaker (IC_{50} = 610 ± 83 nM). A chimeric molecule consisting of two moieties of human fH_{1-5} linked to mouse 3d8b (ADX-048) showed AP complement inhibition similar to that of ADX-097 (IC_{50} = 73 ± 8.3 nM), indicating that fusion of fH_{1-5} to either the mouse or human anti-C3d antibodies results in similar potency.

Wieslab AP and CP assays are designed for human serum and do not work well in rodent sera.[54] Therefore, to assess C3d-mAb-2fH potency in mouse and rat, a crucial prerequisite for *in vivo* testing in rodent species, we used an assay that relies on zymosan particles incubated in complement-preserved serum. The human fusion protein ADX-097 showed similar potency in human and mouse serum (IC_{50} = 191 ± 17 nM in human and 202 ± 54 nM in mouse) and was slightly more potent in rat serum (IC_{50} = 99 ± 17 nM) (Table S3). In comparison to ADX-097, the mouse fusion protein ADX-118 was 3- to 4-fold more potent in mouse (IC_{50} = 46 ± 3.9) but 2- to 3-fold less potent in rat serum (281 ± 52 nM). ADX-118 showed no complement inhibition in human serum. Together, these data demonstrate activity of both the mouse and human fusion proteins in rodent, enabling subsequent *in vivo* studies to assess tissue-targeted pharmacokinetics and pharmacodynamics (PK/PD).

Evaluation of C3d-mAb-2fH activity on human skin explants

To test inhibition of complement in the context of human tissue, we evaluated human C3d-mAb-2fH (ADX-097) in a skin explant assay.[55] Cryosections of human skin were pre-incubated with heat-inactivated (complement-inactive) normal human serum or serum from BP patients, allowing pathogenic autoantibodies in the BP serum to bind the skin section. After washing, sections were then incubated with complement-active human serum (with or without inhibitors), and complement deposition was detected by immunofluorescence with an anti-C3c antibody. Sections pre-incubated with normal serum exhibit minimal anti-C3c immunofluorescence, while those pre-incubated with BP serum show substantial C3c signal, indicating C3b tissue deposition (Figure 2C). Addition of the anti-C3d-binding antibody ADX-093 had no effect, while ADX-097 concentrations as low as 0.28 μM significantly inhibited skin C3b deposition ($p < 0.0002$, Figures 2D and 2E), with complete inhibition of complement occurring between 0.28 and 1.4 μM (60–300 μg/mL ADX-097

Table 2. C3d-mAb-2fH binding affinity rises with increased C3d density

		Low C3d density (10 RU)		Low-med C3d density (40 RU)		Med C3d density (750 RU)		Med-high C3d density (2,500 RU)		High C3d density (4,200 RU)	
Protein ID	Anti-C3d antibody	K_D (nM)	k_{off} (1/s)	K_D (nM)	k_{off} (1/s)	K_D (nM)	k_{off} (1/s)	K_D (nM)	k_{off} (1/s)	K_D (nM)	k_{off} (1/s)
ADX-058 (3d8b)	ms IgG1	8.47	9.7×10^{-4}	3.67	3.9×10^{-4}	0.004	4.1×10^{-7}	0.011	1.2×10^{-6}	0.001	1.5×10^{-7}
ADX-054	ms Fab	20.9	1.8×10^{-3}	24.9	3.9×10^{-4}	23.0	1.6×10^{-3}	27.9	1.6×10^{-3}	30.3	1.4×10^{-3}
ADX-093	hu IgG4	13.4	7.8×10^{-4}	3.49	3.9×10^{-4}	0.065	4.9×10^{-6}	0.005	4.4×10^{-7}	0.036	2.9×10^{-6}
ADX-148	hu Fab	26.1	1.4×10^{-3}	24.5	3.9×10^{-4}	21.9	1.5×10^{-3}	25.7	1.4×10^{-3}	25.8	1.3×10^{-3}
ADX-097	hu IgG4 + 2fH	18.8	8.2×10^{-4}	5.06	3.9×10^{-4}	0.004	2.3×10^{-7}	0.015	9.8×10^{-7}	0.010	6.7×10^{-7}
TT30	hu CR2-fH	1,080	ND	NT	NT	1,260	ND	1,890	ND	1,890	ND
**Fold ↑ C3d density**		**1×**		**4×**		**75×**		**250×**		**420×**	

RU, relative density units; ms, mouse; hu, human; fH, factor $H_{1–5}$ fragment; ND, not able to determine; NT, not tested.

in serum). These data indicate that ADX-097 can inhibit human tissue complement deposition, even in the highly dysregulated context of BP serum.

C3d-targeted $fH_{1–5}$ localized to and blocked local tissue complement in *CfH*$^{-/-}$ mice

We evaluated the C3d-mAb-2fH *in vivo* distribution and local complement inhibition in fH knockout mice (*CfH*$^{-/-}$), which have uncontrolled systemic complement activation and increased C3 deposition in the liver and kidney, resulting in sporadic complement-mediated kidney injury.[56] Tissue complement activity in kidney and liver was evaluated by immunofluorescence with an antibody against non-tissue-linked active C3 split products (C3b/iC3b/C3c, which we collectively refer to as "anti-C3 fragment") and for drug localization by staining with anti-human fH (anti-fH). After a single intravenous (i.v.) dose of untargeted human $fH_{1–5}$ (hu $fH_{1–5}$), anti-fH immunostaining indicated no localization to liver or kidney (Figures S3G and S3O), and complement activity in these tissues was unchanged compared to *CfH*$^{-/-}$ + PBS (Figures S3B, S3C, S3J, and S3K). In contrast, significant anti-fH immunostaining was evident in *CfH*$^{-/-}$ mice treated with chimeric C3d-mAb-2fH (ADX-048) (Figures S3H and S3P), demonstrating targeting to tissues with active complement. These mice also showed a marked decrease in both liver and kidney anti-C3 fragment immunofluorescence (compare Figures S3D and S3L to S3C and S3K), indicating that, unlike non-targeted hu $fH_{1–5}$, C3d-mAb-2fH potently inhibits complement activity in tissue for at least a week after dosing.

We next assessed the kinetics of C3d-mAb-2fH-mediated glomerular complement inhibition using a 5 mg/kg i.v. dose of C3d-mAb-2fH, C3d-targeting antibody alone (ADX-093), or vehicle control. C3 fragment immunofluorescence, measured by digital image quantitation, was similar in ADX-093-dosed *CfH*$^{-/-}$ mice and in untreated *CfH*$^{-/-}$ mice (compare black line and shaded area in Figure S3Q). However, dosing with ADX-097 significantly and durably reduced glomerular C3 fragment deposition in *CfH*$^{-/-}$ mice (red line in Figure S3Q). Anti-human IgG4 immunostaining demonstrated accumulation of both the ADX-093 and ADX-097 proteins to glomeruli (Figures S4E and S4F). Detection with both anti-fH and anti-IgG4 show similar tissue distribution (compare Figures S3H and S4F), indicating that the ADX-097 fusion protein remains intact *in vivo*. These data demonstrate that both the human and human/mouse chimeric C3d-mAb-2fH fusion proteins localize to and block tissue convertase activity in *CfH*$^{-/-}$ mice and that inhibition requires the presence of fH moieties.

A detailed dose-ranging time course in *CfH*$^{-/-}$ mice more thoroughly evaluated the relationship between circulating and tissue PK/PD. These studies used mouse C3d-mAb-2fH, ADX-118, to minimize the potential for cross-species anti-drug antibody (ADA) formation that could affect drug exposure. ADX-118 was delivered subcutaneously (s.c.) at doses ranging from 0.3 to 25 mg/kg and i.v. at 5 mg/kg. All doses resulted in glomerular localization of ADX-118 (gray lines in Figures 3A–3E; representative immunofluorescence is shown in Figure S5), with maximal drug concentration in tissue (tissue C_{max}) correlating to drug dose. At all doses, localized drug was detected in tissue for at least 10 days, returning to background levels by 14–17 days post dose. Comparison of 5 mg/kg delivered s.c. vs. i.v. suggests that the i.v. route distributed more rapidly to tissue, translating to greater tissue complement inhibition at the 8-h time point (compare black lines in Figures 3B and 3E; representative immunofluorescence is shown in Figure S6). However, both routes of administration achieved similar tissue C_{max} and, beyond the first 24 h after dosing, both inhibited complement similarly. While tissue drug exposure was dose correlated, 1, 5, or 25 mg/kg s.c. ADX-118 doses led to similar maximal complement inhibition, although lower doses required more time to reach maximal inhibition (black lines in Figures 3A–3C). Importantly, maximum tissue complement inhibition in the 1 mg/kg and 5 mg/kg s.c. groups was reached prior to tissue C_{max} (Figures 3B, 3C, and S6), suggesting that anti-C3d target saturation is not required to fully inhibit tissue AP complement. The 0.3 mg/kg dose group inhibited complement for several days but did not reach the same maximum achieved by the 1, 5, and 25 mg/kg doses (Figure 3D). Finally, 1 week after dosing, the 1, 5, and 25 mg/kg groups all retained maximal complement inhibition, indicating that ADX-118-mediated tissue complement inhibition is quite durable.

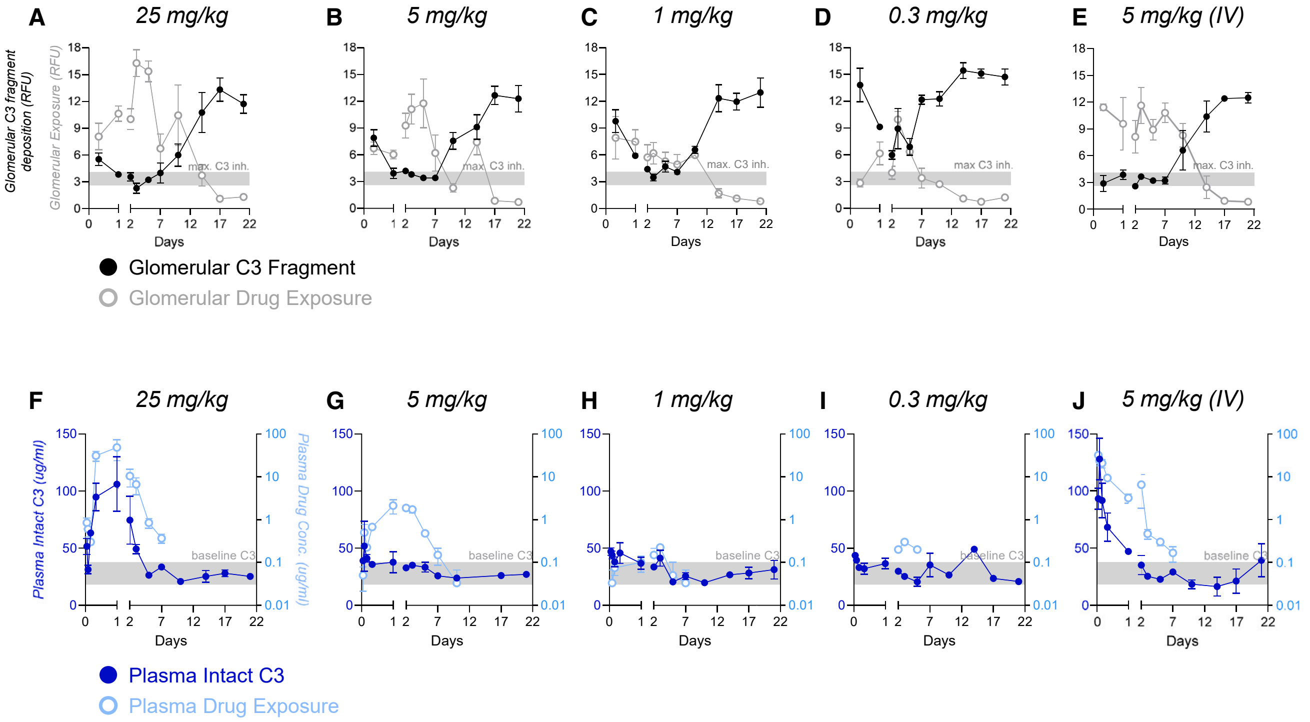

Figure 3. Glomerular and circulating pharmacokinetics and pharmacodynamics of C3d-mAb-2fH

CfH$^{-/-}$ mice received a single dose of mouse C3d-mAb-2fH (ADX-118) at the indicated concentrations. Dosing route was s.c. unless otherwise indicated. Kidney tissue was collected at 8 h and 1, 2, 3, 5, 7, 10, 14, 17, and 22 days after dosing (n = 4 mice per time point). Plasma was collected at 1, 2, 4, and 8 h, and 1, 2, 3, 5, 7, 10, 14, 17, and 22 days after dosing. (A–E) Summary of image quantitation data for C3 fragment deposition (black lines) and tissue drug levels (anti-fH, gray lines). ADX-118 levels were dose dependent (compare gray lines in A–D) and showed measurable drug levels in kidney for at least 2 weeks after dosing. Tissue C_{max} of 5 mg/kg s.c. (B) and i.v. (E) were similar. The level of maximum C3 fragment inhibition was defined as the average C3 fragment level in the 25 mg/kg group at 1, 2, 3, and 5 days ± 1 SD (A) and is indicated as a gray bar in (A)–(E). Peak inhibition of C3 fragment deposition was similar in the 25 mg/kg (A), 5 mg/kg (B, E), and 1 mg/kg (C) groups, while partial inhibition is achieved in the 0.3 mg/kg group (D). Measurement of circulating drug exposure (light-blue lines) and circulating intact C3 (dark-blue lines) is shown in (F)–(J). Circulating ADX-118 levels were dose dependent (compare light-blue lines in F–I). Circulating C_{max} was approximately 15-fold higher for i.v. vs. s.c. delivery (5 mg/kg) (compare G and J). Circulating intact C3 levels transiently increased in the 25 mg/kg s.c. (F) and 5 mg/kg i.v. (J) groups, as these groups achieved sufficient circulating drug concentration to transiently slow C3 consumption. However, despite inhibiting complement in glomeruli, s.c. doses ≤5 mg/kg did not attain sufficiently high circulating C3d-mAb-2fH concentrations to affect systemic C3 cleavage (G–I).

Local complement can be inhibited by C3d-mAb-2fH at doses that do not affect circulating complement

Because uncontrolled AP complement activity in *CfH*$^{-/-}$ mice consumes C3 faster than new C3 protein is generated, these mice exhibit a 10- to 20-fold reduction in circulating intact C3 protein levels.[56] *In vivo* administration of an exogenous AP complement inhibitor temporarily reduces this consumption, leading to a transient increase in intact plasma C3 that can serve as a sensitive biomarker of systemic complement inhibition.[57–59] Twenty-four hours after delivery, a single 25 mg/kg s.c. dose of ADX-118 reached a circulating C_{max} of approximately 50 μg/mL (light blue in Figure 3F), corresponding to a measurable increase in intact plasma C3 that returned to baseline as drug cleared from circulation (dark blue in Figure 3F). I.v. delivery of 5 mg/kg ADX-118 also resulted in sufficient drug exposure (∼30 μg/mL) to elicit transient elevation of intact plasma C3 (Figure 3J). In contrast, s.c. delivery of ≤5 mg/kg ADX-118 led to lower C_{max} and overall exposure levels, resulting in negligible plasma C3 elevation (Figures 3G–3I). Thus, while s.c. doses of 1–5 mg/kg are capable of potent and durable local inhibition in tissue (Figures 3B and 3C), they do not achieve sufficiently high plasma drug concentrations to affect circulating complement in *CfH*$^{-/-}$ mice.

C3d-mAb-2fH localized to active complement in primate skin

We next examined whether human C3d-mAb-2fH (ADX-097) can target local complement in primates. High-dose UVB irradiation activates epidermal complement,[60,61] and we therefore developed a model of UVB-induced skin complement activation in cynomolgus monkeys. Erythema was induced in monkey skin by transient exposure to UVB light using a hand-held lamp (Figures S7A–S7D). A time course of skin biopsies was collected after exposure and immunostained using anti-C3c and anti-C3d antibodies (Figures S7E–S7H and S7I–S7L). Co-localized deposition of complement fragments was

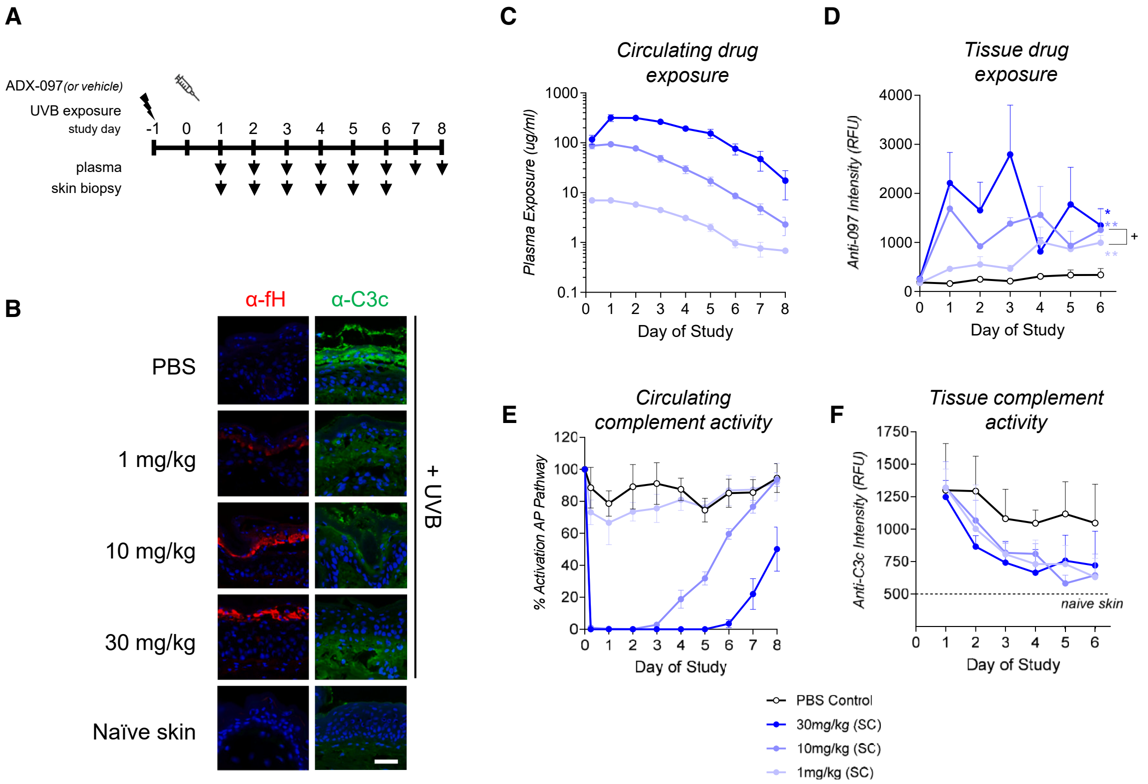

Figure 4. C3d-mAb-2fH localizes to skin in cynomolgus monkeys

(A) Study design to test distribution and complement inhibition in UVB-induced cynomolgus monkey skin. Discrete sections of shaved skin were exposed to 3,900 mJ/cm^2 UVB irradiation on day −1 of the study. ADX-097 or PBS vehicle control was delivered systemically by s.c. injection. Skin biopsies from the irradiated areas and plasma were collected daily for 6 days after dosing. (B) Representative images from sections collected 72 h after ADX-097 dosing. Sections were stained with an anti-fH antibody (red) to detect localization of fH_{1-5} in skin or with an antibody that recognizes C3c fragments (green) to detect areas of active complement. All sections were counterstained with DAPI (blue). Scale bar, 50 μm. (C) Plasma exposure (μg/mL) of ADX-097, measured by ELISA, over time. (D) Tissue exposure of ADX-097, measured by anti-fH immunofluorescence and digitally quantified, over time demonstrate drug localization to complement-active epidermis. Area under the curve (AUC) of anti-fH immunostaining for all time points is statistically different vs. PBS control for all ADX-097 dosed groups (*p < 0.02, **p < 0.005). AUC of the 10 mg/kg ADX-097 group is greater than that of the 1 mg/kg dosed monkeys ($^+p < 0.05$). Tissue exposure (AUC) of the 30 mg/kg group is not statistically different from the 10 mg/kg group (p = 0.26) but is statistically different from the 1 mg/kg group (p < 0.04). (E) Systemic complement inhibition was measured by Wieslab assay in monkey serum. Thirty mg/kg and 10 mg/kg ADX-097 inhibited complement in serum in a dose-dependent manner (p < 0.0001, $\%Activity_{AUC}$ between the two doses). A non-statistically significant trend toward inhibition was observed between the $\%Activity_{AUC}$ of the PBS control and 1 mg/kg ADX-097 groups (p = 0.07), suggesting the possibility of minimal systemic inhibition at early time points after ADX-097 dosing. (F) Tissue complement inhibition was measured by anti-C3c immunostaining and image quantitation. All doses of ADX-097 tested resulted in a non-statistically significant trend (p = 0.11–0.16) toward reduced tissue complement compared to PBS-treated controls.

observed in the epidermis as early as 24 h after exposure and lasted for at least 72 h (Figures S7M–S7P).

To evaluate localization of C3d-mAb-2fH to primate skin, UVB injury was induced on study day −1, 24 h prior to drug dosing (Figure 4A). Complement activation, marked by anti-C3c immunostaining, was observed after UVB exposure in vehicle (PBS)-treated monkeys (compare PBS to naive skin, Figures 4B and S8). Human C3d-mAb-2fH was administered systemically by s.c. injection on day 0, and drug localization was detected as early as 24 h after dosing (Figure S8). Total tissue drug exposure, measured as area under the curve (AUC), was dose correlated and was greater than the PBS control for all doses tested (p < 0.02) (Figures 4B–4D and S8). Notably, while ADX-097 localizes to tissue in the 1 mg/kg dose group, no inhibition of systemic complement was observed in plasma collected from this group (Figure 4E). In the 10 mg/kg and 30 mg/kg dose groups, inhibition of circulating complement waned as circulating drug levels dropped below approximately 70 μg/mL (Figures 4C and 4E). Circulating drug concentration in the 1 mg/kg group remained below 10 μg/mL throughout the study (Figure 4C). All tested doses of ADX-097 showed similar tissue complement inhibition, reaching maximal inhibition roughly 3 days after s.c. dosing (Figure 4F). However, this difference was not statistically significant (p = 0.11–0.15), a likely consequence of the small number of samples (n = 3) collected at each time point. Nevertheless, these data are consistent with those observed in $CfH^{-/-}$ mice, suggesting that in addition to homing to skin complement, ADX-097 may locally regulate complement in non-human primate (NHP) skin.

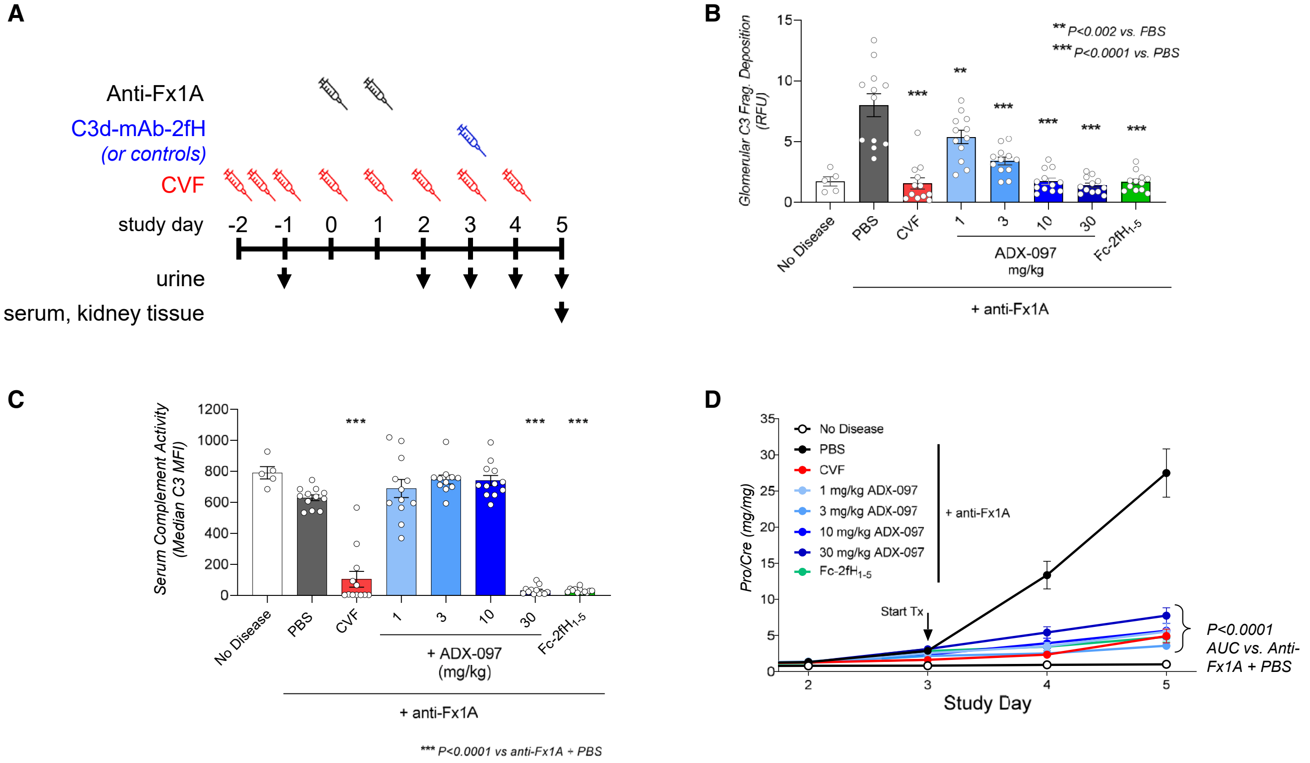

Figure 5. C3d-mAb-2fH reduces proteinuria and inhibits glomerular complement without affecting systemic complement

(A) Study design in the passive Heymann nephritis (PHN) model of kidney injury. (B) Glomerular C3 fragment deposition was quantified from anti-C3 fragment immunofluorescence on kidney tissue. Compared to non-disease controls, C3 fragment deposition is elevated in PHN rats treated with PBS ($p < 0.0001$). CVF treatment reduces anti-C3 fragment to levels equivalent to non-disease controls ($p < 0.0001$). ADX-097 treatment reduces glomerular C3 fragment deposition in a dose-dependent manner: 1 mg/kg ADX-097 reduces anti-C3 fragment immunofluorescence by approximately 40% ($p < 0.002$), and 3 mg/kg reduces it by approximately 70% ($p < 0.0001$). ADX-097 doses $\geq$10 mg/kg, as well as 17 mg/kg Fc-2fH$_{1–5}$, reduced anti-C3 fragment immunofluorescence to levels equivalent to non-diseased controls. (C) Compared to healthy controls, serum complement activity was slightly but not statistically significantly reduced in PHN rats treated with PBS. Activity was significantly inhibited in PHN rats treated with CVF, 50 mg/kg ADX-097, or 17 mg/kg Fc-2fH$_{1–5}$ (all $p < 0.0001$ vs. PHN + PBS). In contrast, compared to PHN + PBS, ADX-097 doses $\leq$10 mg/kg did not affect circulating complement. (D) Urine protein/creatinine ratio (uPCR) was elevated by day 3 in rats treated with anti-Fx1A nephrotoxic serum alone and continued to increase until the end of the study. Treatment with cobra venom factor (CVF), dosed daily beginning 2 days prior to anti-Fx1A instillation, significantly inhibited uPCR ($p < 0.0001$). Single i.v. doses of 1–30 mg/kg human C3d-mAb-2fH (ADX-097) inhibited uPCR progression ($p < 0.0001$). No dose response was observed, suggesting that 1 mg/kg ADX-097 achieves maximal efficacy in this time frame. A 17 mg/kg dose (equimolar to 30 mg/kg ADX-097) of a non-targeted inhibitor, Fc-2fH$_{1–5}$, also inhibited uPCR progression.

Intravenous C3d-targeted fH$_{1–5}$ reduced renal injury in a rat model of membranous nephropathy

We tested disease-modifying efficacy of C3d-mAb-2fH in the PHN model of membranous nephropathy in rats. PHN is induced by administering a sheep serum raised against a preparation of rat proximal tubules (anti-Fx1A).[62,63] Anti-Fx1A causes subepithelial immune deposits in the glomerular basement membrane (GBM), leading to pathologic changes in the GBM and podocytes that are reflected as elevated urine protein levels.[62] Initial renal injury in PHN is driven by complement activation, as treatment with cobra venom factor (CVF), a C3 analog that depletes endogenous complement, or with a small-molecule factor B inhibitor, effectively reduces disease in this model.[64–67]

Because C3d-mAb-2fH targeting occurs through C3d binding, we first assessed the time course of C3d deposition after anti-Fx1A treatment (Figure S9). Consistent with the role of complement in PHN, glomerular anti-C3d immunostaining was first detected in kidneys 2 days after delivery of anti-Fx1A (Figures S9A and S9B) while proteinuria, measured as urine protein/creatinine ratio (uPCR), appeared by day 3 ($p < 0.001$ vs. healthy control, Figure 5D). Based on these data, C3d-mAb-2fH was dosed on day 3 to evaluate therapeutic efficacy after disease onset.

We initially tested the human C3d-mAb-2fH, ADX-097, administered i.v. after onset of proteinuria (day 3 after disease induction) at doses ranging from 1 to 30 mg/kg. As a comparator, a separate cohort of rats was dosed daily with 100 U/kg CVF beginning 2 days prior to disease induction (for study design see Figure 5A). We used the human drug candidate ADX-097 in these studies because the short drug-treatment period (2–4 days) minimizes concerns that cross-species ADAs could affect drug exposure. Glomerular and systemic

complement activity was assessed in samples collected 2 days after ADX-097 administration (day 5 after disease induction). Representative images from both anti-C3 fragment and anti-fH immunofluorescence are shown in Figure S10. In PHN kidney tissue, ADX-097 treatment led to dose-dependent reduction of glomerular complement activity (image quantitation of anti-C3 fragment immunostaining) (Figure 5B). Notably, while ADX-097 doses $\geq$10 mg/kg completely inhibited activity, the 1 mg/kg and 3 mg/kg dose groups showed approximately 40% and 75% reduction in C3 fragment deposition, respectively. Non-targeted Fc-2fH$_{1-5}$ at a molar-equivalent dose to 30 mg/kg ADX-097 also inhibited glomerular C3 fragment deposition (Figure 5B). However, while both the 30 mg/kg ADX-097 and Fc-2fH$_{1-5}$ groups had similar concentrations in circulation (Figure S11A), only ADX-097 localizes to glomeruli (Figure S11B), suggesting that Fc-fH$_{1-5}$ acts through fluid-phase/circulating rather than local complement inhibition. Consistent with this hypothesis, both Fc-2fH$_{1-5}$ and 30 mg/kg ADX-097 also inhibited serum complement (Figure 5C). In contrast, no systemic complement inhibition was detected in samples from animals treated with 1, 3, or 10 mg/kg ADX-097 (Figure 5C), indicating that C3d targeting drives the potency of ADX-097's effects on complement-mediated disease at these lower doses.

All doses of ADX-097 reduced the progression of proteinuria (uPCR) as early as 24 h after injection and to a similar degree as prophylactic CVF treatment (Figure 5B). This was not due to diminished anti-Fx1A localization, as equivalent sheep IgG deposition was detected in ADX-097-treated and control PHN glomeruli collected 5 days after disease induction (2 days after ADX-097 dosing) (Figure S12). No correlation between ADX-097 dose and reduced uPCR progression was observed, suggesting that the minimal efficacious dose of ADX-097 is below the lowest tested dose (1 mg/kg). We note that the 1 mg/kg and 3 mg/kg doses yielded 40% and 75% decreases in glomerular anti-C3 fragment immunofluorescence, respectively, but their effect on proteinuria was comparable to fully blocking anti-C3 fragment immunostaining (Figures 5B and 5D). These data suggest that partial complement inhibition may be sufficient for disease-modifying efficacy in this model.

Soluble C5b-9 in urine correlated with tissue complement inhibition

The C5b-9 protein complex is an end product of complement activation, leading to formation of pores that disrupt pathogen and target cell membranes, driving cell lysis and death.[68] Tissue C5b-9 deposition is detected in a wide spectrum of kidney diseases, including membranous, IgA, hypertensive, and diabetic nephropathies, as well as lupus nephritis, thrombotic microangiopathies, and C3 glomerulopathy.[69] Soluble C5b-9 is also detected in urine (uC5b-9) from PHN rats and from patients suffering from IgAN, membranous nephropathy, and pre-eclampsia, suggesting that uC5b-9 may also reflect tissue complement activity in the kidney.[70–73] Because ADX-097 inhibits in tissue without affecting circulating complement, our PHN studies provide an opportunity to further evaluate uC5b-9 as an indicator of renal complement. In urine samples collected from PHN rats on study day 5, 48 h after treatment with PBS or with 1, 3, or 10 mg/kg ADX-097 (see Figure 5A), uC5b-9 concentration normalized to urine creatinine (uC5b-9/uCre) was reduced by ADX-097 in a dose-dependent manner (Figure S13A). Notably, all ADX-097-treated rats showed similar reductions in proteinuria (Figure 5D), suggesting that changes in uC5b-9/uCre do not simply reflect decreased urine protein. Furthermore, we found a highly significant correlation (Spearman's r = 0.76; $p < 0.00000001$) between uC5b-9/uCre and tissue glomerular C3 fragment deposition, measured by anti-C3 fragment immunostaining (Figure S13B), suggesting that uC5b-9/uCre is a urine biomarker of kidney tissue complement activation.

Low-dose, subcutaneous C3d-mAb-2fH reduced renal injury in PHN rats

In a separate study, we further explored ADX-097 potency in the PHN model using lower and s.c. doses of ADX-097 (0.3, 1, and 3 mg/kg) to understand the minimum efficacious dose (for study design see Figure 6A). We also included s.c. doses of Fc-2fH$_{1-5}$ at molar equivalence to ADX-097 (0.17, 0.51, and 1.7 mg/kg, matching 0.3, 1, and 3 mg/kg ADX-097, respectively) to more directly compare C3d targeted vs. non-targeted complement inhibition.

In tissue collected 4 days after dosing (7 days after disease induction), we observed a statistically significant reduction in glomerular anti-C3 fragment immunofluorescence in rats dosed with 1 mg/kg or 3 mg/kg ADX-097 (Figure 6B). Treatment with equimolar doses of Fc-2fH$_{1-5}$, however, showed no equivalent effect. Anti-fH immunostaining was detected in ADX-097-treated glomeruli but not in those that received Fc-2fH$_{1-5}$ (Figure 6C), confirming a lack of fH$_{1-5}$ localization in the absence of the C3d-targeting antibody.

Forty-eight hours after s.c. injection (5 days after disease induction), 1 mg/kg and 3 mg/kg doses of ADX-097 reduced uPCR relative to anti-Fx1A + PBS controls ($p < 0.0007$), while the 0.3 mg/kg ADX-097 group exhibited a non-statistically significant trend ($p = 0.07$) toward proteinuria reduction (Figure 6D). By 96 h after injection, all ADX-097 treatment groups exhibited a statistically significant reduction in proteinuria ($p < 0.005$) that correlated with ADX-097 dose (Figure 6D). A similar correlation was evident when uPCR was analyzed as AUC ($uPCR_{AUC}$): Compared to anti-Fx1A + PBS, 3 mg/kg ADX-097 reduced $uPCR_{AUC}$ by 73% ± 8.7% ($p < 0.003$), and 1 mg/kg (s.c.) reduced $uPCR_{AUC}$ by 59% ± 10% ($p < 0.01$) (Figure 6E). $uPCR_{AUC}$ reduction in the 0.3 mg/kg group (33% ± 22%) was not statistically significant, with the larger error reflecting a more variable response at this low dose. Efficacy of 3 mg/kg ADX-097 was similar to that of prophylactic CVF treatment (uPCR reduced by 76% ± 11%). We note, however, that despite daily dosing with CVF, the reduction of uPCR in this group waned between study days 5 and 6. This may be due to CVF's high propensity to induce a neutralizing ADA response that can reduce circulating exposure.[74–76] This interpretation is further supported by concomitant lack of glomerular complement inhibition in tissue (Figure 6B) and by urine C5b-9/Cre, which was similar to that of non-diseased control rats until study day 5 but elevated by day 7 (Figure 6F).

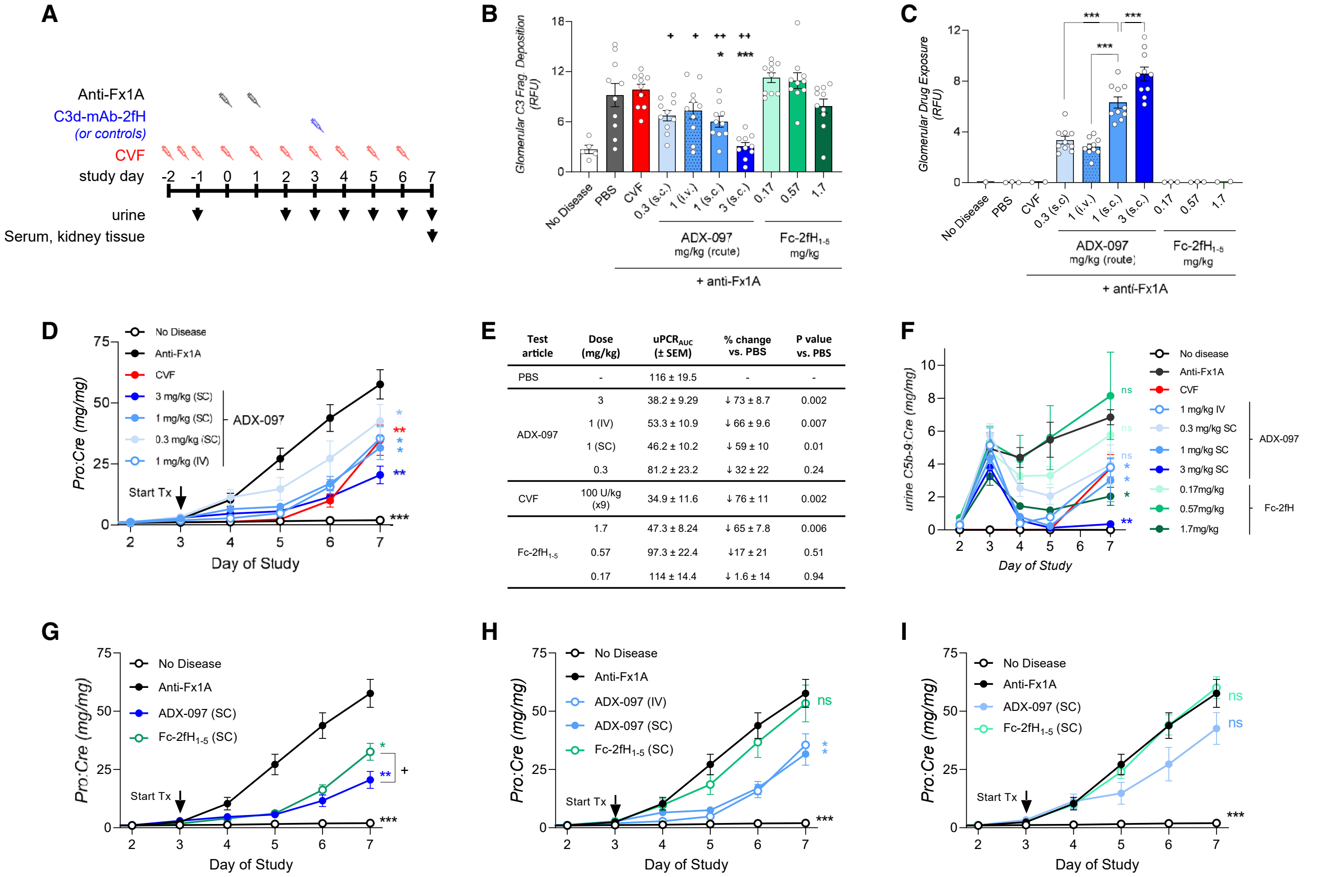

Test article	Dose (mg/kg)	$uPCR_{AUC}$ (± SEM)	% change vs. PBS	P value vs. PBS
PBS	-	116 ± 19.5	-	-
ADX-097	3	38.2 ± 9.29	↓ 73 ± 8.7	0.002
	1 (IV)	53.3 ± 10.9	↓ 66 ± 9.6	0.007
	1 (SC)	46.2 ± 10.2	↓ 59 ± 10	0.01
	0.3	81.2 ± 23.2	↓ 32 ± 22	0.24
CVF	100 U/kg (x9)	34.9 ± 11.6	↓ 76 ± 11	0.002
$Fc\text{-}2fH_{1\text{-}5}$	1.7	47.3 ± 8.24	↓ 65 ± 7.8	0.006
	0.57	97.3 ± 22.4	↓17 ± 21	0.51
	0.17	114 ± 14.4	↓ 1.6 ± 14	0.94

Figure 6. C3d targeting of fH dose-dependently reduces renal injury in passive Heymann nephritis

(A) Summary of study design in the passive Heymann nephritis (PHN) model of kidney injury. PHN rats were treated with CVF starting on study day −2 (prior to disease induction by anti-Fx1A) or after onset of proteinuria on study day 3 with human C3d-mAb-2fH (ADX-097) at doses of 0.3, 1, or 3 mg/kg s.c. or with 1 mg/kg i.v. $Fc\text{-}2fH_{1–5}$ was included in the study at s.c. doses equimolar to ADX-097. (B) Glomerular C3 fragment deposition (quantitation of anti-C3 fragment immunofluorescence) in glomeruli from PHN rats. C3 fragment deposition is significantly increased in anti-Fx1A-induced PHN kidneys ($p < 0.0001$). CVF-treated PHN rats do not exhibit reduced anti-C3 fragment signal, a likely consequence of CVF clearance due to immunogenicity. ADX-097-treated PHN rats show a dose-dependent inhibition of glomerular C3 fragment deposition, which is reduced to levels equivalent to non-diseased controls in the 3 mg/kg s.c. group and is partially reduced in the 1 mg/kg s.c. group (*$p < 0.05$, ***$p < 0.0001$ vs. anti-Fx1A + PBS). None of the $Fc\text{-}2fH_{1–5}$-treated dose groups show a statistically significant difference in glomerular C3 fragment deposition compared to the PBS-treated PHN control group. All C3d-mAb-2fH-treated groups show a statistically significant reduction in C3 fragment deposition compared to their molar-equivalent dose of Fc-2fH ($^{+}p < 0.05$, $^{++}p < 0.005$ vs. equivalent-dose $Fc\text{-}fH_{1–5}$). (C) ADX-097 localization to glomeruli was detected by quantitation of anti-fH immunofluorescence. No ADX-097 is observed in healthy controls or in PHN rats dosed with PBS or CVF. Importantly, glomerular fH localization is also not detected in rats that received any dose of $Fc\text{-}2fH_{1–5}$. In contrast, anti-fH immunofluorescence is measured at all doses of ADX-097. Staining is dose dependent across groups receiving s.c. ADX-097. However, i.v. dosing results in less glomerular localization that an equivalent dose delivered s.c. (compare 1 mg/kg s.c. vs. i.v. dosing groups), suggesting that the duration rather than the concentration of the C_{max} is a key determinant of ADX-097 accumulation in tissue (***$p < 0.0001$). (D) On study day 5, ADX-097 treatment at doses ≥ 1 mg/kg showed similar reduction in proteinuria compared to CVF. At this time point, the 0.3 mg/kg group showed reduced uPCR, though less substantial than CVF or higher doses of ADX-097. At the end of the study (day 7), all ADX-097-treated groups exhibited a dose-dependent reduction in proteinuria (*$p < 0.01$, **$p < 0.005$, ***$p < 0.0001$ vs. anti-Fx1A + PBS). (E) Summary of $uPCR_{AUC}$ for days 3–7 of study. (F) Time course of urine C5b-9/Cre ratio (uC5b-9) suggests that 1 mg/kg and 3 mg/kg doses of ADX-097 equivalently inhibit glomerular complement in the first 48 h after dosing but that effects on uC5b-9 are more durable in the 3 mg/kg dose group. $AUC_{uC5b\text{-}9}$ of 1 and 3 mg/kg ADX-097 is reduced compared to the untreated anti-Fx1A dose group ($p < 0.03$ for 1 mg/kg s.c. and i.v.; $p < 0.002$ for 3 mg/kg s.c.). $AUC_{uC5b\text{-}9}$ of the 0.3 mg/kg ADX-097 dose group and of the 0.17 and 0.57 mg/kg Fc-2fH dose groups are not statistically significantly different from that of untreated anti-Fx1A, indicating little effect of these treatments on uC5b-9. $AUC_{uC5b\text{-}9}$ of the 1.7 mg/kg Fc-2fH dose group is reduced relative to untreated anti-Fx1A ($p < 0.008$), likely reflecting the longer circulating half-life of Fc-2fH. (G) Comparison of 3 mg/kg ADX-097 and the molar-equivalent dose of $Fc\text{-}2fH_{1–5}$ (1.7 mg/kg). Both molecules reduce uPCR equivalently up to day 5, but by day 7 the effect of ADX-097 is more potent than that of $Fc\text{-}2fH_{1–5}$ ($^{+}p < 0.05$). (H) Comparison of 1 mg/kg s.c. or i.v. ADX-097 to 0.57 mg/kg s.c. $Fc\text{-}2fH_{1–5}$. Both dosing routes of ADX-097 significantly reduce uPCR and $uPCR_{AUC}$ (*$p < 0.01$), while $Fc\text{-}2fH_{1–5}$ has no statistically significant effect on either means of assessing proteinuria. (I) Comparison of 0.3 mg/kg s.c. ADX-097 and 0.17 mg/kg s.c. $Fc\text{-}2fH_{1–5}$. $Fc\text{-}2fH_{1–5}$ has no effect on proteinuria. ADX-097 shows a non-statistically significant trend toward proteinuria reduction.

Local complement inhibition preserved podocyte architecture

A subset of renal cortex samples collected on study day 7 was further analyzed by transmission electron microscopy (TEM) to visualize ultrastructural changes in the glomeruli. Figures S14A and S14B show representative TEM images from a healthy control glomerulus, with a GBM of uniform thickness and displaying a distinct lamina densa. Healthy podocytes with normal foot-process morphology and well-differentiated slit diaphragms resided along the length of the GBM (white arrows in Figures S14A and S14B). In contrast, representative glomeruli from rats treated with anti-Fx1A exhibited extensive podocyte foot-process effacement (yellow arrows in Figures S14C and S14D), with rare slit-diaphragm-like structures evident along a distorted GBM of varying thickness. Electron-dense subepithelial deposits were visible between some podocytes and the underlying GBM (yellow asterisks in Figure S14D), consistent with prior observations in the PHN model and representing accumulation of immune complexes at the filtration barrier.[62] Figures S14E and S14F show representative images from a PHN rat treated with 3 mg/kg ADX-097. ADX-097 rescued podocyte architecture along a substantial portion of the GBM. While local examples of effaced podocytes (yellow arrows in Figures S14E and S14F) and electron-dense deposits (yellow asterisk in Figure S14F) were observed, the number of well-differentiated podocyte foot processes (white arrows in Figures S14E and S14F) was clearly increased throughout multiple evaluated samples. These data further demonstrate preservation of glomerular ultrastructure after ADX-097 treatment consistent with dose-dependent attenuation of uPCR.

C3d-mediated drug targeting improved potency compared to non-targeted $fH_{1–5}$

To further understand the relationship between potency and tissue targeting, we compared efficacy of ADX-097 to that of equimolar doses of Fc-2f$H_{1–5}$. Forty-eight hours after s.c. delivery, 3 mg/kg ADX-097 and 1.7 mg/kg Fc-2f$H_{1–5}$ equivalently reduced uPCR relative to controls, suggesting that both proteins initially inhibit renal injury. Fc-2f$H_{1–5}$ exhibited 22- to 43-fold higher circulating exposure at end of the study compared to equimolar doses of ADX-097 (Figure S15B), a finding consistent with prior studies suggesting that C3d binding shortens the circulating half-life of anti-C3d antibodies and implying greater Fc-2f$H_{1–5}$ systemic exposure across the course of the study.[77] If the circulating C_{max} of both ADX-097 and Fc-2f$H_{1–5}$ was high enough to inhibit circulating complement in this model, this effect would be prolonged in the 1.7 mg/kg Fc-2f$H_{1–5}$ group, potentially explaining its proteinuria reduction. We note, however, that both 3 mg/kg and 1 mg/kg doses of ADX-097 inhibit urine C5b-9 more potently than 1.7 mg/kg Fc-2f$H_{1–5}$ (Figure 6F), indicating that C3d targeting increased the potency of $fH_{1–5}$ beyond any transient effects on fluid-phase/circulating complement. This also translated to greater durability as, 96 h after dosing, proteinuria in the 3 mg/kg ADX-097 group was less than in rats treated with 1.7 mg/kg Fc-2f$H_{1–5}$ ($p < 0.05$) (Figure 6G). Thus, while complicated by potential effects on circulating complement, 3 mg/kg ADX-097 appears to be more potent than an equimolar dose of untargeted $fH_{1–5}$.

Comparison of the 1 mg/kg ADX-097 (s.c. and i.v.) and 0.57 mg/kg Fc-2f$H_{1–5}$ treatment groups revealed a more clear-cut efficacy difference, as ADX-097 but not Fc-2f$H_{1–5}$ reduced uPCR and $uPCR_{AUC}$ (Figure 6H). Notably, s.c. and i.v. routes show similar degrees of inhibition, indicating that circulating C_{max}, which would be higher in the i.v. dosed group, had little effect on ADX-097 efficacy. A similar difference was observed between the 0.3 mg/kg ADX-097 and 0.17 mg/kg Fc-2f$H_{1–5}$ treatment groups, although the difference between these low-dose groups is not statistically significant (Figure 6I).

Urine-soluble uC5b-9/Cre ratio further elucidated both the potency difference between C3d-mAb-2fH/ADX-097 and Fc-2f$H_{1–5}$ (Figure 6F) and the kinetics of inhibition. Prior to dosing on day 3, uC5b-9/Cre was elevated in anti-Fx1A-treated rats ($p < 0.02$), and untreated and drug-treated dose groups showed no statistically significant difference in uC5b-9/Cre. In the first 48 h after dosing, 1 mg/kg and 3 mg/kg doses of ADX-097 equivalently inhibited glomerular complement, but effects on uC5b-9/Cre were more durable in the 3 mg/kg dose group, showing continued inhibition at the end of the study on day 7. AUC_{uC5b-9} of 1 mg/kg and 3 mg/kg ADX-097 was reduced relative to the untreated anti-Fx1A dose group ($p < 0.03$ for 1 mg/kg s.c. and i.v.; $p < 0.002$ for 3 mg/kg s.c.). AUC_{uC5b-9} of the 0.3 mg/kg ADX-097 dose group and of the 0.17 mg/kg and 0.57 mg/kg Fc-2fH dose groups were not statistically significantly different from that of untreated anti-Fx1A, indicating minimal effect of these treatments on uC5b-9. AUC_{uC5b-9} of the 1.7 mg/kg Fc-2fH dose group was reduced relative to untreated anti-Fx1A ($p < 0.008$), likely reflecting the longer circulating half-life of Fc-2fH as discussed above (Figure S15B). Thus, urinary C5b-9/Cre further underscores the potency advantage conferred by anti-C3d targeting of the $fH_{1–5}$ moieties. Together with reduced proteinuria and potent inhibition of tissue complement deposition, these data demonstrate that ADX-097/C3d-mAb-2fH is a potent, durable, and local inhibitor of complement activation and effector generation at low doses, suggesting potential for therapeutic use in complement-mediated diseases.

DISCUSSION

Therapeutics that target systemic complement are constrained by complement's essential role in innate immunity and by the fact that complement components exist in high abundance and undergo rapid systemic turnover.[15–17,21,22] As a result, therapies relying on this strategy must contend with large pharmacologic sinks while striking a balance between safety and potency. Consequently a substantial unmet need remains, particularly in autoimmune diseases where standard of care includes immunosuppressive agents.[15,16] Targeting drug to tissues where complement is dysregulated has the potential to improve potency by avoiding circulating sinks while minimizing systemic complement blockade, leaving complement-dependent homeostatic functions relatively intact. The data presented here suggest that localizing $fH_{1–5}$ to tissue-deposited C3d is an effective strategy for targeted complement inhibition, resulting in potent and durable local complement inhibition at doses that do not affect systemic complement.

Translational studies presented here indicate that high-density C3d deposition is a feature of complement dysregulation across a range of tissues. We observed C3d immunostaining in both skin and kidney disease, including DLE, BP, MN, IgAN, and lupus nephritis (Figures 1 and S1). We note that C3d deposition co-localized with active complement (anti-C3c immunofluorescence), consistent with the fact that C3d is linked to complement activity rather than being tissue or organ specific. Thus, C3d targeting may have translational applications across a wide range of complement-driven diseases. While not the focus of this work, further analysis of associations between the density of C3d deposition and disease severity will be of interest. These findings support C3d as a localization target to bring a therapeutic to tissues with active complement. In addition, they suggest a translational strategy for C3d-mAb-2fH that focuses on indications with strong C3d deposition and/or patients for whom local C3d immunostaining is a part of their clinical diagnosis.

C3d-mAb-2fHs are human, mouse, and mouse/human chimeric antibody fusion proteins that direct fH_{1-5} to tissue-deposited C3d, thereby inhibiting local AP complement. We demonstrate in multiple models and species that these fusions localize to and regulate tissue complement sites of high C3d density, resulting in potent and durable local complement re-regulation at doses that do not affect systemic complement. In SPR studies, bivalent anti-C3d antibodies bound high-density C3d with approximately 2,000-fold greater affinity than monovalent Fabs (Figure 2B and Table 2), illustrating the significant avidity advantage of using a bivalent targeting antibody. Concordantly, even at low doses, C3d-mAb-2fH appeared to have a longer tissue-residence time than tissue-targeting strategies that rely on monovalent binding to their localization target.[48,51,59] Furthermore, in addition to providing drug localization and durability, this avidity may also confer a binding preference toward high-density tissue C3d over lower-density C3d that is present on circulating blood cells.[78–80]

We also found that saturation of the C3d-binding target does not appear to be required for maximum local complement inhibition. In both mouse and NHP models, C3d-mAb-2fH was distributed to skin in a dose-dependent manner (Figures 3D, 4A–4C, and 4E) while effects on complement activation showed more limited dose dependence (Figures 3F, 4A–4C, and 4E). We believe this reflects the mechanism of C3d-mAb-2fH, in which affinity for high-density C3d durably holds the fH_{1-5} fragments in the vicinity of active complement. Because fH-mediated complement regulation is catalytic—a single molecule of C3d-mAb-fH can dissociate and degrade multiple convertase complexes—this long tissue-residence time greatly amplifies fH_{1-5}-driven convertase decay and degradation. Furthermore, fH-induced degradation also leaves behind tissue-bound C3d, raising the possibility that C3d-mAb-2fH tissue localization may enhance its own convertase regulatory function either while the drug remains in circulation or upon subsequent dosing. These attributes combine to yield localized drug potency and durability that may be difficult to achieve through competitive or allosteric AP inhibition.

Importantly, our data suggest that localized inhibition is sufficient to control complement-driven disease. In the rat PHN model of renal injury, a single ≥ 1 mg/kg dose of human C3d-mAb-2fH (ADX-097) reduced the uPCR (Figure 5D). Accordingly, all doses of ADX-097 localized to glomeruli and significantly inhibited glomerular complement activity (anti-C3 fragment immunofluorescence) (Figures 5C, S11, and S12B). We also note that 1 mg/kg and 3 mg/kg doses yielded 40% and 75% decreases in glomerular anti-C3 fragment immunofluorescence, respectively, while their effect on proteinuria was comparable to fully blocking anti-C3 fragment immunostaining (Figure 5D). These data suggest that complete local complement inhibition may not be required to inhibit proteinuria. We cannot rule out, however, that some of the residual staining could represent inactive forms of C3, particularly iC3b.[48,59] Generation of more selective C3 fragment antibodies may be required to address this question.

While all tested doses of ADX-097 inhibited glomerular complement, doses ≤ 10 mg/kg left serum activity intact by 48 h after dosing (Figures 5C and 5D), indicating that tissue-targeted complement inhibition is achievable with limited effect on serum complement. An additional study showed that 1 mg/kg ADX-097 (i.v. or s.c.) significantly reduced proteinuria and urine C5b-9, while an equimolar dose (0.57 mg/mL) of Fc-$2fH_{1-5}$ had no effect (Figures 6E and 6I). ADX-097 at 3 mg/kg had a greater effect on urine C5b-9 and a more durable effect on proteinuria than equimolar Fc-$2fH_{1-5}$ (1.7 mg/kg) (Figure 6F). Importantly, due to limitations on *in vivo* blood collection in the PHN model, we cannot rule out that circulating concentrations of ADX-097 may have transiently affected fluid-phase inhibition in these studies. However, an extensive PK/PD time course showed that 1 mg/kg and 5 mg/kg s.c. doses of C3d-mAb-2fH inhibit tissue complement in $CfH^{-/-}$ mice without affecting intact C3 levels in plasma, a biomarker of circulating complement, even when plasma drug is at C_{max} (Figure 3). These data demonstrate a clear potency advantage of tissue targeting and furthermore suggest that local inhibition is sufficient for controlling complement-mediated disease.

Finally, treatment-induced changes in soluble urine C5b-9 in the PHN model provide important translational insight into this biomarker. Tissue deposition of C5b-9 is present in lupus nephritis, thrombotic microangiopathies, C3 glomerulopathy, and membranous, IgA, hypertensive, and diabetic nephropathies.[69] Soluble C5b-9 has also been reported in urine (uC5b-9) in the rat PHN model and from patients suffering from IgAN, MN, and pre-eclampsia, suggesting that uC5b-9 may reflect tissue complement activity in the kidney.[70–73] However, because serum-soluble C5b-9 is also elevated in disease,[81–83] it has not been clear whether changes in urine C5b-9 stem from changes in the circulating biomarker or from intrinsic changes in tissue complement activation. We show here that urine-soluble C5b-9 tightly correlates with tissue-specific complement inhibition (Figure S13), suggesting that the bulk of urine C5b-9 in the PHN model stems directly from renal tissue rather than from changes in filtration of circulating soluble C5b-9. While additional studies in

patients and in other model systems is warranted, this finding suggests that measurement of urine C5b-9 could be eventually be used in place of biopsy collection and immunostaining to track changes in tissue complement deposition.

Taken together, the data presented here describe a novel approach to local targeting of a complement-blocking therapy. Translational data confirm that high-density C3d deposition is a common feature of local complement activation across multiple tissue types, suggesting a therapeutic homing target as well as an opportunity for selecting patients who might most benefit from a C3d-directed inhibitor. Based on this, we designed mouse, human, and chimeric antibody fusion proteins that target fH_{1-5} to tissue complement via a high-affinity anti-C3d antibody (C3d-mAb-2fH). We show that this molecule efficiently localizes fH_{1-5} in human tissue *ex vivo* and *in vivo* in mouse, rat, and NHP models of complement activation. These data also elucidate key features of the C3d-mAb-2fH mechanism, suggesting that both antibody avidity and the catalytic mechanism of fH contribute to the potency and durability of the molecule. Critically, our data indicate that local complement inhibition is sufficient to alter disease progression. We also show that soluble urine C5b-9 (uC5b-9) closely reflects renal tissue complement inhibition in this model, suggesting uC5b-9 as a potential biomarker of local kidney complement activity. Collectively, these studies support further evaluation of the human C3d-mAb-2fH fusion protein ADX-097 in the clinic, with the potential for potent, durable, and targeted complement inhibition for a range of complement-driven diseases.

MATERIALS AND METHODS

Sourcing, immunostaining, and semiquantitative scoring of human samples

A retrospective study of human skin biopsies was carried out using formalin-fixed paraffin-embedded (FFPE) samples obtained from the Mass General Brigham Biobank (Boston, MA). After deparaffinization, antigen retrieval was accomplished using DAKO Target Retrieval Solution combined with heat treatment in a BioCare Medical Pressure Cooker. Slides were then blocked in a solution of 10% goat serum, 1% BSA, and 0.05% Na-azide in 1× PBS, followed by immunostaining for anti-C3d (clone 3d8b, mouse IgG_1 kappa), followed by detection with an AF647-conjugated anti-mouse secondary or with a fluorescein isothiocyanate (FITC)-conjugated anti-C3c (DAKO F0201, rabbit IgG). Skin immunostaining was scored by a trained pathologist on a scale of 0–2 (negative staining was assigned as "0"). Thirty samples from healthy biopsies and a minimum of 18 samples for each disease state were included in this analysis.

Frozen human kidney biopsy tissue blocks were supplied by Arkana Laboratories (Little Rock, AR). Kidney tissue cryosections were stained with anti-C3d mAb (clone 3d8b, mouse IgG_1 kappa) followed by a secondary FITC-conjugated goat anti-mouse IgG Fc. Fluorescence intensity of glomerular C3d staining was scored in blinded fashion by a pathologist for each case using a scale of 0–3+ (negative staining was assigned as "0" and trace staining as "0.5" in the plotted graphs). A subset of the human kidney samples used in this study was stained for C3 deposition using an FITC-conjugated goat anti-human C3 polyclonal antibody (#B1C/B1A; Kent Labs, Bellingham, WA). The fluorescence intensity of glomerular C3 staining was scored in blinded fashion by a pathologist for each case using a scale of 0–3+ (negative staining was assigned as "0" and trace staining as "0.5" in the graphs). A retrospective analysis was conducted based on the historical C3 scores to determine the frequency of C3-positive staining.

Characterization of C3d binding

Binding affinity measurements were performed on Biacore 3000 or T200 at 25°C. Flow cells of the CM5 chip were coated with low surface density of human, cynomolgus monkey, or mouse C3d using a standard EDC/NHS amine coupling method in sodium acetate (pH 5.0). Anti-C3d antibodies and fusion proteins were diluted in buffer in a concentration series ranging between 0 and 200 nM (0–1,000 nM for CR2-fH_{1-5}, due to its weaker binding affinity) and injected over surface-bound target antigen at a flow rate of 30 μL/min for 120 s followed by dissociation in running buffer for 180 s. Binding of antibody fusion proteins was monitored in real time and fit using a Langmuir (1:1) binding model. From the observed k_{on} and k_{off}, K_D was determined. Surfaces were regenerated using two injections of glycine at pH 1.7 for 40 s.

In vitro measurement of complement inhibition

Wieslab assays

Complement activation assays were performed using the Wieslab AP and CP ELISAs (SVAR Life Science, Malmö, Sweden) according to manufacturer's instructions.

Classical pathway hemolysis (CH_{50}) assay

Sheep red blood cells (SRBCs) were washed in gelatin veronal buffer with $MgCl_2/CaCl_2$ (GVB^{++}) and isolated by centrifugation. Pelleted SRBCs were washed in GVB^{++}, then sensitized with rabbit anti-sheep erythrocyte antiserum for 30 min at 30°C.[84] Complement inhibition was assessed as decreased SRBC lysis vs. untreated controls.

Complement deposition on human skin explants

Complement activation by BP immune complexes was performed as previously described.[55] Compounds or a solvent were added with the addition of the complement source (diluted in normal human plasma). Slides were examined by fluorescence microscopy and image analysis in blinded fashion. Results are based on n = 10 per group, whereby the donor skin and patient IgG were varied.

UVB-induced skin complement activation model in non-human primates

NHP UVB skin model was performed at Biomere (Worcester, MA). Twenty-four naive cynomolgus monkeys of 2–5 years of age were enrolled. Animals were sedated with Telazol, and UVB exposure at 3,120 mJ/cm^2 was applied to dorsal skin on day −1 using a SolRx 100-series Ultraviolet Phototherapy Lamp Unit (SolarcSystems, ON, Canada). A piece of UV light blocking film with a center hole of 2-cm diameter was attached to the lamp unit surface to block all the UV light except the center 2-cm diameter area, so that only an

area of the 2-cm circle was exposed to UVB at each skin testing site. Each animal received s.c. administration of ADX-097 or vehicle on day 0. Blood collections and skin biopsies were collected at specified time points (n = 3 per time point) (Figure 4A). Skin biopsies from naive animals were collected at the time of the final, group-specific time point. All animals were returned to the testing facility's colony at the completion of post-biopsy medication and observations.

NHP circulating PK/PD assays

Plasma PK analysis was conducted using an ADX-097-specific ELISA. Plates were coated with an anti-ideotype antibody that recognizes ADX-097 (Q32Bio CL00027) for total drug capture. Detection was accomplished using a biotinylated mouse anti-human factor H (Thermo Fisher [Waltham, MA] MA5-17735, clone OX-24) followed by a standard streptavidin HRP A/TMB colorimetric assay. Plasma complement activity assays were performed at Q32Bio using the Wieslab AP Kit (SVAR Life Science) according to manufacturer's instructions. AP pathway activation was determined by normalizing values at each time point to pre-dose control.

NHP skin immunostaining and image analysis

Frozen skin sections (5 μm) were stained after fixation with cold acetone at −20°C. Slides were rinsed with Dulbecco's PBS and loaded onto a Leica BOND Rx autostainer. ADX-097 tissue drug level was detected using a biotinylated mouse anti-human factor H primary antibody (Thermo Fisher MA5-17735, clone OX-24) followed by detection with Alexa 647-conjugated streptavidin (Thermo Fisher S-21374). Slides were co-stained for complement fragment C3c using an FITC- rabbit anti-human C3c (DAKO F0201) antibody. Stained slides were mounted with VectaShield Vibrance anti-fade mounting medium containing DAPI (VectorLab H-1800). Whole slide images were acquired using DAPI, FITC, and Cy5 channels with identical exposure times in each channel across all slides. VisioPharm software was used to identify tissue edges and tissue-free areas to define a region of interest (ROI) corresponding to the epidermis. C3c and C3d signal in this ROI was quantified as an average signal intensity in the appropriate fluorescence channel.

CfH$^{-/-}$ mice

C57BL/6 fH-deficient (*CfH*$^{-/-}$) mice carrying a targeted disruption of the gene encoding fH+ were generously provided by Prof. Matthew Pickering.[56] Mice were housed and studies conducted at Istituto di Ricerche Farmacologiche Mario Negri IRCCS (Italy) or at Biomere (Worcester, MA) according to internal institutional guidelines.

Rodent tissue immunostaining

Mouse or rat tissue samples (n = 5 per time point) were frozen in OCT. Cryosections (5 μm) were fixed in −20°C acetone. Rodent C3 fragments were detected using an FITC-conjugated goat anti-mouse C3 fragment polyclonal antibody (MPBiomedicals, 0855510) or an FITC-conjugated goat anti-rat C3 fragment polyclonal antibody (MPBiomedicals, 0855751). Rodent C3d was stained using a human anti-C3d IgG4 (clone 3d8b, ADX-086) followed by a secondary Alexa Fluor 647-conjugated mouse anti-human IgG4 pFc′ (Southern Biotech 9190-31 clone HP6023) or Alexa Fluor 488-conjugated mouse anti-human IgG4 pFc′ (Southern Biotech 9190-30 clone HP6023). Humanized C3d-mAb-2fH (ADX-097) was detected using FITC-conjugated OX-24 (Thermo Fisher MA5-17736). Mouse C3d-mAb-2fH (ADX-118) was detected using an FITC-conjugated mouse anti-mouse fH1-4 monoclonal antibody (clone 2A5, a gift from Dr. Claire Harris). Immunofluorescence quantitation was performed in blinded fashion using an EVOS M5000 imaging system (Thermo Fisher) and ImageJ software. A minimum of ten glomeruli were assessed per section.

Measurement of plasma C3 in *CfH*$^{-/-}$ mice

Mouse blood was collected by cardiac puncture in the presence of EDTA, chilled on ice, and plasma separated by centrifugation at 2,000 × *g* at 4°C within 15 min of collection. Mouse C3 was detected using a mouse C3 ELISA kit (Genway Biotech, San Diego, CA, GWB-7555C7) following the manufacturer's instructions.

Passive Heymann nephritis model of membranous nephropathy

PHN studies were performed at Inotiv Westminster (formerly Plato BioPharma). Six-week old male Sprague-Dawley rats (n = 10 per group, except for "no disease" control, which were n = 5 per group) were obtained from Charles River Laboratories and allowed to acclimatize for 5 days prior to study initiation. Rats were housed in metabolic cages throughout the study, starting at day 3. Nephritis was induced by administration of two doses of a sheep anti-Fx1A antibody (Dr. David Salant, Boston University School of Medicine, Boston, MA) delivered i.v. at 100 and 300 mg/kg on days 0 and 1 of the study, respectively. Healthy control animals were dosed i.v. with normal sheep serum (Millipore, Burlington, MA). The positive control group was treated intraperitoneally on day −1 with 150 U/kg CVF (Quidel, San Diego, CA), then 100 U/kg daily from day 0 until the end of the study. All other study animals were treated intraperitoneally with PBS on the same schedule. Test proteins ADX-097 and Fc-2fH$_{1-5}$ or PBS were delivered either s.c. or i.v. on day 3. Urine was collected from day 2 to day 7, and body weights and other physiological parameters were assessed daily. At study termination, serum and plasma were collected and both kidneys collected for histology and immunofluorescence. Immunostaining and quantitation were performed as described above for mouse tissue. Urinary protein and creatinine were measured on an Olympus AU400e Clinical Chemistry Analyzer (Beckman Coulter, Brea, CA) using clinical chemistry reagents Micro-Total Protein and creatinine (Sekisui Diagnostics, Burlington, MA). Urinary albumin was measured by ELISA using a rat albumin-specific ELISA kit (Nephrat; Ethos Biosciences, Logan Township, NJ).

Measurement of complement activity in rat serum

Blood was clotted in serum separator tubes, centrifuged, and stored at −80°C. Complement activity was measured using a modified zymosan assay protocol. Ten microliters of serum was mixed with 30 μL of PBS + 0.1% BSA buffer, after which 8.3% pre-activated zymosan (Complement Technologies, Tyler, TX), 16.6 mM EGTA,

and 8.3 mM $MgCl_2$ in 0.1% BSA/PBS was added to all wells (final volume, 100 μL). After incubation, the complement reaction was quenched with 20 μL of 50 mM EDTA and goat anti-rat C3-FITC (MP Biomedicals, Solon, OH) was added. Following washing, the pellets were resuspended in PBS (pH 7.4) and 0.1% BSA. Median fluorescence intensity values were measured to determine complement activity in the serum using an Attune flow cytometer (Thermo Fisher) collecting 10,000 events per well in autosampler mode, and were analyzed in FlowJo (FlowJo, Ashland, OR).

Measurement of soluble C5b-9 in rat urine

Soluble C5b-9 was measured using a Hycult Terminal Complement Complex (HK-106) assay according to the manufacturer's instructions, with the exception that measurement standard was diluted in diluent plus 25% male Sprague-Dawley urine (BioIVT #RAT00URINE0104496, Lot #RAT515719).

Statistics

Statistical significance was determined by one-way ANOVA unless otherwise specified. All statistical calculations were made using embedded functions in GraphPad Prism software.

Study approval

All animal study protocols were approved by and performed under guidelines of the institutional animal care and use committee of the institution where the studies were performed. Approval for collection and use of human samples was as follows. Collection of immune cells and skin samples for skin explant studies was approved by the Ethics Committee, University of Lübeck Retrospective (protocol numbers 09-140 and 04-061). Analysis of human kidney samples was performed under IRB 2017/06/28 (Arkana Laboratories, Little Rock, AR). Human skin specimens were retrieved from the archives of the Department of Pathology at Brigham and Women's Hospital, and the study was conducted with approval of the Institutional Review Board of Brigham and Women's Hospital, Harvard Medical School (2020P003508).

DATA AND CODE AVAILABILITY

All data shown in this work are available from the authors upon request.

SUPPLEMENTAL INFORMATION

Supplemental information can be found online at https://doi.org/10.1016/j.ymthe.2024.02.001.

ACKNOWLEDGMENTS

The work described was funded by Q32 Bio. The authors would like to thank Applied Pathology Systems (Shrewsbury, MA), Biomere (Worcester, MA), and Plato BioPharma/Inotiv (Westminster, CO) for technical support with *in vivo* studies.

AUTHOR CONTRIBUTIONS

K.C.F., M.J.S., S.L.K., J.M.T., V.M.H., and S.M.V. conceived and designed fusion proteins. F.L., S.T.R., K.C.F., J.G.M., M.J.S., A.B., G.R., S.L.K., S.M.V., and S.W. designed research studies. F.L., S.T.R., K.C.F., J.G.M., A.E.C., H.A.C., S.X., L.F.S.-J., K.B., E.S., and M.L. conducted experiments. F.L., S.T.R., K.C.F., J.G.M., A.E.C., A.B., J.M.H., C.G.L., A.V., R.J.L., D.J.S., S.L.K., J.M.T., V.M.H., S.M.V., and S.W. contributed to analysis and interpretation of data. J.M.H., A.B., D.J.S., J.M.T., and V.M.H. provided reagents and specialized technical support. F.L., S.T.R., and S.W. wrote the manuscript. All authors contributed to reviewing the manuscript. Listing order of co-first authors (F.L. and S.T.R.) is alphabetical by surname.

DECLARATION OF INTERESTS

This work was funded by Q32 Bio, Inc., which holds patents on ADX-097 and related molecules. Q32 Bio is currently conducting clinical trials using ADX-097. F.L., S.T.R., K.C.F., J.G.M., A.E.C., S.M.V., and S.W. are current employees and equity holders of Q32 Bio, Inc. M.J.S. and S.L.K. were employees of Q32 Bio, Inc. at the time of their contributions to the described work. J.M.T., and V.M.H. are co-founders of Q32 Bio, Inc. and retain equity in the company.

REFERENCES

1. Stoermer, K.A., and Morrison, T.E. (2011). Complement and viral pathogenesis. Virology *411*, 362–373. https://doi.org/10.1016/j.virol.2010.12.045.
2. Ricklin, D., and Lambris, J.D. (2013). Complement in immune and inflammatory disorders: therapeutic interventions. J. Immunol. *190*, 3839–3847. https://doi.org/10.4049/jimmunol.1203200.
3. Reis, E.S., Mastellos, D.C., Hajishengallis, G., and Lambris, J.D. (2019). New insights into the immune functions of complement. Nat. Rev. Immunol. *19*, 503–516. https://doi.org/10.1038/s41577-019-0168-x.
4. Gros, P., Milder, F.J., and Janssen, B.J.C. (2008). Complement driven by conformational changes. Nat. Rev. Immunol. *8*, 48–58. https://doi.org/10.1038/nri2231.
5. Rodríguez de Córdoba, S., Harris, C.L., Morgan, B.P., and Llorca, O. (2011). Lessons from functional and structural analyses of disease-associated genetic variants in the complement alternative pathway. Biochim. Biophys. Acta *1812*, 12–22. https://doi.org/10.1016/j.bbadis.2010.09.002.
6. Thurman, J.M., and Holers, V.M. (2006). The central role of the alternative complement pathway in human disease. J. Immunol. *176*, 1305–1310. https://doi.org/10.4049/jimmunol.176.3.1305.
7. Łukawska, E., Polcyn-Adamczak, M., and Niemir, Z.I. (2018). The role of the alternative pathway of complement activation in glomerular diseases. Clin. Exp. Med. *18*, 297–318. https://doi.org/10.1007/s10238-018-0491-8.
8. Haddad, G., Lorenzen, J.M., Ma, H., de Haan, N., Seeger, H., Zaghrini, C., Brandt, S., Kölling, M., Wegmann, U., Kiss, B., et al. (2021). Altered glycosylation of IgG4 promotes lectin complement pathway activation in anti-PLA2R1-associated membranous nephropathy. J. Clin. Invest. *131*, e140453. https://doi.org/10.1172/JCI140453.
9. Ricklin, D., Mastellos, D.C., Reis, E.S., and Lambris, J.D. (2018). The renaissance of complement therapeutics. Nat. Rev. Nephrol. *14*, 26–47. https://doi.org/10.1038/nrneph.2017.156.
10. Holers, V.M. (2014). Complement and its receptors: new insights into human disease. Annu. Rev. Immunol. *32*, 433–459. https://doi.org/10.1146/annurev-immunol-032713-120154.
11. Harris, C.L., Pouw, R.B., Kavanagh, D., Sun, R., and Ricklin, D. (2018). Developments in anti-complement therapy; from disease to clinical trial. Mol. Immunol. *102*, 89–119. https://doi.org/10.1016/j.molimm.2018.06.008.
12. Andrighetto, S., Leventhal, J., Zaza, G., and Cravedi, P. (2019). Complement and Complement Targeting Therapies in Glomerular Diseases. Int. J. Mol. Sci. *20*, 6336. https://doi.org/10.3390/ijms20246336.
13. Kaartinen, K., Safa, A., Kotha, S., Ratti, G., and Meri, S. (2019). Complement dysregulation in glomerulonephritis. Semin. Immunol. *45*, 101331. https://doi.org/10.1016/j.smim.2019.101331.

14. Ricklin, D., Mastellos, D.C., and Lambris, J.D. (2019). Therapeutic targeting of the complement system. Nat. Rev. Drug Discov. https://doi.org/10.1038/s41573-019-0055-y.

15. Konar, M., and Granoff, D.M. (2017). Eculizumab treatment and impaired opsonophagocytic killing of meningococci by whole blood from immunized adults. Blood *130*, 891–899. https://doi.org/10.1182/blood-2017-05-781450.

16. McNamara, L.A., Topaz, N., Wang, X., Hariri, S., Fox, L., and MacNeil, J.R. (2017). High Risk for Invasive Meningococcal Disease Among Patients Receiving Eculizumab (Soliris) Despite Receipt of Meningococcal Vaccine. MMWR. Morb. Mortal. Wkly. Rep. *66*, 734–737. https://doi.org/10.15585/mmwr.mm6627e1.

17. Langereis, J.D., van den Broek, B., Franssen, S., Joosten, I., Blijlevens, N.M.A., de Jonge, M.I., and Langemeijer, S. (2020). Eculizumab impairs Neisseria meningitidis serogroup B killing in whole blood despite 4CMenB vaccination of PNH patients. Blood Adv. *4*, 3615–3620. https://doi.org/10.1182/bloodadvances.2020002497.

18. McKeage, K. (2019). Ravulizumab: First Global Approval. Drugs *79*, 347–352. https://doi.org/10.1007/s40265-019-01068-2.

19. Petrosyan, A., Cravedi, P., Villani, V., Angeletti, A., Manrique, J., Renieri, A., De Filippo, R.E., Perin, L., and Da Sacco, S. (2019). A glomerulus-on-a-chip to recapitulate the human glomerular filtration barrier. Nat. Commun. *10*, 3656. https://doi.org/10.1038/s41467-019-11577-z.

20. Gao, S., Cui, Z., and Zhao, M.H. (2022). Complement C3a and C3a Receptor Activation Mediates Podocyte Injuries in the Mechanism of Primary Membranous Nephropathy. J. Am. Soc. Nephrol. *33*, 1742–1756. https://doi.org/10.1681/ASN.2021101384.

21. Sissons, J.G., Liebowitch, J., Amos, N., and Peters, D.K. (1977). Metabolism of the fifth component of complement, and its relation to metabolism of the third component, in patients with complement activation. J. Clin. Invest. *59*, 704–715. https://doi.org/10.1172/JCI108689.

22. Rother, R.P., Rollins, S.A., Mojcik, C.F., Brodsky, R.A., and Bell, L. (2007). Discovery and development of the complement inhibitor eculizumab for the treatment of paroxysmal nocturnal hemoglobinuria. Nat. Biotechnol. *25*, 1256–1264. https://doi.org/10.1038/nbt1344.

23. Fishman, J., Kuranz, S., Yeh, M.M., Brzozowski, K., and Chen, H. (2023). Changes in Hematologic Lab Measures Observed in Patients with Paroxysmal Nocturnal Hemoglobinuria Treated with C5 Inhibitors, Ravulizumab and Eculizumab: Real-World Evidence from a US Based EMR Network. Hematol. Rep. *15*, 266–282. https://doi.org/10.3390/hematolrep15020027.

24. Hayes, W., Tschumi, S., Ling, S.C., Feber, J., Kirschfink, M., and Licht, C. (2015). Eculizumab hepatotoxicity in pediatric aHUS. Pediatr. Nephrol. *30*, 775–781. https://doi.org/10.1007/s00467-014-2990-5.

25. Oruc, A., Ayar, Y., Vuruskan, B.A., Yildiz, A., Aktas, N., Yavuz, M., Gullulu, M., Dilek, K., and Ersoy, A. (2018). Hepatotoxicity associated with eculizumab in a patient with atypical hemolytic uremic syndrome. Nefrologia (Engl Ed) *38*, 448–450. https://doi.org/10.1016/j.nefro.2017.10.001.

26. Coyle, D., Cheung, M.C., and Evans, G.A. (2014). Opportunity cost of funding drugs for rare diseases: the cost-effectiveness of eculizumab in paroxysmal nocturnal hemoglobinuria. Med. Decis. Making *34*, 1016–1029. https://doi.org/10.1177/0272989X14539731.

27. Hoy, S.M. (2021). Pegcetacoplan: First Approval. Drugs *81*, 1423–1430. https://doi.org/10.1007/s40265-021-01560-8.

28. Patriquin, C.J., and Kuo, K.H.M. (2019). Eculizumab and Beyond: The Past, Present, and Future of Complement Therapeutics. Transfus. Med. Rev. *33*, 256–265. https://doi.org/10.1016/j.tmrv.2019.09.004.

29. Kareem, S., Jacob, A., Mathew, J., Quigg, R.J., and Alexander, J.J. (2023). Complement: Functions, location and implications. Immunology *170*, 180–192. https://doi.org/10.1111/imm.13663.

30. Petr, V., and Thurman, J.M. (2023). The role of complement in kidney disease. Nat. Rev. Nephrol. *19*, 771–787. https://doi.org/10.1038/s41581-023-00766-1.

31. Ferreira, V.P., Pangburn, M.K., and Cortés, C. (2010). Complement control protein factor H: the good, the bad, and the inadequate. Mol. Immunol. *47*, 2187–2197. https://doi.org/10.1016/j.molimm.2010.05.007.

32. Parente, R., Clark, S.J., Inforzato, A., and Day, A.J. (2017). Complement factor H in host defense and immune evasion. Cell. Mol. Life Sci. *74*, 1605–1624. https://doi.org/10.1007/s00018-016-2418-4.

33. Makou, E., Herbert, A.P., and Barlow, P.N. (2013). Functional anatomy of complement factor H. Biochemistry *52*, 3949–3962. https://doi.org/10.1021/bi4003452.

34. Gordon, D.L., Kaufman, R.M., Blackmore, T.K., Kwong, J., and Lublin, D.M. (1995). Identification of complement regulatory domains in human factor H. J. Immunol. *155*, 348–356.

35. Fridkis-Hareli, M., Storek, M., Mazsaroff, I., Risitano, A.M., Lundberg, A.S., Horvath, C.J., and Holers, V.M. (2011). Design and development of TT30, a novel C3d-targeted C3/C5 convertase inhibitor for treatment of human complement alternative pathway-mediated diseases. Blood *118*, 4705–4713. https://doi.org/10.1182/blood-2011-06-359646.

36. Magro, C.M., and Dyrsen, M.E. (2008). The use of C3d and C4d immunohistochemistry on formalin-fixed tissue as a diagnostic adjunct in the assessment of inflammatory skin disease. J. Am. Acad. Dermatol. *59*, 822–833. https://doi.org/10.1016/j.jaad.2008.06.022.

37. Pfaltz, K., Mertz, K., Rose, C., Scheidegger, P., Pfaltz, M., and Kempf, W. (2010). C3d immunohistochemistry on formalin-fixed tissue is a valuable tool in the diagnosis of bullous pemphigoid of the skin. J. Cutan. Pathol. *37*, 654–658. https://doi.org/10.1111/j.1600-0560.2009.01450.x.

38. Villacorta, J., Diaz-Crespo, F., Acevedo, M., Guerrero, C., Campos-Martin, Y., García-Díaz, E., Mollejo, M., and Fernandez-Juarez, G. (2016). Glomerular C3d as a novel prognostic marker for renal vasculitis. Hum. Pathol. *56*, 31–39. https://doi.org/10.1016/j.humpath.2016.05.015.

39. Snijders, M.L.H., van de Wall-Neecke, B.J., Hesselink, D.A., Becker, J.U., and Clahsen-van Groningen, M.C. (2020). Utility of immunohistochemistry with C3d in C3 glomerulopathy. Mod. Pathol. *33*, 431–439. https://doi.org/10.1038/s41379-019-0348-z.

40. Oto, O.A., Demir, E., Mirioglu, S., Dirim, A.B., Ozluk, Y., Cebeci, E., Basturk, T., Ucar, A.R., Soltanova, L., Nuriyev, K., et al. (2021). Clinical significance of glomerular C3 deposition in primary membranous nephropathy. J. Nephrol. *34*, 581–587. https://doi.org/10.1007/s40620-020-00915-w.

41. Giang, J., Seelen, M.A.J., van Doorn, M.B.A., Rissmann, R., Prens, E.P., and Damman, J. (2018). Complement Activation in Inflammatory Skin Diseases. Front. Immunol. *9*, 639. https://doi.org/10.3389/fimmu.2018.00639.

42. Heiderscheit, A.K., Hauer, J.J., and Smith, R.J.H. (2022). C3 glomerulopathy: Understanding an ultra-rare complement-mediated renal disease. Am. J. Med. Genet. C Semin. Med. Genet. *190*, 344–357. https://doi.org/10.1002/ajmg.c.31986.

43. Hou, J., Ren, K.Y.M., and Haas, M. (2022). C3 Glomerulopathy: A Review with Emphasis on Ultrastructural Features. Glomerular Dis. *2*, 107–120. https://doi.org/10.1159/000524552.

44. Pickering, M.C., D'Agati, V.D., Nester, C.M., Smith, R.J., Haas, M., Appel, G.B., Alpers, C.E., Bajema, I.M., Bedrosian, C., Braun, M., et al. (2013). C3 glomerulopathy: consensus report. Kidney Int. *84*, 1079–1089. https://doi.org/10.1038/ki.2013.377.

45. Thurman, J.M., Kulik, L., Orth, H., Wong, M., Renner, B., Sargsyan, S.A., Mitchell, L.M., Hourcade, D.E., Hannan, J.P., Kovacs, J.M., et al. (2013). Detection of complement activation using monoclonal antibodies against C3d. J. Clin. Invest. *123*, 2218–2230. https://doi.org/10.1172/JCI65861.

46. Fahnoe, K.C., Liu, F., Morgan, J.G., Ryan, S.T., Storek, M., Stark, E.G., Taylor, F.R., Holers, V.M., Thurman, J.M., Wawersik, S., et al. (2022). Development and Optimization of Bifunctional Fusion Proteins to Locally Modulate Complement Activation in Diseased Tissue. Front. Immunol. *13*, 869725. https://doi.org/10.3389/fimmu.2022.869725.

47. Risitano, A.M., Notaro, R., Pascariello, C., Sica, M., del Vecchio, L., Horvath, C.J., Fridkis-Hareli, M., Selleri, C., Lindorfer, M.A., Taylor, R.P., et al. (2012). The complement receptor 2/factor H fusion protein TT30 protects paroxysmal nocturnal hemoglobinuria erythrocytes from complement-mediated hemolysis and C3 fragment. Blood *119*, 6307–6316. https://doi.org/10.1182/blood-2011-12-398792.

48. Ruseva, M.M., Peng, T., Lasaro, M.A., Bouchard, K., Liu-Chen, S., Sun, F., Yu, Z.X., Marozsan, A., Wang, Y., and Pickering, M.C. (2016). Efficacy of Targeted

Complement Inhibition in Experimental C3 Glomerulopathy. J. Am. Soc. Nephrol. *27*, 405–416. https://doi.org/10.1681/ASN.2014121195.

49. Lundberg, B. (2014). Safety and Pharmacokinetics of TT30 in Subjects with Paroxysmal Nocturnal Hemoglobinuria (PNH). NCT01335165.

50. Tortajada, A., Montes, T., Martínez-Barricarte, R., Morgan, B.P., Harris, C.L., and de Córdoba, S.R. (2009). The disease-protective complement factor H allotypic variant Ile62 shows increased binding affinity for C3b and enhanced cofactor activity. Hum. Mol. Genet. *18*, 3452–3461. https://doi.org/10.1093/hmg/ddp289.

51. Yang, Y., Denton, H., Davies, O.R., Smith-Jackson, K., Kerr, H., Herbert, A.P., Barlow, P.N., Pickering, M.C., and Marchbank, K.J. (2018). An Engineered Complement Factor H Construct for Treatment of C3 Glomerulopathy. J. Am. Soc. Nephrol. *29*, 1649–1661. https://doi.org/10.1681/ASN.2017091006.

52. Pangburn, M.K., Schreiber, R.D., and Müller-Eberhard, H.J. (1977). Human complement C3b inactivator: isolation, characterization, and demonstration of an absolute requirement for the serum protein beta1H for cleavage of C3b and C4b in solution. J. Exp. Med. *146*, 257–270. https://doi.org/10.1084/jem.146.1.257.

53. Harrison, R.A., and Lachmann, P.J. (1980). Novel cleavage products of the third component of human complement. Mol. Immunol. *17*, 219–228. https://doi.org/10.1016/0161-5890(80)90074-7.

54. Persson, N., Johansson, M., Borsum, R., Witt, L., and Pramhed, A. (2019). Exploring Existing Human Complement C4d, TCC & Functional Activity Assays - Assessment of Complement Activation in Laborator. 17th European Meeting on Complement in Human Disease.

55. Kasprick, A., Holtsche, M.M., Rose, E.L., Hussain, S., Schmidt, E., Petersen, F., Panicker, S., and Ludwig, R.J. (2018). The Anti-C1s Antibody TNT003 Prevents Complement Activation in the Skin Induced by Bullous Pemphigoid Autoantibodies. J. Invest. Dermatol. *138*, 458–461. https://doi.org/10.1016/j.jid.2017.08.030.

56. Pickering, M.C., Cook, H.T., Warren, J., Bygrave, A.E., Moss, J., Walport, M.J., and Botto, M. (2002). Uncontrolled C3 activation causes membranoproliferative glomerulonephritis in mice deficient in complement factor H. Nat. Genet. *31*, 424–428. https://doi.org/10.1038/ng912.

57. Fakhouri, F., de Jorge, E.G., Brune, F., Azam, P., Cook, H.T., and Pickering, M.C. (2010). Treatment with human complement factor H rapidly reverses renal complement deposition in factor H-deficient mice. Kidney Int. *78*, 279–286. https://doi.org/10.1038/ki.2010.132.

58. Nichols, E.M., Barbour, T.D., Pappworth, I.Y., Wong, E.K.S., Palmer, J.M., Sheerin, N.S., Pickering, M.C., and Marchbank, K.J. (2015). An extended mini-complement factor H molecule ameliorates experimental C3 glomerulopathy. Kidney Int. *88*, 1314–1322. https://doi.org/10.1038/ki.2015.233.

59. Gilmore, A.C., Zhang, Y., Cook, H.T., Lavin, D.P., Katti, S., Wang, Y., Johnson, K.K., Kim, S., and Pickering, M.C. (2021). Complement activity is regulated in C3 glomerulopathy by IgG-factor H fusion proteins with and without properdin targeting domains. Kidney Int. *99*, 396–404. https://doi.org/10.1016/j.kint.2020.09.028.

60. Hammerberg, C., Katiyar, S.K., Carroll, M.C., and Cooper, K.D. (1998). Activated complement component 3 (C3) is required for ultraviolet induction of immunosuppression and antigenic tolerance. J. Exp. Med. *187*, 1133–1138. https://doi.org/10.1084/jem.187.7.1133.

61. Yoshida, Y., Kang, K., Berger, M., Chen, G., Gilliam, A.C., Moser, A., Wu, L., Hammerberg, C., and Cooper, K.D. (1998). Monocyte induction of IL-10 and down-regulation of IL-12 by iC3b deposited in ultraviolet-exposed human skin. J. Immunol. *161*, 5873–5879.

62. Salant, D.J., and Cybulsky, A.V. (1988). Experimental glomerulonephritis. Methods Enzymol. *162*, 421–461. https://doi.org/10.1016/0076-6879(88)62096-9.

63. Salant, D.J., Quigg, R.J., and Cybulsky, A.V. (1989). Heymann nephritis: mechanisms of renal injury. Kidney Int. *35*, 976–984. https://doi.org/10.1038/ki.1989.81.

64. Salant, D.J., Belok, S., Madaio, M.P., and Couser, W.G. (1980). A new role for complement in experimental membranous nephropathy in rats. J. Clin. Invest. *66*, 1339–1350. https://doi.org/10.1172/JCI109987.

65. Saran, A.M., Yuan, H., Takeuchi, E., McLaughlin, M., and Salant, D.J. (2003). Complement mediates nephrin redistribution and actin dissociation in experimental membranous nephropathy. Kidney Int. *64*, 2072–2078. https://doi.org/10.1046/j.1523-1755.2003.00305.x.

66. Schubart, A., Anderson, K., Mainolfi, N., Sellner, H., Ehara, T., Adams, C.M., Mac Sweeney, A., Liao, S.M., Crowley, M., Littlewood-Evans, A., et al. (2019). Small-molecule factor B inhibitor for the treatment of complement-mediated diseases. Proc. Natl. Acad. Sci. USA *116*, 7926–7931. https://doi.org/10.1073/pnas.1820892116.

67. Beck, L.H., Jr., and Salant, D.J. (2014). Membranous nephropathy: from models to man. J. Clin. Invest. *124*, 2307–2314. https://doi.org/10.1172/JCI72270.

68. Morgan, B.P., Boyd, C., and Bubeck, D. (2017). Molecular cell biology of complement membrane attack. Semin. Cell Dev. Biol. *72*, 124–132. https://doi.org/10.1016/j.semcdb.2017.06.009.

69. Koopman, J.J.E., van Essen, M.F., Rennke, H.G., de Vries, A.P.J., and van Kooten, C. (2020). Deposition of the Membrane Attack Complex in Healthy and Diseased Human Kidneys. Front. Immunol. *11*, 599974. https://doi.org/10.3389/fimmu.2020.599974.

70. Pruchno, C.J., Burns, M.W., Schulze, M., Johnson, R.J., Baker, P.J., and Couser, W.G. (1989). Urinary excretion of C5b-9 reflects disease activity in passive Heymann nephritis. Kidney Int. *36*, 65–71. https://doi.org/10.1038/ki.1989.162.

71. Schulze, M., Donadio, J.V., Jr., Pruchno, C.J., Baker, P.J., Johnson, R.J., Stahl, R.A., Watkins, S., Martin, D.C., Wurzner, R., Gotze, O., et al. (1991). Elevated urinary excretion of the C5b-9 complex in membranous nephropathy. Kidney Int. *40*, 533–538. https://doi.org/10.1038/ki.1991.242.

72. Kon, S.P., Coupes, B., Short, C.D., Solomon, L.R., Raftery, M.J., Mallick, N.P., and Brenchley, P.E. (1995). Urinary C5b-9 excretion and clinical course in idiopathic human membranous nephropathy. Kidney Int. *48*, 1953–1958. https://doi.org/10.1038/ki.1995.496.

73. Onda, K., Ohsawa, I., Ohi, H., Tamano, M., Mano, S., Wakabayashi, M., Toki, A., Horikoshi, S., Fujita, T., and Tomino, Y. (2011). Excretion of complement proteins and its activation marker C5b-9 in IgA nephropathy in relation to renal function. BMC Nephrol. *12*, 64. https://doi.org/10.1186/1471-2369-12-64.

74. Cochrane, C.G., Müller-Eberhard, H.J., and Aikin, B.S. (1970). Depletion of plasma complement in vivo by a protein of cobra venom: its effect on various immunologic reactions. J. Immunol. *105*, 55–69.

75. Pryjma, J., and Humphrey, J.H. (1975). Prolonged C3 depletion by cobra venom factor in thymus-deprived mice and its implication for the role of C3 as an essential second signal for B-cell triggering. Immunology *28*, 569–576.

76. Ing, M., Hew, B.E., Fritzinger, D.C., Delignat, S., Lacroix-Desmazes, S., Vogel, C.W., and Rayes, J. (2018). Absence of a neutralizing antibody response to humanized cobra venom factor in mice. Mol. Immunol. *97*, 1–7. https://doi.org/10.1016/j.molimm.2018.02.018.

77. Kulik, L., Laskowski, J., Renner, B., Woolaver, R., Zhang, L., Lyubchenko, T., You, Z., Thurman, J.M., and Holers, V.M. (2019). Targeting the Immune Complex-Bound Complement C3d Ligand as a Novel Therapy for Lupus. J. Immunol. *203*, 3136–3147. https://doi.org/10.4049/jimmunol.1900620.

78. Møller Rasmussen, J., Jepsen, H.H., Teisner, B., Holmskov-Nielsen, U., Rasmussen, G.G., and Svehag, S.E. (1989). Quantification by ELISA of erythrocyte-bound C3 fragments expressing C3d and/or C3c epitopes in patients with factor I deficiency and with autoimmune diseases. Vox Sang. *56*, 262–269. https://doi.org/10.1111/j.1423-0410.1989.tb02039.x.

79. Peffault de Latour, R., Fremeaux-Bacchi, V., Porcher, R., Xhaard, A., Rosain, J., Castaneda, D.C., Vieira-Martins, P., Roncelin, S., Rodriguez-Otero, P., Plessier, A., et al. (2015). Assessing complement blockade in patients with paroxysmal nocturnal hemoglobinuria receiving eculizumab. Blood *125*, 775–783. https://doi.org/10.1182/blood-2014-03-560540.

80. Freedman, J., and Massey, A. (1979). Complement components detected on normal red blood cells taken into EDTA and CPD. Vox Sang. *37*, 1–8. https://doi.org/10.1111/j.1423-0410.1979.tb02261.x.

81. Bu, F., Meyer, N.C., Zhang, Y., Borsa, N.G., Thomas, C., Nester, C., and Smith, R.J.H. (2015). Soluble c5b-9 as a biomarker for complement activation in atypical hemolytic uremic syndrome. Am. J. Kidney Dis. *65*, 968–969. https://doi.org/10.1053/j.ajkd.2015.02.326.

82. Valencia, C.M., Hersh, A.R., Burwick, R.M., Velásquez, J.A., Gutiérrez-Marín, J., Edna, F., Silva, J.L., Trujillo-Otálvaro, J., Vargas-Rodríguez, J., Bernal, Y., et al. (2022). Soluble concentrations of the terminal complement complex C5b-9 correlate

with end-organ injury in preeclampsia. Pregnancy Hypertens. *29*, 92–97. https://doi.org/10.1016/j.preghy.2022.07.001.

83. Wijaya, C., Burns, C., Hall, S., Farmer, M., Jones, D., Rowlandson, M., Choi, P., Formby, M., and de Malmanche, T. (2023). Measurement of complement activation via plasma soluble C5b-9 comparison with terminal complement complex staining in a series of kidney biopsies. Kidney Blood Press. Res. *48*, 220–230. https://doi.org/10.1159/000529734.

84. Costabile, M. (2010). Measuring the 50% haemolytic complement (CH50) activity of serum. J. Vis. Exp. *37*, 1923. https://doi.org/10.3791/1923.

YMTHE, Volume *32*

Supplemental Information

C3d-Targeted factor H inhibits tissue complement in disease models and reduces glomerular injury without affecting circulating complement

Fei Liu, Sarah T. Ryan, Kelly C. Fahnoe, Jennifer G. Morgan, Anne E. Cheung, Michael J. Storek, Alejandro Best, Hui A. Chen, Monica Locatelli, Shuyun Xu, Enno Schmidt, Leon F. Schmidt-Jiménez, Katja Bieber, Joel M. Henderson, Christine G. Lian, Admar Verschoor, Ralf J. Ludwig, Ariela Benigni, Giuseppe Remuzzi, David J. Salant, Susan L. Kalled, Joshua M. Thurman, V. Michael Holers, Shelia M. Violette, and Stefan Wawersik

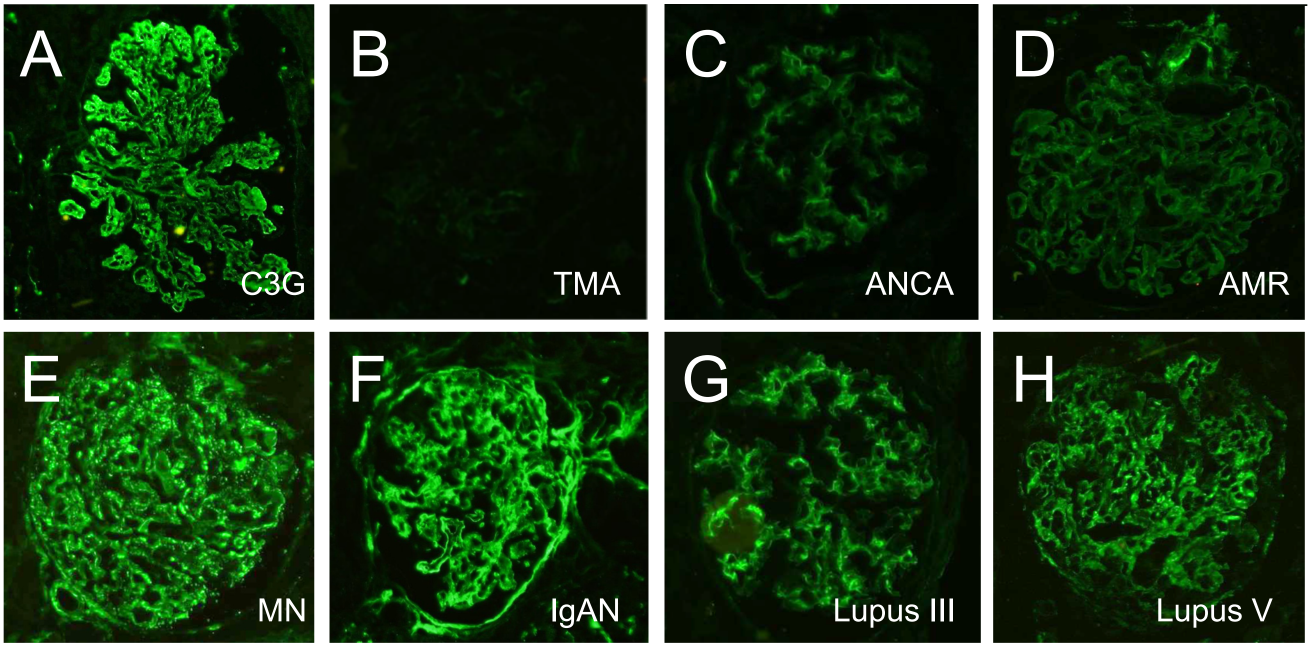

Figure S1. C3d Target Deposition in Human Glomerular Disease. Representative glomerular immunofluorescence in human biopsies from (A) C3 glomerulopathy (C3G), (B) Thrombotic microangiopathy (TMA), (C) anti-neutrophilic cytoplasmic autoantibody vasculitis (ANCA), (D) Antibody mediated rejection of kidney transplant (AMR), (E) Membranous glomerulopathy (MN), (F) IgA nephropathy (IgAN), (G) Class III and (H) Class IV lupus nephritis.

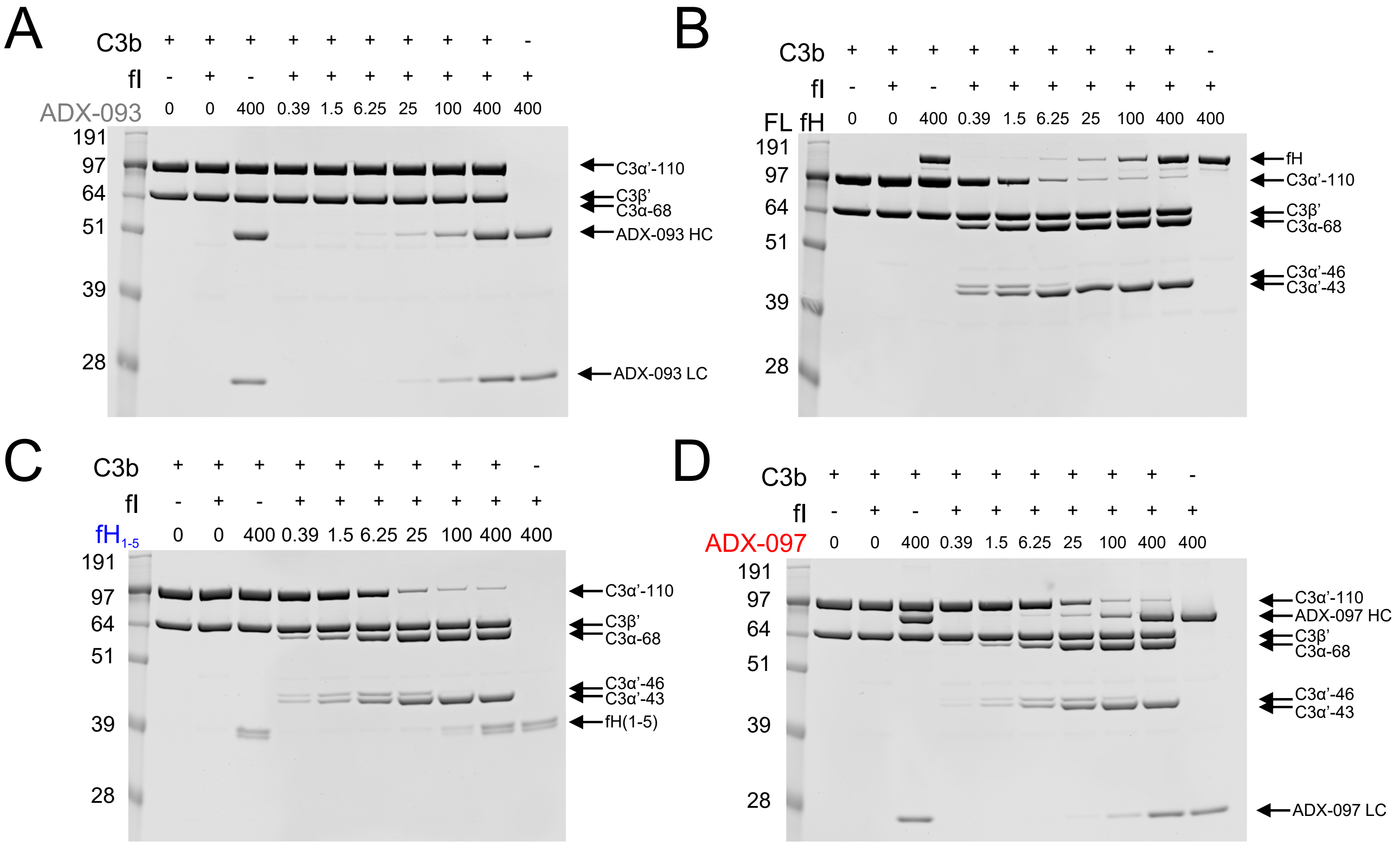

E

Concentration (nM)	% residual intact C3b			
	fH FL	ADX-097	fH1-5	ADX-093
0	100%	100%	100%	100%
0.39	71%	104%	95%	98%
1.56	44%	102%	86%	98%
6.25	16%	86%	60%	98%
25	11%	39%	21%	98%
100	9%	13%	14%	100%
400	6%	13%	13%	105%

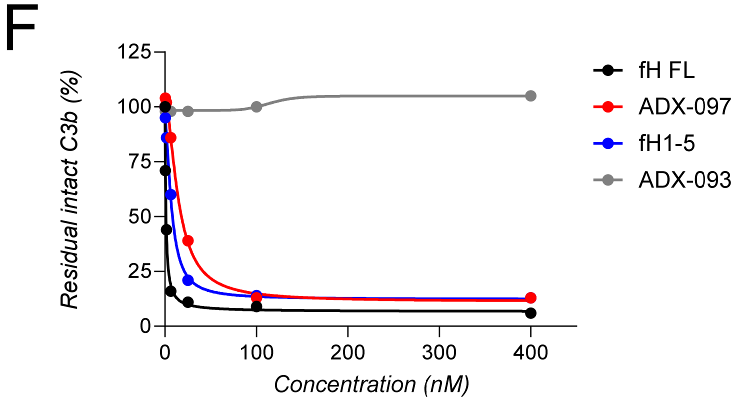

Figure S2. Effect of C3d-mAb-2fH on fI cofactor activity. C3b, fI, and increasing concentrations of (A) anti-C3d antibody (ADX-093), (B) full length Factor H (FL fH), (C) fH_{1-5}, or (D) human C3d-mAb-2fH (ADX-097) were incubated in solution at 37°C for 1 hour. C3b cleavage by fI was analyzed by SDS-PAGE followed by Coomassie staining. Disappearance of C3α'-110 band and the appearances of C3α'-68, -46, and -43 bands indicate C3b proteolytic inactivation. (E) % Residual C3b was calculated as the band intensities of the C3α'-110 band divided by the C3β' band, multiplied by 100. Band intensities were measured using imaging software. (F) % Residual C3b plotted as a function of fH (or control) concentration.

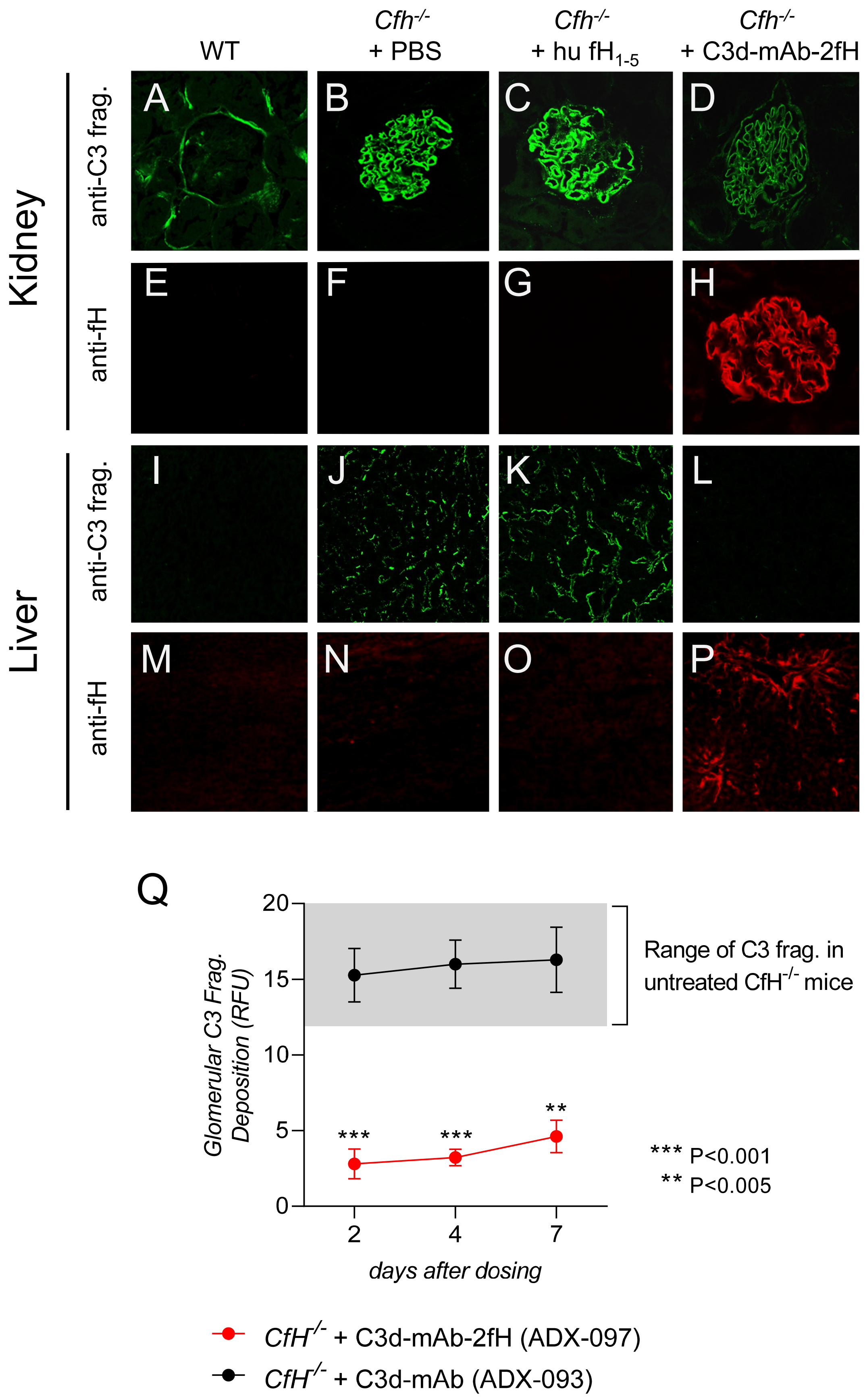

WT
Cfh-/- + PBS
Cfh-/- + hu fH1-5
Cfh-/- + C3d-mAb-2fH
Kidney
Liver
anti-C3 frag.
anti-fH
A
B
C
D
E
F
G
H
I
J
K
L
M
N
O
P
Q
Glomerular C3 Frag. Deposition (RFU)
days after dosing
Range of C3 frag. in untreated CfH-/- mice
*** P<0.001
** P<0.005
CfH-/- + C3d-mAb-2fH (ADX-097)
CfH-/- + C3d-mAb (ADX-093)

Figure S3. Tissue Localization and Complement Inhibition by C3d-mAb-2fH. A-P) *CfH*$^{-/-}$ mice were injected with PBS, 50 mg/kg IV ADX-048 (mouse/human chimeric C3d-mAb-2fH), or soluble human fH_{1-5} (16.6 mg/kg IV – 2x the molar equivalent to 50 mg/kg ADX-048 to provide similar number of fH_{1-5} molecules). Sections from kidney and liver tissue collected after 72 hours were immunostained for C3 active fragment (A-D and I-L) or for fH_{1-5} localization (anti-fH) (E-H and M-P). Background levels of C3 fragment deposition are detected in glomeruli (A) and liver (I) from wild-type mice, while increased C3 fragment deposition was detected in *CfH*$^{-/-}$ mice treated with PBS (B, J). C3 fragment deposition in kidney (C) or liver (K) was unaffected in mice treated with soluble human fH_{1-5}. However, C3 fragment deposition was qualitatively reduced in mice treated with C3d-mAb-2fH (ADX-048) (D, L). Consistent with this, fH_{1-5} localization was evident in C3d-mAb-2fH-treated mice (H, P), while no anti-fH immunofluorescence was detected in WT mice (E, M) or in *CfH*$^{-/-}$ mice treated with PBS (F, G) or soluble human fH_{1-5} (N, O). (Q) A time course of C3d-mAb-2fH-mediated tissue complement inhibition was in *CfH*$^{-/-}$ mice treated with 5 mg/kg IV ADX-097 (human C3d-mAb-2fH) or anti-C3d antibody (ADX-093) (n = 5 mice per group at each time point). Kidneys from *CfH*$^{-/-}$ mice (n = 3) were collected to define the range of C3 fragment deposition in untreated glomeruli (shaded area = average C3 fragment deposition +/- SEM). Kidney tissue was collected at 2, 4 and 7 days after dosing and immunostained with anti-C3 fragment antibody. Glomerular immunofluorescence from at least 10 glomeruli per animal was measured by digital image quantitation. At all time-points, C3d-mAb-2fH treatment significantly ($P < 0.005$) inhibited glomerular C3 fragment deposition. No difference was observed between PBS- and C3d-mAb-treated *CfH*$^{-/-}$ mice.

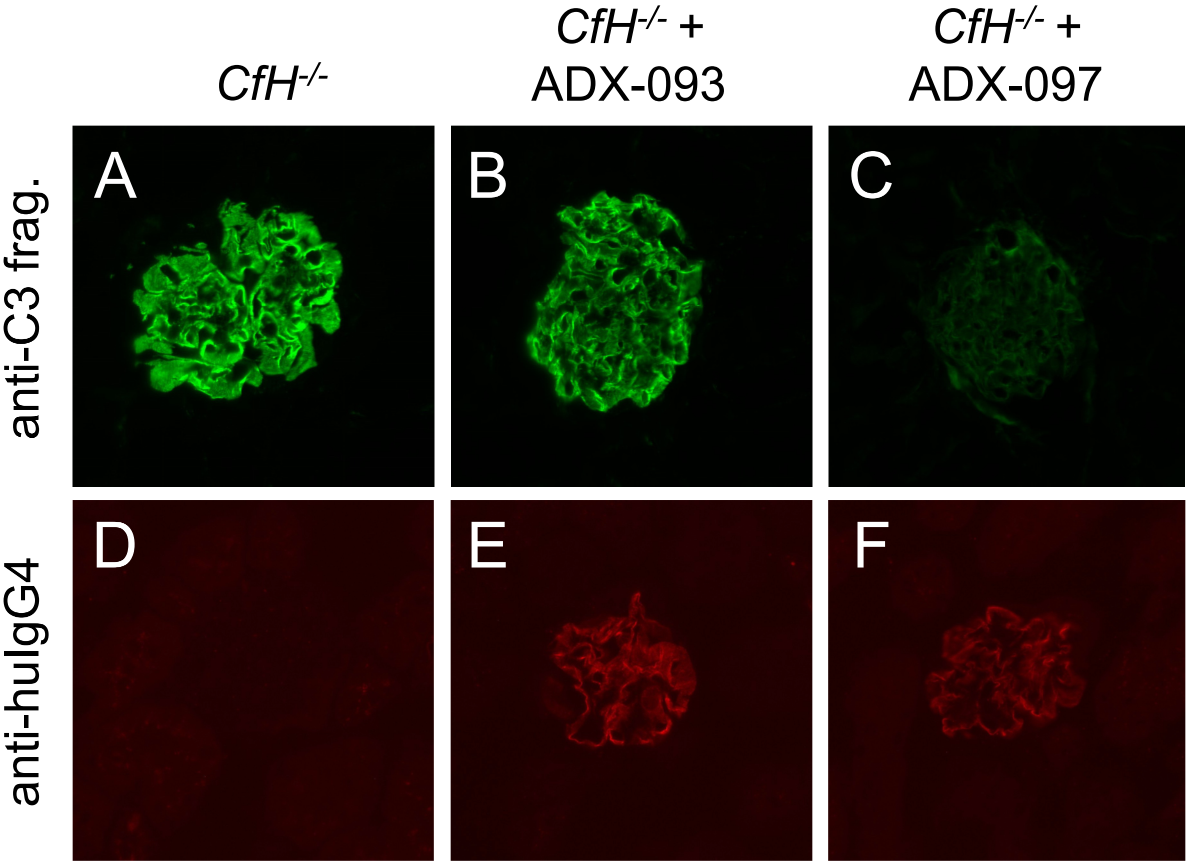

Figure S4. Localization and Tissue Complement Inhibition by ADX-097 and ADX-093 in CfH$^{-/-}$ Glomeruli. Representative images from kidneys immunostained with anti-C3 fragment antibodies. C3 fragment deposition was detected in untreated *CfH*$^{-/-}$ mice (A) and in *CfH*$^{-/-}$ mice treated with ADX-093 (B) or ADX-097 (C). anti-C3 fragment immunofluorescence images from all collected samples were used to generate quantitative data shown in Figure S4Q. To evaluate tissue drug localization, sections were immunostained with an anti-human IgG4 antibody that recognizes both ADX-093 and ADX-097. Representative immunofluorescence images are shown from untreated *CfH*$^{-/-}$ mice (D) and in *CfH*$^{-/-}$ mice treated with ADX-093 (E) or ADX-097 (F).

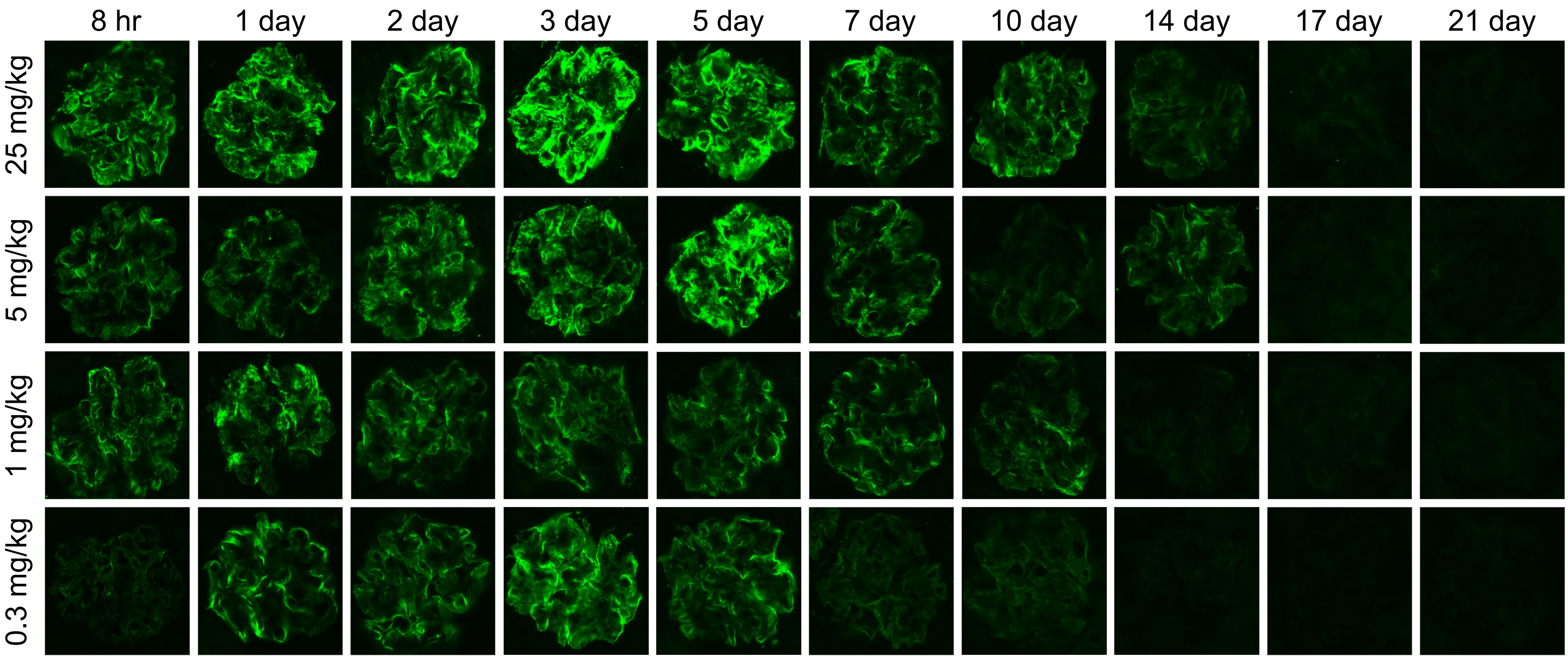

Figure S5. C3d-mAb-2fH Localization in *CfH$^{-/-}$* Glomeruli. Representative immunofluorescence of fH localization in C3d-mAb-2fH (ADX-118)-treated *CfH$^{-/-}$* mice. Images from all collected samples (n = 3-4 per time point) were used to generate quantitative data shown in Figure 4.

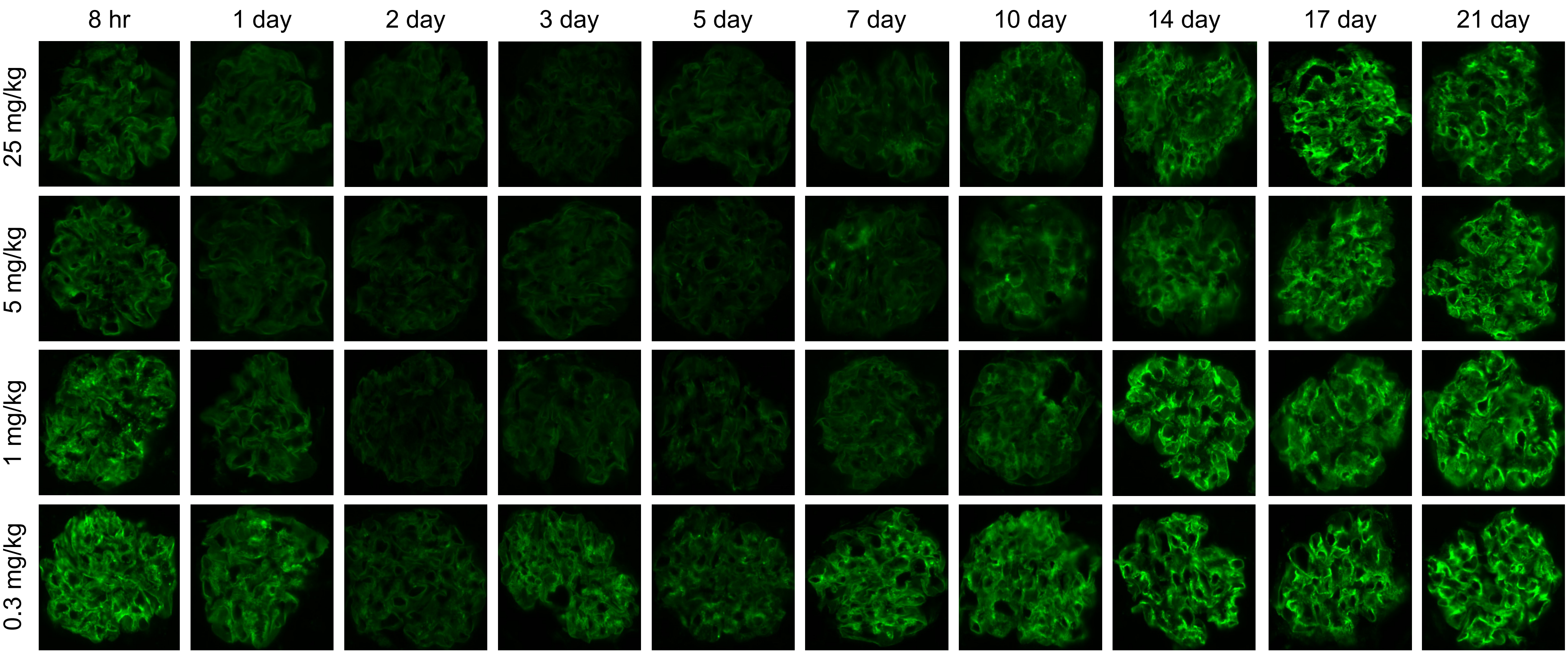

Figure S6. Tissue Complement Inhibition by C3d-mAb-2fH in *CfH$^{-/-}$* Glomeruli. Representative immunofluorescence of C3 fragment deposition (anti-C3c antibody) in C3d-mAb-2fH (ADX-118)-treated *CfH$^{-/-}$* mice. Images from all collected samples (n = 3-4 per time point) were used to generate quantitative data shown in Figure 4.

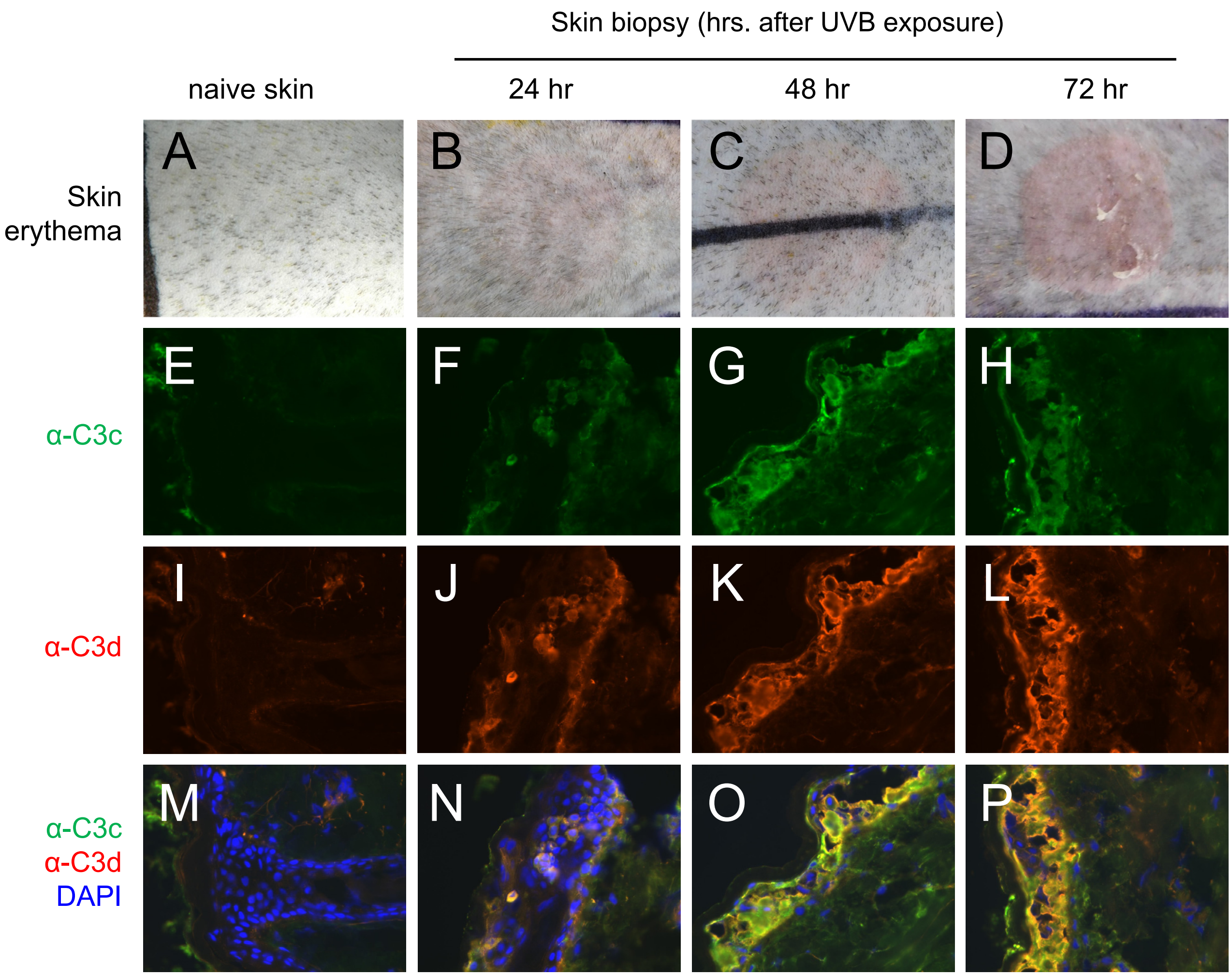

Figure S7. UVB-Induced Complement Activation in Cynomolgus Monkey Skin. Representative photographs of shaved cynomolgus monkey skin prior to exposure to UVB light (A) and 24 (B), 48 (C), and 72 hours (D) after UVB exposure. (E-H) anti-C3c immunofluorescence in naïve skin (E) and after UVB exposure (F-H) reveals the time course of complement activation in the model. (I-L) anti-C3d immunofluorescence shows minimal C3d deposition prior to UVB exposure (I). C3d is present in epidermis 24 hours after UVB exposure (J) and is maintained at 48 and 72 hours after exposure (K-L). anti-C3c (green) and anti-C3d (red) immunostaining co-localize in the epidermis (M-P)(biopsies were counterstained with DAPI (blue) to more clearly delineate tissue features.

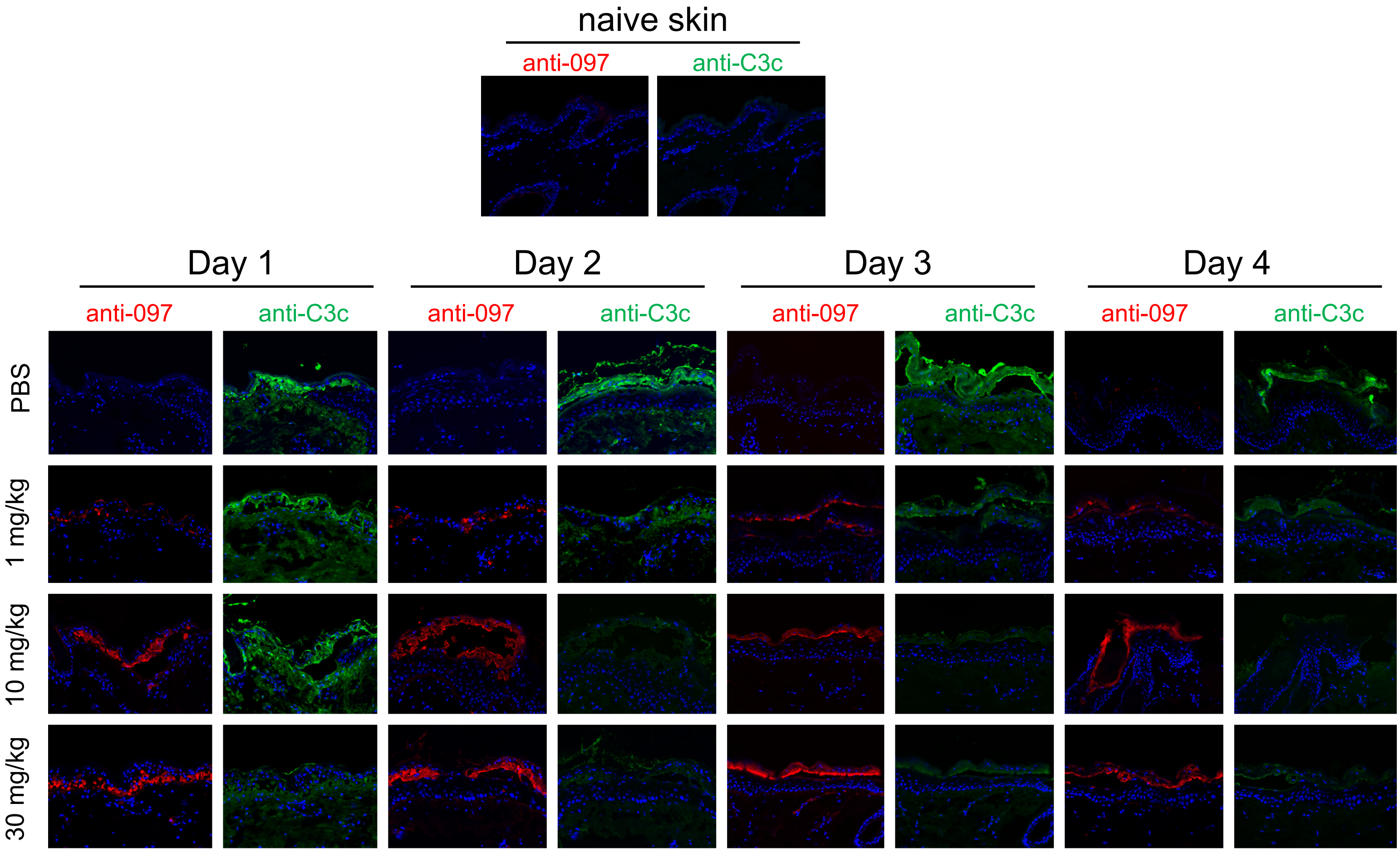

Figure S8. ADX-097 distributes to NHP skin and may inhibit local complement activation. Representative immunofluorescence of C3 fragment deposition (anti-C3c antibody, green) and C3d-mAb-2fH (ADX-097) localization (anti-fH immunostaining, red) in UVB-induced monkey skin. Images from all collected samples (n = 3 per time point) were used to generate quantitative data shown in Figure 3D, F.

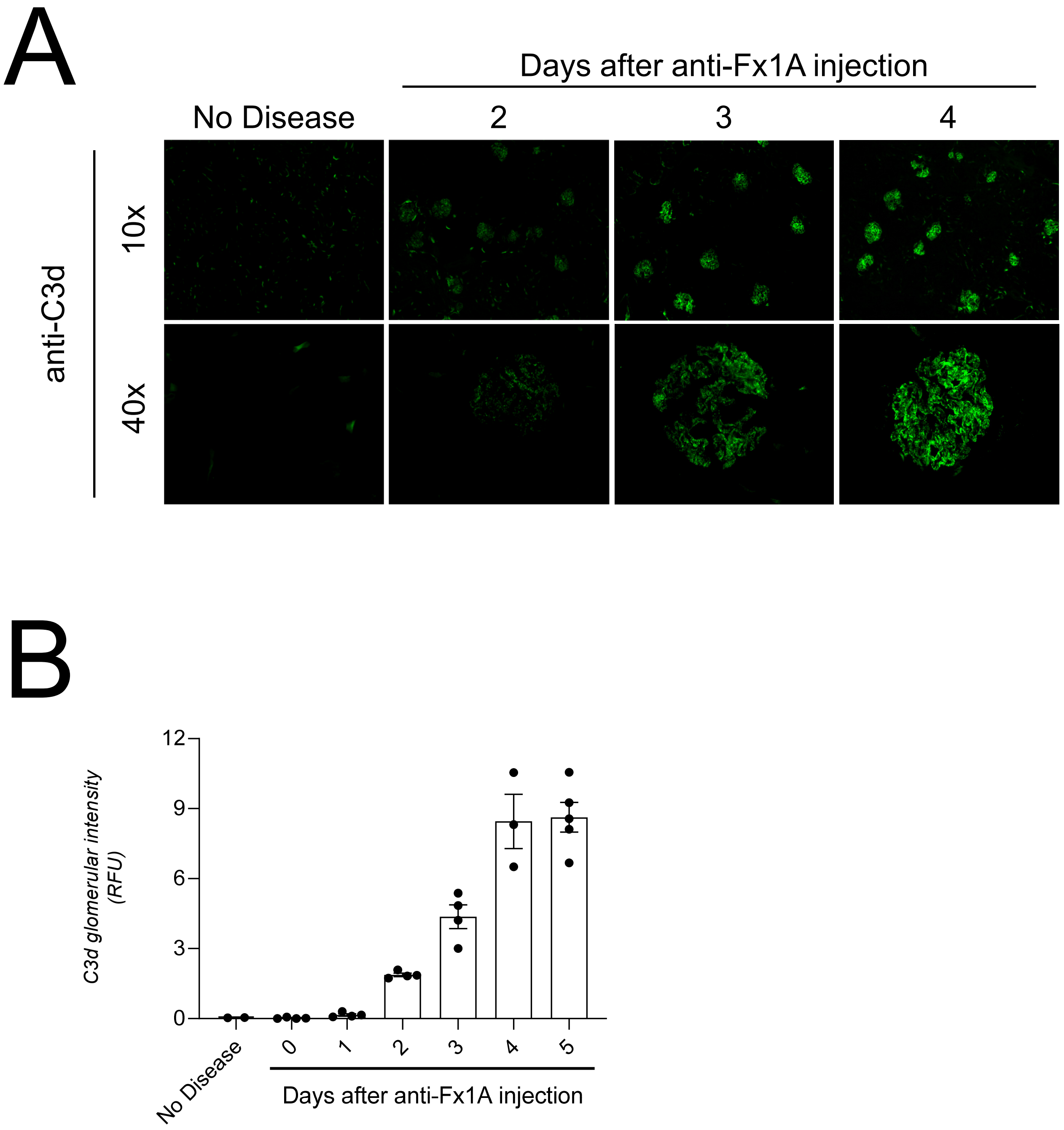

Figure S9. Characterization of C3d Deposition in Passive Heymann Nephritis. Kidney samples collected from the Passive Heymann Nephritis (PHN) model were collected and immunostained for C3d deposition. (A) Representative immunofluorescence from anti-C3d stained PHN kidneys shows clear C3d deposition in glomeruli by day 3 after anti-Fx1A-mediated disease induction. (B) Quantitation of C3d deposition in PHN glomeruli. Immunofluorescence from of least 10 glomeruli from 3-4 rats per time point was measured by digital image analysis.

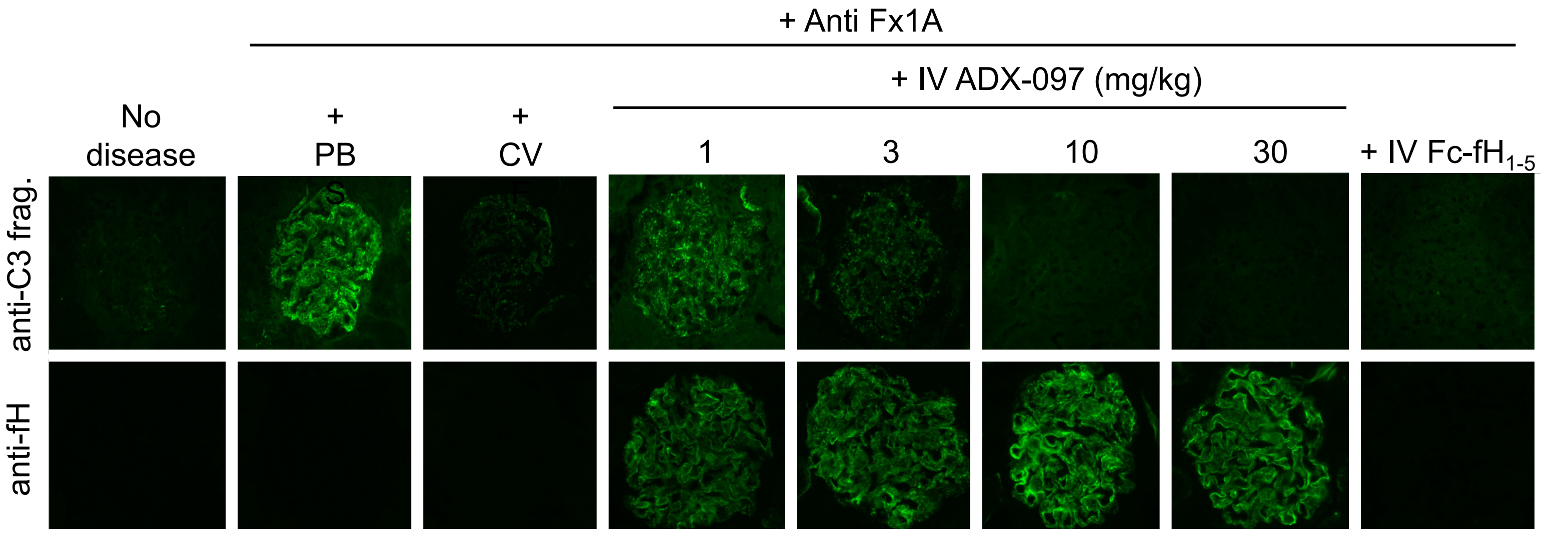

Figure S10. Representative Images of Tissue Complement Activity and ADX-097 Localization in Passive Heymann Nephritis. Images from all collected samples (n = 12 per group) were used to generate quantitative data shown in Figure 5C, D.

A

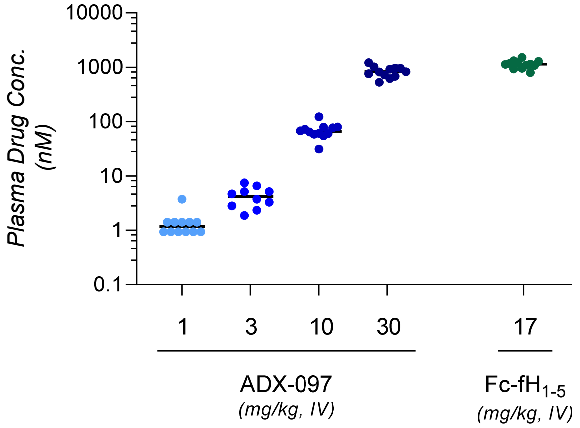

Treatment	Dose (mg/kg)	Ave Conc. (nM)	Ave Conc. (µg/ml)
ADX-097	30	837	181
ADX-097	10	69.0	14.9
ADX-097	3	4.31	0.93
ADX-097	1	1.36	0.29
Fc-2fH$_{1-5}$	17	1138	137

B

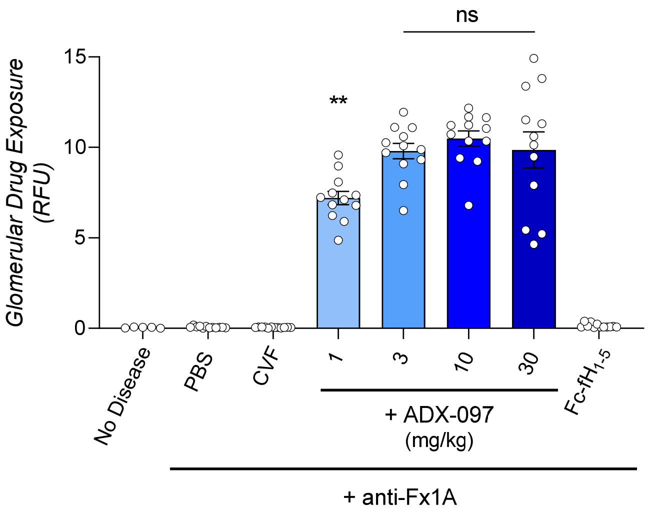

*** P < 0.004 vs. 3, 10, or 30 mg/kg ADX-097*

Figure S11. Tissue and Circulation Drug Exposure in Passive Heymann Nephritis. Kidney and plasma samples from the Passive Heymann Nephritis (PHN) study outlined in Figure 5A were collected on study day 5 (48 hours after ADX-097 treatment) analyzed for presence of ADX-097. (A) Plasma drug concentration was measured by drug-specific ELISA. Dose-dependent plasma drug concentrations are detected in the ADX-097 treatment groups. Fc-2fH$_{1-5}$ circulating concentrations are similar to the 30 mg/kg ADX-097 dose group, consistent with delivery of equimolar doses of the two proteins. A summary of circulating drug concentrations, expressed in nM and µg/ml, are below. (B) Quantitation of glomerular immunofluorescence using an anti-fH antibody to detect localization of ADX-097. No anti-fH immunofluorescence is detected in non-disease controls, nor in PHN + PBS, PHN + CVF, or PHN + Fc-2fH$_{1-5}$ dose groups. Dose-dependent localization of ADX-097 is detected in glomeruli, with less ADX-097 localization detected in glomeruli from the 1 mg/kg IV dose group. No difference in glomerular drug localization is observed between the 3, 10, and 30 mg/kg ADX-097 dose groups.

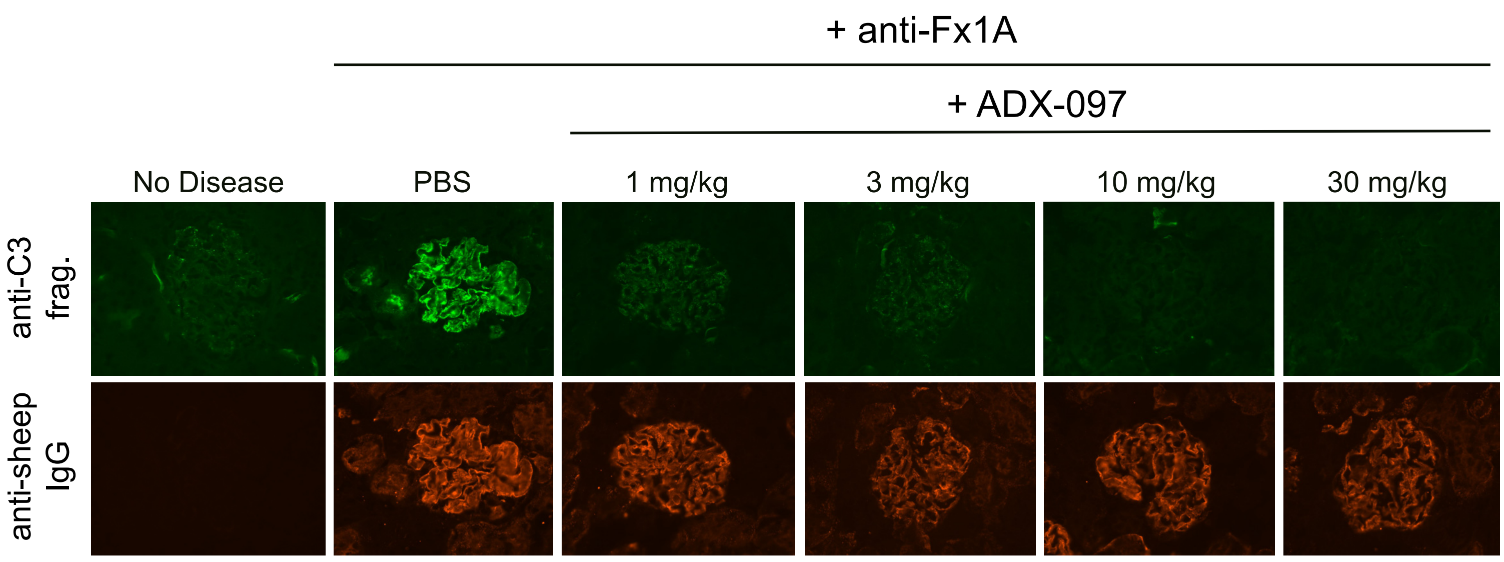

Figure S12. Anti-Sheep IgG Immunostaining in Passive Heymann Nephritis. Representative samples from Passive Heymann Nephritis (PHN) rats treated with indicated doses of C3d-mAb-2fH (ADX-097). Samples were co-stained for C3 fragment deposition (anti-C3c, green) and with an anti-sheep IgG antibody (red). ADX-097-mediated inhibition of C3 fragment deposition does not occur through blocking sheep anti-Fx1A, as anti-sheep IgG accumulation is similar in PHN + PBS controls and PHN + ADX-097-treated rats.

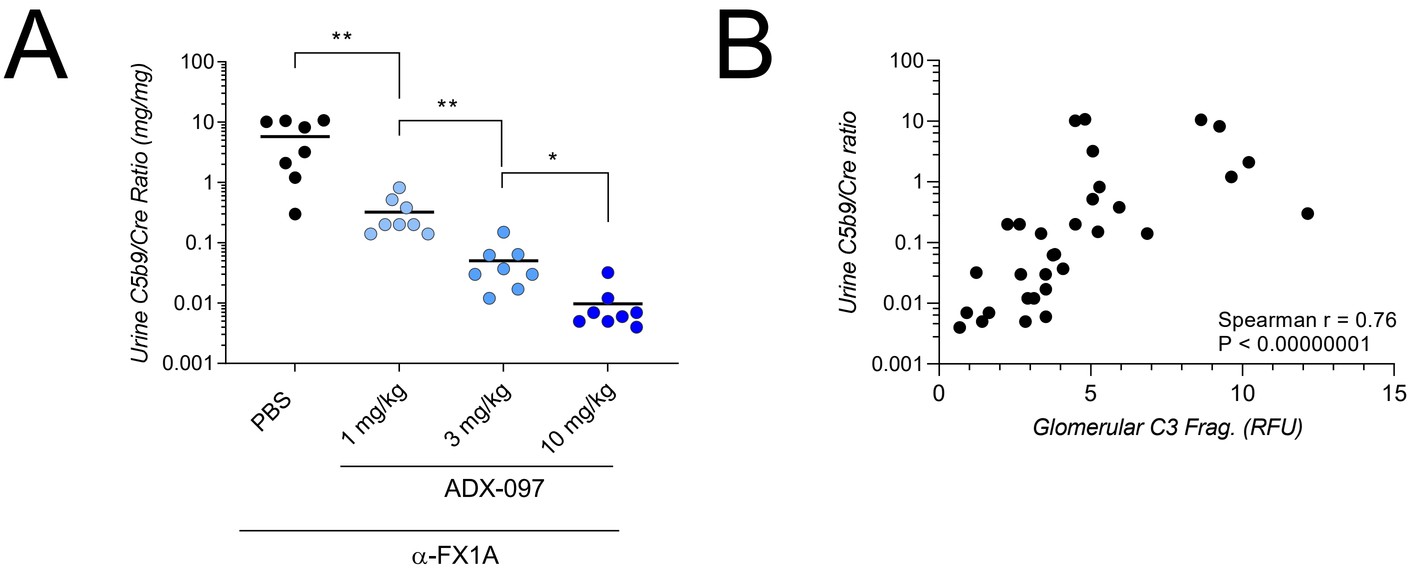

Figure S13. Urine C5b-9 Correlates with Glomerular Complement in Passive Heymann Nephritis. Soluble C5b-9 was measured in urine samples from study day 5 (48 hours after ADX-097 treatment) of the Passive Heymann Nephritis (PHN) study outlined in Figure 5A. (A) Urine C5b-9/Creatinine ratio is dose-dependently reduced in PHN rats after treatment with ADX-097 (**P < 0.002, * P < 0.03). Note that these doses (1 to 10 mg/kg, IV) do not inhibit circulating complement (see Figure 5D), suggesting that uC5b-9/Cre reflects changes in renal complement activity. (B) X-Y correlation plot of glomerular C3 fragment immunostaining on study day 5 vs. urine C5b-9/Cre ratio shows a strong correlation between glomerular complement deposition and urine C5b-9/Cre ratio (P < 0.00000001).

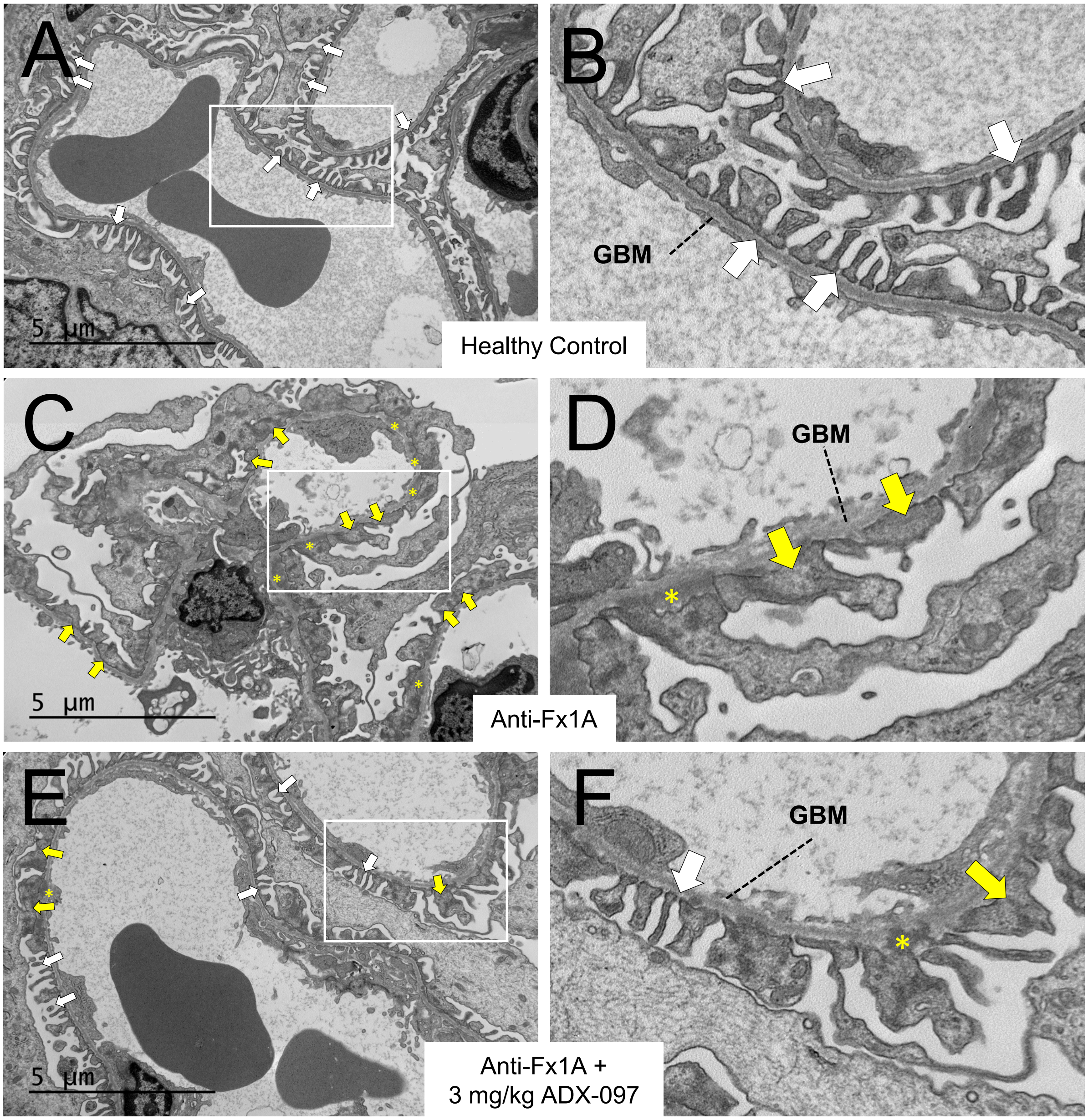

Figure S14. C3d-mAb-2fH Protects Podocyte Ultrastructure in Passive Heymann Nephritis. (A) Representative EM image from glomeruli in the healthy control (treated with normal serum) group shows well-differentiated podocyte foot processes (white arrows). (B) Enlarged image of the area outlined in the white frame in panel A. White arrows indicate examples of normal slit diaphragms. The glomerular basement membrane (GBM) is of uniform thickness with a distinct lamina densa (C) Representative glomerular EM image from a PHN rat shows extensive foot process effacement (yellow arrows), electron-dense regions consistent with immune complexes (yellow asterisks). (D) Enlarged image of the area outlined in the white box in panel B. Yellow arrows indicate effaced podocyte foot processes. Yellow asterisks denote electron-dense regions consistent with immune complex deposition. Note the distorted and thickened GBM without a clear lamina densa. (E) Glomerular EM from PHN rats treated with 3 mg/kg SC ADX-097 show substantial preservation of podocyte foot processes (white arrows), though occasional examples of effaced podocytes can be found (yellow arrows). (F) Enlarged image of the area within the white box in panel C. The white arrow highlights a representative healthy slit diaphragm, while the yellow arrow points out a partially effaced podocyte foot process. The GBM is more uniform in thickness and has a differentiated lamina densa.

A

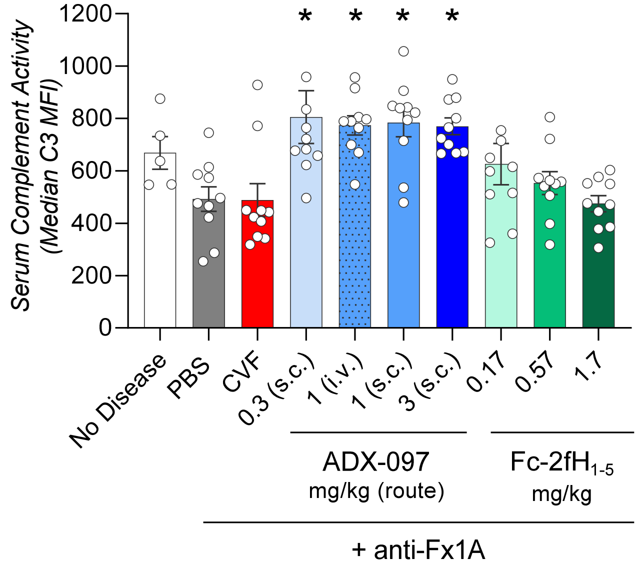

B

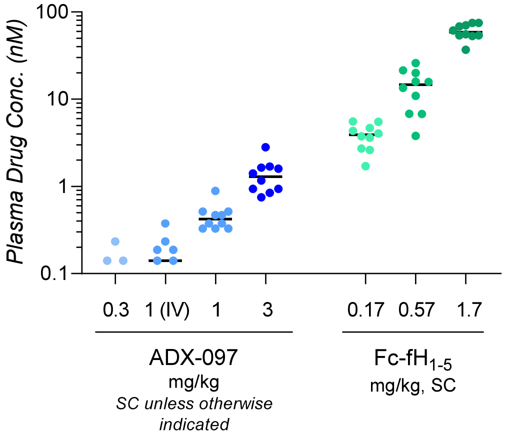

ADX-097 Dose (mg/kg)	Ave Plasma Conc. (nM)	Ave Plasma Conc. (µg/ml)	Fc-fH Dose (mg/kg)	Ave Plasma Conc. (nM)	Ave Plasma Conc. (µg/ml)
3	1.38	0.298	1.7	60.3	7.3
1	0.46	0.099	0.57	14.1	1.71
1 (IV)	0.16	0.035	-	-	-
0.3	0.12	0.026	0.17	3.85	0.465

Figure S15. Circulating Complement and Drug Exposure in Passive Heymann Nephritis. Kidney and plasma samples from the Passive Heymann Nephritis (PHN) study outlined in Figure 6A were collected on study day 7 (96 hours after ADX-097 treatment) analyzed for presence of ADX-097. (A) Day 7 serum complement activity measured by zymosan beads. The PHN + PBS control group shows a non-statistically significant reduction in serum complement activity compared to non-disease controls, suggesting that circulating complement levels may begin to drop as disease progresses. CVF- and Fc-2fH$_{1-5}$-treated PHN rats' serum complement activity is unchanged from PBS-treated controls. All doses of ADX-097 exhibit serum complement activity that is slightly higher than PHN + PBS controls ($P < 0.05$), indicating that ADX-097 is blocking glomerular complement activity without affecting systemic complement. (B) Plasma drug concentration was measured by drug-specific ELISA. Dose-dependent plasma drug concentrations are detected in the ADX-097 treatment groups. Fc-2fH$_{1-5}$ plasma concentrations are significantly higher at this time point, suggesting more rapid clearance of ADX-097 from circulation. A summary of circulating drug concentrations, expressed in nM and µg/ml, are below.

Supplemental Tables

Table S1. Semi-quantitative Scoring – Retrospective Review of Renal C3 Fragment Staining in Glomeruli

	TMA	ANCA	MN	IgAN	Lupus III	Lupus IV
Total Cases	100	104	109	107	112	107
# Positive	17	43	94	96	108	94
# Negative	83	61	15	11	4	13
% Positive	20.8	41.3	86.2	89.7	96.3	87.9
# of samples per positive score	9 = trace<br>2 = 1+<br>1 = 1.5+<br>4 = 1-2+<br>1 = 2-3+	13 = trace<br>9 = tr-1+<br>10 = 1+<br>7 = 1-2+<br>3 = 2+<br>1 = 3+	20 = trace<br>3 = tr-1+<br>32 = 1+<br>5 = 1-2+<br>21 = 2+<br>5 = 2-3+<br>9 = 3+	7 = trace<br>2 = tr-1+<br>31 = 1+<br>11 = 1-2+<br>31 = 2+<br>7 = 2-3+<br>6 = 3+	4 = trace<br>1 = tr-1+<br>24 = 1+<br>4 = 1-2+<br>42 = 2+<br>13 = 2-3+<br>20 = 3+	15 = trace<br>1 = tr-1+<br>27 = 1+<br>7 = 1-2+<br>24 = 2+<br>3 = 2-3+<br>17 = 3+

Table S2. Binding of Targeting Antibodies to C3d

Protein ID	Anti-C3d Antibody	Effector	Human C3d K_D (nM)	Cyno C3d K_D (nM)	Mouse C3d K_D (nM)
ADX-058 (3d8b)	Mouse (IgG1)	-	7.10	Not tested	6.90
ADX-093	Human IgG4 (humanized ADX-058)	-	10.0	Not tested	Not tested
ADX-118	ADX-058 (Mouse IgG1)	Mouse fH_{1-5}	9.40	1.33	7.16
ADX-097	ADX-093 (Human IgG4)	Human fH_{1-5}	12.0	6.32	3.15

Table S3. Comparison of C3d-mAb-fH Activity Across Species (Zymosan Assays)

Protein ID	Anti-C3d Antibody	Effector	Serum Species	IC_{50} (nM)
ADX-097	ADX-093 (Human IgG4)	2x Human fH_{1-5}	Mouse	202 ± 54
	ADX-093 (Human IgG4)	2x Human fH_{1-5}	Rat	99 ± 17
	ADX-093 (Human IgG4)	2x Human fH_{1-5}	Human	191 ± 17
ADX-118	ADX-058 (Mouse IgG1)	2x Mouse fH_{1-5}	Mouse	46 ± 3.9
	ADX-058 (Mouse IgG1)	2x Mouse fH_{1-5}	Rat	281 ± 52
	ADX-058 (Mouse IgG1)	2x Mouse fH_{1-5}	Human	No activity

Supplemental Methods

Humanization of 3d8b

The anti-C3d mAb 3d8b, originally identified as a murine IgG2, [42] was humanized as previously described [43] using modeled structure-based complementarity-determining region (CDR) grafting into human germline gene acceptor frameworks [81]. The resulting antibody is a hinge-stabilized (S228P) human IgG4 with additional Fc mutations to minimize C1q binding and effector function.

Generation and expression of fusion proteins

Anti-C3d parental antibodies, antibody, Fab, and CR2 fusions, and Fc-fusions were transiently transfected in CHO cells using standard methods. Proteins were affinity purified over Protein A (Cytiva, Marlborough, MA) and buffer exchanged into phosphate buffered saline (PBS) pH 7.4 using size exclusion chromatography to yield material with greater than 95% purity and endotoxin levels below 0.5 EU/mg. Proteins were concentration by centrifugation through high flow polyethersulfone (PES) membranes and sterile filtered through 0.2 µm filters.

Fluid phase fI co-factor activity

Complement regulatory activity of C3d-mAb-2fH was assayed in a fluid phase fI co-factor activity assay as previously described. [47,48] 0.7 µM of C3b and 20 nM of fI were mixed with either full length fH (FL fH), $fH_{1\text{-}5}$, ADX-097 (human C3d-mAb-2fH), or ADX-093 (human anti-C3d binding antibody) in a total volume of 16 µl in PBS buffer at 37°C for 30 min. The proteolytic breakdown of C3b was assessed using a 10% SDS-PAGE gel followed by Coomassie

staining. C3b cleavage was calculated by measuring the band intensity ratio of C3α'-110/C3β' using a LI-COR gel imager and associated software.

Zymosan Complement Activation Assay

Pre-activated zymosan (Complement Technologies, Tyler, TX) was diluted in PBS pH 7.4 containing 25 mM EGTA, 12.5 mM $MgCl_2$ and 0.1% BSA. Antibody fusion proteins were serially diluted in PBS pH 7.4, 0.1% BSA. Activated zymosan was combined with fusion protein and 25% complement preserved serum. Reactions were incubated at 37°C for 20 minutes and stopped by the addition of 50 mM EDTA. Samples were centrifuged (3000 rpm), and the zymosan pellet was resuspended and washed in PBS pH 7.4, 0.1% BSA followed by centrifugation. The final pellet was resuspended in goat anti mouse C3–fluorescein isothiocyanate (FITC) $F(ab')_2$ (MP Biomedicals, Solon, OH) in PBS, pH 7.4 and incubated for 1 hour on ice. Following washing and centrifugation as described above, the pellets were resuspended in PBS, pH 7.4, 0.1% BSA. Data were acquired on an Attune flow cytometer (ThermoFisher, Waltham, MA) and analysed in FlowJo (FlowJo, LLC, Ashland, OR).

Plasma drug exposure assays (C3d-mAb-2fH and Fc-2fH)

C3d-mAb-2fH proteins in plasma were measured by ELISA. Human C3d protein (Complement Technologies, Tyler TX) was coated onto plates, then blocked in 1% BSA/TBS-Tween. Standards were diluted into 2% mouse or rat plasma and study samples were diluted in TBS. Biotin conjugated anti-human Factor H antibody (OX-24-biotin, ThermoFisher, Waltham MA) was added and subsequently detected with streptavidin – HRP (BioLegend San Diego, CA). The reaction was stopped with 2 N Sulfuric Acid Stop Solution (R&D systems, Minneapolis, MN)

and plates were read on a SpectraMax 250 plate reader. A four-parameter fit was used to generate the standard curve and test samples values were extrapolated from the standard curve. Fc-2fH protein was measured in plasma by coating plates with anti-human Factor H antibody (OX-24, ThermoFisher, Waltham, MA) and blocked in 2% BSA/PBS. Standards and test samples were diluted into 2% rat plasma/PBS. Drug binding was detected using an HRP-anti human IgG4 Fc' (Abcam, Waltham, MA) followed by TMB (ThermoFisher, Waltham, MA), then stopped with stop solution (ThermoFisher, Waltham, MA) and read on a SpectraMax 250 instrument. Standards were fit to a four-parameter curves and test sample concentrations were extrapolated using GraphPad Prism software.

Transmission Electron Microscopy

Kidney tissue was fixed with 1% osmium tetroxide in 0.15M cacodylate buffer, dehydrated in an acetone series, and embedded in epoxy resin. Ultrathin sections were cut at 72 nm with a diamond knife, mounted on 200-mesh copper grids, and stained with 4% uranyl acetate and 0.4% lead citrate. Prepared sections were examined in a JEOL JEM-1010 transmission electron microscope, and digital images were collected at a range of magnifications with an Erlangshen ES100W digital camera (Gatan, Pleasanton,CA).
